# Neuronal Hyperactivity Disturbs ATP Microgradients, Impairs Microglial Motility, and Reduces Phagocytic Receptor Expression Triggering Apoptosis/Microglial Phagocytosis Uncoupling

**DOI:** 10.1371/journal.pbio.1002466

**Published:** 2016-05-26

**Authors:** Oihane Abiega, Sol Beccari, Irune Diaz-Aparicio, Agnes Nadjar, Sophie Layé, Quentin Leyrolle, Diego Gómez-Nicola, María Domercq, Alberto Pérez-Samartín, Víctor Sánchez-Zafra, Iñaki Paris, Jorge Valero, Julie C. Savage, Chin-Wai Hui, Marie-Ève Tremblay, Juan J. P. Deudero, Amy L. Brewster, Anne E. Anderson, Laura Zaldumbide, Lara Galbarriatu, Ainhoa Marinas, Maria dM. Vivanco, Carlos Matute, Mirjana Maletic-Savatic, Juan M. Encinas, Amanda Sierra

**Affiliations:** 1 Achucarro Basque Center for Neuroscience, Bizkaia Science and Technology Park, Zamudio, Spain; 2 University of the Basque Country, Leioa, Spain; 3 Université Bordeaux Segalen, Bordeaux, France; 4 Centre for Biological Sciences, University of Southampton, Southampton, United Kingdom; 5 Ikerbasque Foundation, Bilbao, Spain; 6 Centre de recherche du CHU de Québec, Axe Neurosciences, Québec, Canada; 7 Université Laval, Département de médecine moléculaire, Québec, Canada; 8 Baylor College of Medicine, The Jan and Dan Duncan Neurological Research Institute at Texas Children’s Hospital, Houston, Texas, United States of America; 9 University Hospital of Cruces, Bilbao, Spain; 10 CIC BioGUNE, Derio, Spain; Stanford University School of Medicine, UNITED STATES

## Abstract

Phagocytosis is essential to maintain tissue homeostasis in a large number of inflammatory and autoimmune diseases, but its role in the diseased brain is poorly explored. Recent findings suggest that in the adult hippocampal neurogenic niche, where the excess of newborn cells undergo apoptosis in physiological conditions, phagocytosis is efficiently executed by surveillant, ramified microglia. To test whether microglia are efficient phagocytes in the diseased brain as well, we confronted them with a series of apoptotic challenges and discovered a generalized response. When challenged with excitotoxicity in vitro (via the glutamate agonist NMDA) or inflammation in vivo (via systemic administration of bacterial lipopolysaccharides or by omega 3 fatty acid deficient diets), microglia resorted to different strategies to boost their phagocytic efficiency and compensate for the increased number of apoptotic cells, thus maintaining phagocytosis and apoptosis tightly coupled. Unexpectedly, this coupling was chronically lost in a mouse model of mesial temporal lobe epilepsy (MTLE) as well as in hippocampal tissue resected from individuals with MTLE, a major neurological disorder characterized by seizures, excitotoxicity, and inflammation. Importantly, the loss of phagocytosis/apoptosis coupling correlated with the expression of microglial proinflammatory, epileptogenic cytokines, suggesting its contribution to the pathophysiology of epilepsy. The phagocytic blockade resulted from reduced microglial surveillance and apoptotic cell recognition receptor expression and was not directly mediated by signaling through microglial glutamate receptors. Instead, it was related to the disruption of local ATP microgradients caused by the hyperactivity of the hippocampal network, at least in the acute phase of epilepsy. Finally, the uncoupling led to an accumulation of apoptotic newborn cells in the neurogenic niche that was due not to decreased survival but to delayed cell clearance after seizures. These results demonstrate that the efficiency of microglial phagocytosis critically affects the dynamics of apoptosis and urge to routinely assess the microglial phagocytic efficiency in neurodegenerative disorders.

## Abstract 2

Phagocytosis by microglia is tightly coupled to apoptosis, swiftly removing apoptotic cells and actively maintaining tissue homeostasis, but the neuronal hyperactivity associated with epilepsy disrupts the ATP gradients that drive phagocytosis, leading to the accumulation of apoptotic cells and inflammation.

## Author Summary

Phagocytosis, the engulfment and digestion of cellular debris, is at the core of the regenerative response of the damaged tissue, because it prevents the spillover of toxic intracellular contents and is actively anti-inflammatory. In the brain, the professional phagocytes are microglia, whose dynamic processes rapidly engulf and degrade cells undergoing apoptosis—programmed cell death—in physiological conditions. Thus, microglia hold the key to brain regeneration, but their efficiency as phagocytes in the diseased brain is only presumed. Here, we have discovered a generalized response of microglia to apoptotic challenge induced by excitotoxicity and inflammation, in which they boost their phagocytic efficiency to account for the increase in apoptosis. To our surprise, this apoptosis/microglial phagocytosis coupling was lost in the hippocampus from human and experimental mesial temporal lobe epilepsy (MTLE), a major neurodegenerative disorder characterized by excitotoxicity, inflammation, and seizures. This uncoupling was due to widespread ATP release during neuronal hyperactivity, which “blinded” microglia to the ATP microgradients released by apoptotic cells as “find-me” signals. The impairment of phagocytosis led to the accumulation of apoptotic cells and the build-up of a detrimental inflammatory reaction. Our data advocates for systematic assessment of the efficiency of microglial phagocytosis in brain disorders.

## Introduction

Phagocytosis is a crucial component of the regenerative response that is well described in peripheral inflammatory diseases, in which macrophages must rapidly clear the cell corpses to prevent the spillover of toxic intracellular contents and the initiation of an inflammatory response [1]. In the brain, however, where neuronal apoptosis (programmed cell death) is ubiquitous during development as well as in neurodegenerative diseases such as epilepsy, ischemia/stroke, Alzheimer and Parkinson diseases, or multiple sclerosis, phagocytosis is known to be executed largely by microglia but remains notoriously unexplored [2].

Microglia belong to the macrophage–monocyte lineage but, unlike most tissue-resident macrophages, they are derived from the embryonic yolk sac and invade the brain parenchyma early during embryonic development [3]. They also have other exclusive characteristics such as the unique motility of their fine processes, which scan the whole brain parenchyma every few hours [4,5]. As a result of these recent findings, novel roles of microglia in the healthy brain have just begun to be unraveled, including their capacity to interact with neurons and modulate their activity [6–8]. They also shape the adult hippocampal neurogenic niche by removing the excess of newborn cells naturally undergoing apoptosis [9]. Here, microglia are very skilled phagocytes that rapidly engulf and degrade the apoptotic cells. Importantly, in physiological conditions only a small proportion of microglia are in the process of phagocytosing at a given time, suggesting that they hold a large phagocytic reservoir that could be summoned in the diseased brain.

To test whether microglia are efficient phagocytes in neurodegenerative conditions, we confronted them with a series of apoptotic challenges evoked by excitotoxicity as well as acute or chronic inflammation. We discovered that, in these conditions, hippocampal microglia raised their phagocytic capacity to proportionally match the increase in apoptosis. Thus, a general mechanism of phagocytic response begins to emerge, in which microglial phagocytosis is efficiently coupled to apoptosis. Unexpectedly, we found that the microglial phagocytic response was impaired in both human and experimental mesial temporal lobe epilepsy (MTLE), a major neurological disorder characterized by seizures, excitotoxicity, and inflammation. Herein, we show that the uncoupling between microglial phagocytosis and apoptosis is the result of an impairment of microglial motility and apoptotic cell recognition; it is related to the disruption of ATP microgradients; and leads to a delayed clearance of apoptotic cells and inflammation.

## Results

### Microglial Phagocytosis Is Coupled to Cell Apoptosis

**Fig 1:**
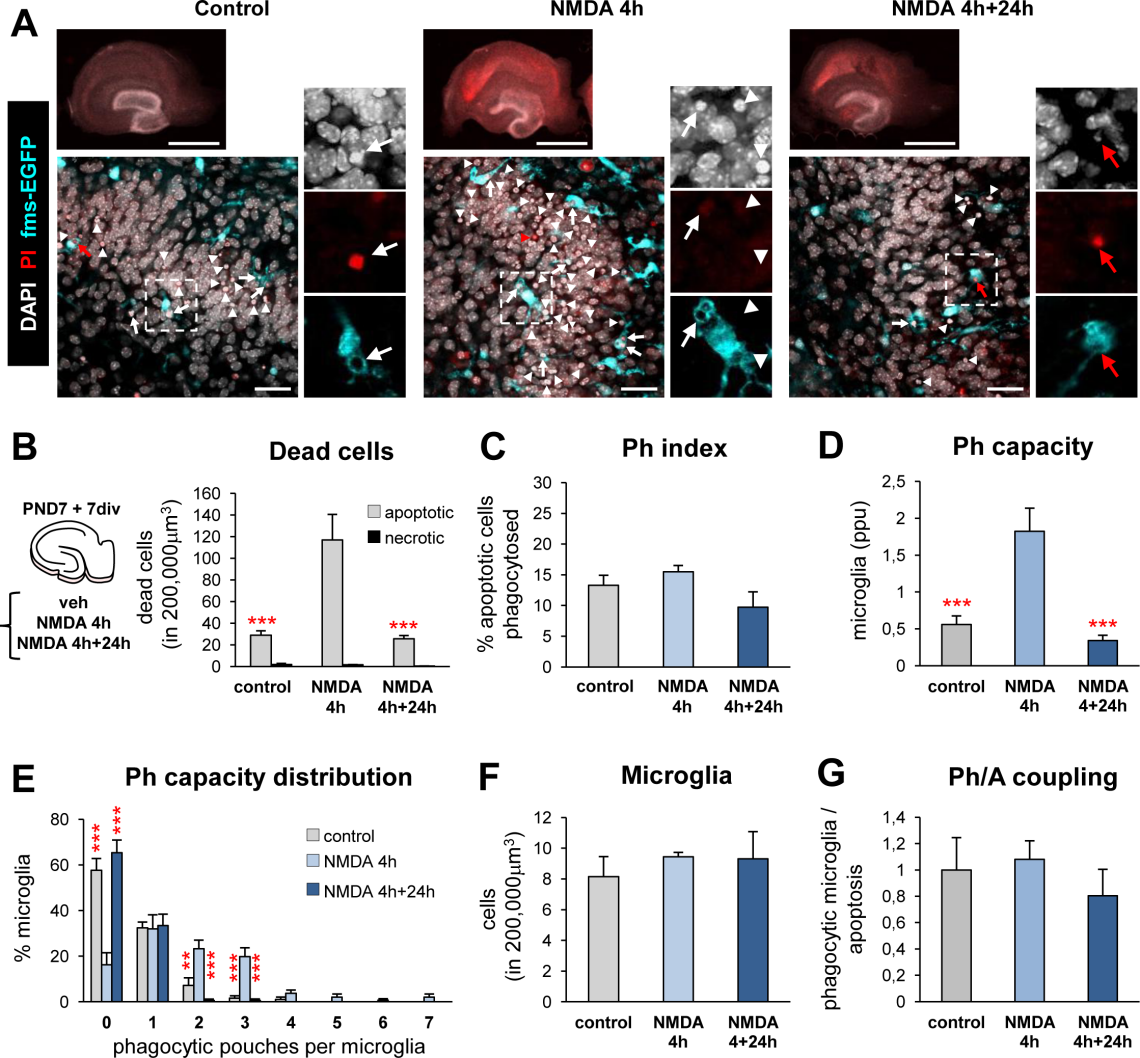
Microglial phagocytic response during in vitro excitotoxic challenge. (**A**) Representative epifluorescence (upper panels) and confocal (middle panel) images of the DG in hippocampal organotypic cultures treated with NMDA (50 μM) for 4 h, or fresh media for another 24 h. Normal or apoptotic (pyknotic/karyorrhectic) nuclear morphology was visualized with DAPI (white), microglia by the transgenic expression of fms-EGFP (cyan) and membrane permeability (characteristic of necrotic cells) by PI (red). High magnification inserts show a phagocytosed secondary apoptotic cell (pyknotic, PI^+^; left panel, arrow); primary apoptotic cells (pyknotic, PI^-^), phagocytosed or not (arrow and arrow heads, respectively, central panel); and phagocytosed necrotic (nonpyknotic, PI^+^; red arrow, right panel). Scale bars, upper panel = 1 mm, lower panel = 30 μm. (**B**) Number of dead apoptotic (primary and secondary together) and necrotic cells in 200.000 μm^3^ of the DG in organotypic slices treated with NMDA. (**C**) Ph index in organotypic slices (% of apoptotic cells phagocytosed) treated with NMDA. The Ph index in vivo of animals at PND7 (the age at which the organotypic slices were cultured) and PND14 (equivalent to the age of the cultures after 1 wk in vitro) is shown in **S1A and S1B Fig** (**D**) Weighted Ph capacity of microglia (in parts per unit, ppu). (**E**) Histogram showing the Ph capacity of microglia (in % of cells). (**F**) Number of microglial cells. (**G**) Ph/A coupling (in fold-change) in organotypic slices treated with NMDA. Bars represent mean ± SEM. * indicates *p* < 0.05, ** indicates *p* < 0.01, and *** indicates *p* < 0.001 by Holm-Sidak posthoc test (after one-way ANOVA was significant at *p* < 0.05). Only significant effects are shown. Underlying data is shown in **S1 Data**.

To analyze the microglial response to an apoptotic challenge, we used an in vitro model of excitotoxicity without seizures induced in postnatal (PND) organotypic hippocampal cultures by treatment with the glutamate agonist NMDA (N-methyl-D-aspartate, 50 μM) (**Fig 1**). Apoptosis was determined by aberrant nuclear morphology visualized with the DNA dye DAPI (pyknosis/karyorrhexis), necrosis by retention of propidium iodide (PI), and microglial phagocytosis by the appearance of phagocytic pouches in fms-EGFP mice, in which all microglia express the fluorescent reporter [10] (**Fig 1A**). The number of apoptotic, but not of necrotic, cells increased significantly after 4 h of NMDA treatment compared to nontreated controls and returned to basal levels 24 h later (**Fig 1B**). The basal phagocytic (Ph) index, i.e., the proportion of apoptotic cells completely engulfed by microglia, was 15 ± 2% in organotypic slices (**Fig 1C**) lower than in the PND (60%–70%; **S1A and S1B Fig**), and adult (90%–100%; [9]) dentate gyrus (DG), evidencing changes induced by culturing the tissue. When challenged with NMDA, microglia responded to the increased number of apoptotic cells by rising their Ph capacity, i.e., the proportion of microglia with one or more phagocytic pouches, each containing one apoptotic cell [9], and there were more phagocytic microglia overall, some of them with up to seven pouches (**Fig 1D and 1E**), while the number of microglia remained unchanged (**Fig 1F**). Thus, the increase of net phagocytosis (number of microglia multiplied by their phagocytic capacity) matched the increase in apoptosis, as determined by the phagocytosis/apoptosis coupling ratio (Ph/A coupling). The Ph/A coupling was similar between control and NMDA-treated slices (**Fig 1G**).

**Fig 2:**
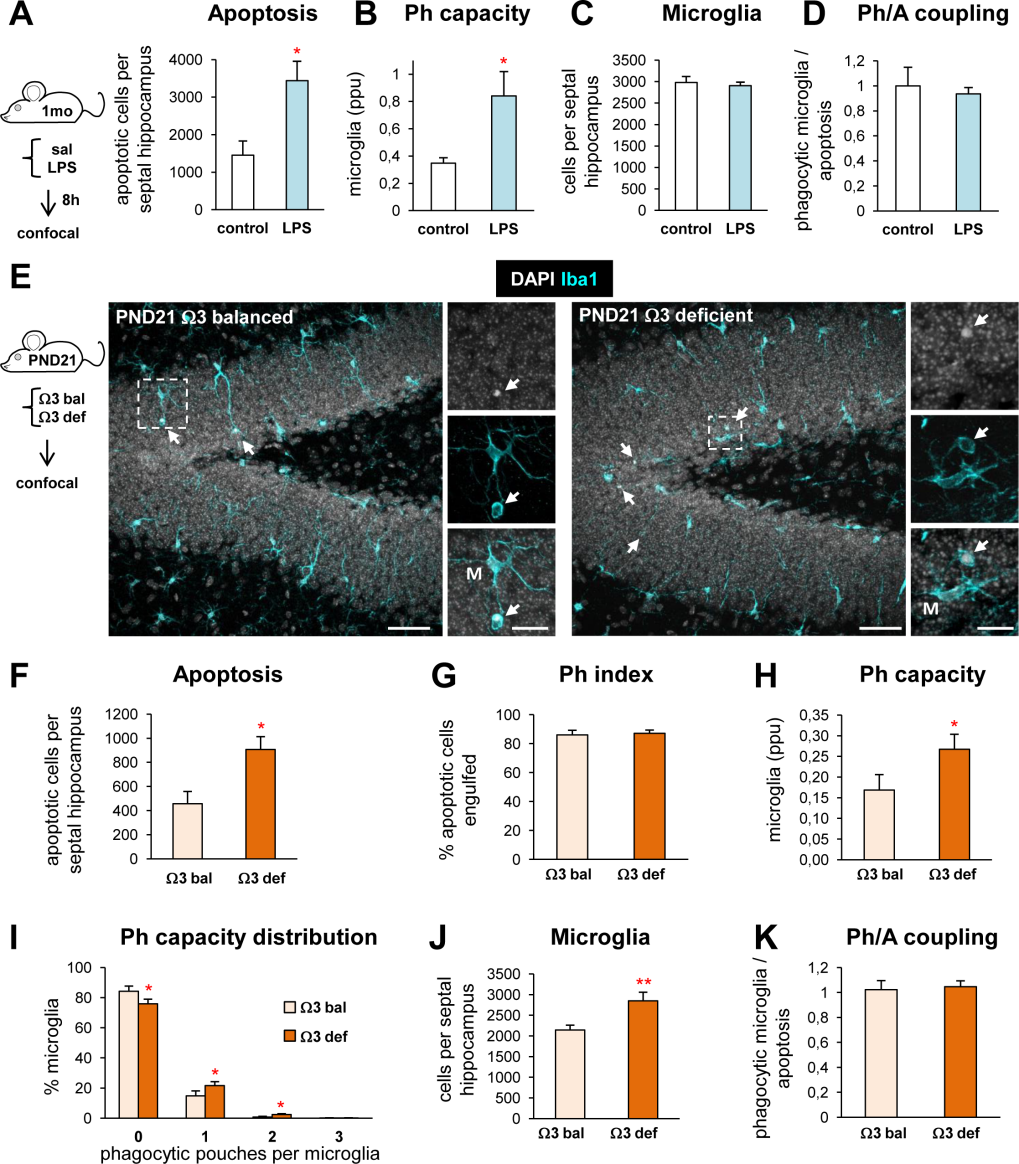
Microglial phagocytic response during in vivo acute and chronic inflammatory challenge. (**A**) Experimental design and apoptosis in the DG of c57BL/6 fms-EGFP 1-mo mice injected systemically with LPS (1mg/kg; *n* = 5) or vehicle (saline; *n* = 4) 8 h prior to sacrifice. Apoptotic cells were identified by pyknosis/karryorhexis. **Fig 2A** was generated from data that was originally published as part of [9]. (**B**) Weighted Ph capacity of microglia (in parts per unit, ppu) in control and LPS mice. (**C**) Number of microglial cells in control and LPS mice. (**D**) Ph/A coupling in the 1-mo mouse hippocampus (in fold change) during acute inflammatory challenge. (**E**) Experimental design and representative confocal z-stacks of the DG of PND21 Swiss mice fed during gestation and lactation with a diet balanced (Ω3 bal; *n* = 7) or deficient (Ω3 def; *n* = 7) in the omega 3 polyunsaturated fatty acid, a diet that induces chronic inflammation in the hippocampus. Microglia were labeled with Iba1 (cyan) and apoptotic nuclei were detected by pyknosis/karyorrhexis (white, DAPI). Arrows point to apoptotic cells engulfed by microglia (M). Scale bars = 50 μm; z = 22.5μm. (**F**) Number of apoptotic (pyknotic/karyorrhectic) cells in mice fed with Ω3 balanced and deficient diets. (**G**) Ph index in the PND21 hippocampus (in % of apoptotic cells) in mice fed with Ω3 balanced and deficient diets. (**H**) Weighted Ph capacity of microglia (in ppu) in PND21 mice. (**I**) Histogram showing the Ph capacity distribution of microglia (in % of cells) in PND21 mice. (**J**) Total number of microglial cells (Iba1^+^) in PND21 mice. (**K**) Ph/A coupling in PND21 mice. Bars represent mean ± SEM. * indicates *p* < 0.05 and ** indicates *p* < 0.01 by one-tail Student´s *t* test. Underlying data is shown in **S1 Data**.

Remarkably, we observed the same type of coupled microglial phagocytic response in vivo after acute and chronic inflammatory challenge (**Fig 2**). After acute inflammatory challenge induced by bacterial lipopolysaccharides (LPS; 1 mg/kg i.p., 8 h, 1-mo-old mice), apoptosis increased in the DG (**Fig 2A**) [9], and hippocampal microglia responded by proportionally raising their phagocytic capacity (**Fig 2B**) without increasing their number (**Fig 2C**). As a result, the Ph/A coupling ratio (**Fig 2D**) and the Ph index remained unchanged (95.9 ± 2.2% versus 93.3 ± 1.4% of apoptotic cells engulfed in control versus LPS, respectively [9]). We observed a similar response in a model of chronic inflammatory challenge induced in young (PND21) mice fed during embryonic and PND development with a diet deficient in anti-inflammatory omega 3 polyunsaturated fatty acids [11]. In omega 3-deficient mice there was an increase in apoptosis in the DG (**Fig 2E and 2F**) but the Ph index remained unaltered compared to mice fed during gestation and lactation with an omega 3 balanced diet (**Fig 2G**). The increase in apoptosis was matched by a partial increase in the Ph capacity and an increase in the number of microglial cells (**Fig 2H–2J**), ultimately resulting in the maintenance of the Ph/A coupling (**Fig 2K**). Thus, both after excitotoxic challenge in vitro and acute and chronic inflammatory challenge in vivo, microglial phagocytosis remained tightly coupled to apoptosis.

### Acute Impairment of Microglial Phagocytosis Following Seizures In Vivo

**Fig 3:**
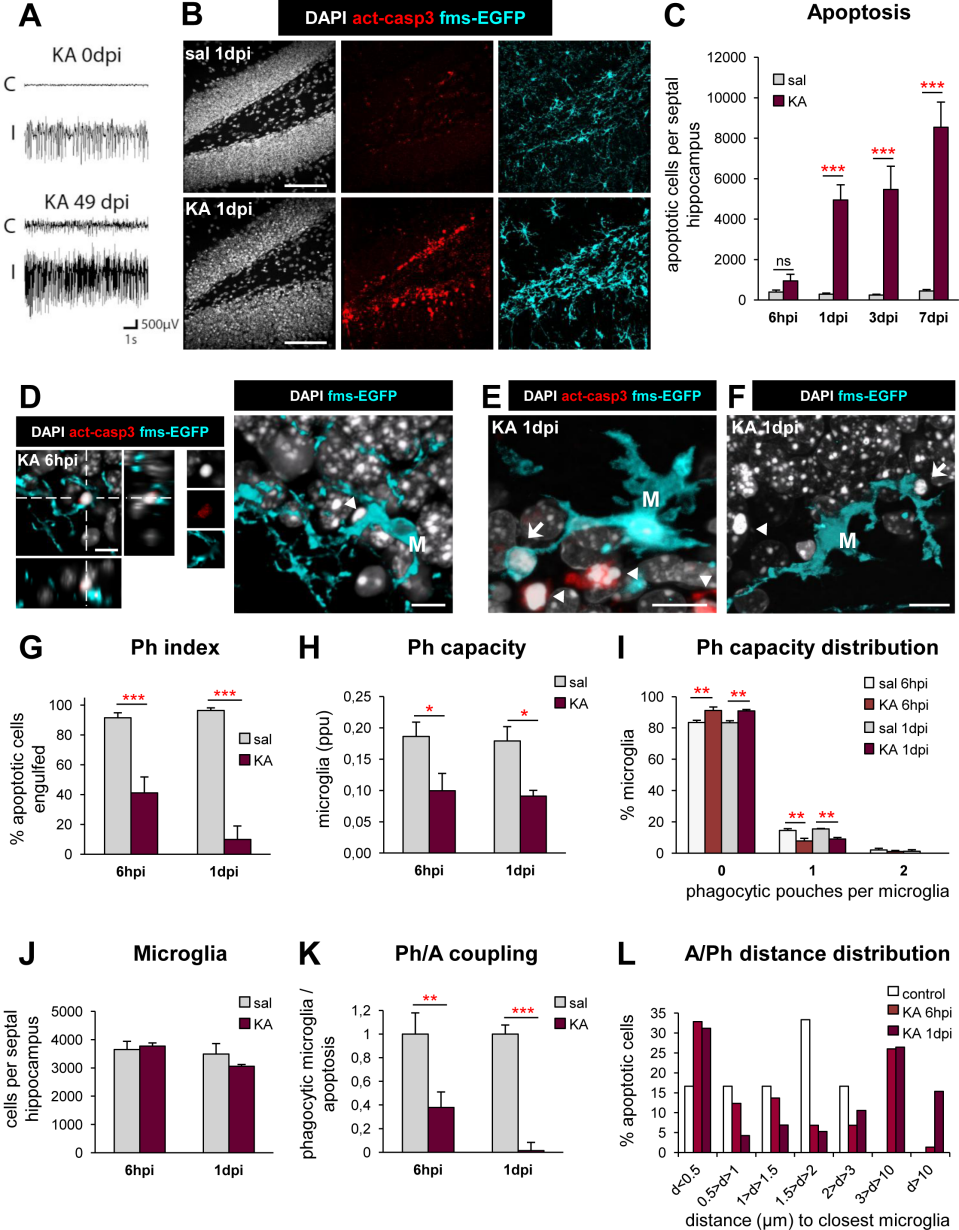
Microglial phagocytosis is impaired early (1 dpi) due to MTLE seizures in vivo. (**A**) Hippocampal electroencephalographic recordings of mice injected in the ipsilateral side (I) with KA (50 nL, 20 mM) during status epilepticus (0 dpi) and during a spontaneous seizure occurring in the chronic phase of MTLE (49 dpi). The contralateral hippocampus (C) is shown for comparison purposes. (**B**) Representative confocal z-stacks of saline and KA (1 dpi) hippocampi labeled with DAPI (nuclear morphology, white), activated caspase 3 (act-casp3^+^, red, for apoptotic cells), and fms-EGFP (cyan, microglia). (**C**) Number of apoptotic cells (pyknotic/karyorrhectic and act-casp3^+^) in the septal DG (*n* = 3−9 per time point and treatment). The volume of the septal DG is shown in **S3B Fig**. (**D**) Representative confocal image of a nonphagocytosed apoptotic (pyknotic and act-casp3^+^, arrowhead) cell in the SGZ (orthogonal projection, left; and 3-D-rendered image, right). M, microglial cell body. (**E**) Representative 3-D-rendered confocal z-stack of apoptotic (pyknotic and act-casp3^+^) cells, phagocytosed (arrow) or not (arrowheads) in the septal DG of mice treated with KA at 1 dpi. M, microglial cell body. (**F**) Representative 3-D-rendered confocal z-stack of an apoptotic (pyknotic), nonphagocytosed cells (arrowhead) in the DG of mice treated with KA at 1 dpi. The arrow points to a semiengulfed apoptotic cell. M, microglial cell body. (**G**) Ph index in the septal DG (in % of apoptotic cells) after KA. Phagocytosis by astrocytes and neuroblasts is shown in **S3C and S3E Fig**. (**H**) Weighted Ph capacity of DG microglia (in ppu). (**I**) Histogram showing the Ph capacity distribution of microglia (in % of cells) in the DG. (**J**) Total number of microglial cells (fms-EGFP^+^) in the septal DG. Microglial density is shown in **S3A Fig**. (**K**) Ph/A coupling (in fold change) in the septal DG. (**L**) Histogram showing the distribution of the distance (in μm) of apoptotic cells (in %) to microglial processes. The average distance of apoptotic cells to microglia is shown in **S3F Fig**. Bars represent mean ± SEM except in L, where they indicate the sum of cells in each distance slot. * indicates *p* < 0.05, ** indicates *p* < 0.01, and *** indicates *p* < 0.001 by Holm-Sidak posthoc test after two-way ANOVA (H–K) or one-way ANOVA (C, G, where a significant interaction time x treatment was found) were significant at *p* < 0.05. Scale bars = 50 μm (B), 10 μm (D–F). z = 25 μm (B), 13.9 μm (D), 14.1 μm (E), 8.4 μm (F). Underlying data is shown in **S1 Data**.

The above results suggest that microglia have a substantial reservoir for phagocytosis, as they could reach their maximum Ph capacity by recruiting up to 100% microglia to be phagocytic, by inducing each microglia to phagocytose more apoptotic cells, and/or by increasing the total number of microglia. To test this potential, we challenged microglia in an in vivo model of MTLE, in which seizures concur with excitotoxicity and inflammation (**Fig 3**). Intrahippocampal administration of kainic acid (KA) induced an episode of prolonged continuous seizure activity (status epilepticus) that lasted 4–6 h. All animals reached level 3–4 class seizures according to the Racine scale [12], and the development of spontaneous recurrent seizures was monitored for up to 7 wk (**Fig 3A**) [13]. Apoptosis was consistently induced in the medial hippocampus (spanning from −1 mm to −2.5 mm in the anteroposterior (AP) axis, from Bregma), and thus quantifications were restricted to that area. We quantified the absolute number of apoptotic cells (determined by pyknosis/karyorrhexis and/or activated caspase 3 staining) along a time course from 6 h postinjection (hpi) to 7 dpi (d postinjection) (**Fig 3B and 3C**). In the DG, the number of apoptotic cells, mostly located in the subgranular zone (SGZ) where neural stem cells reside, significantly increased starting from 1 dpi and up to 7 dpi relative to controls. Unexpectedly, we found limited evidence of phagocytosis (**Fig 3D–3F**), and the Ph index significantly dropped at 6 hpi and 1 dpi (**Fig 3G**). In addition, we also found a consistently low Ph index in the CA1 and CA3 regions of the hippocampus, as well as in the adjacent somatosensory cortex (**S2A–S2C Fig**). In these regions, apoptosis was undetectable in control conditions, and thus the basal Ph index could not be estimated.

We further analyzed the characteristics of this phagocytosis impairment in the DG. We found a decreased Ph capacity at both 6 hpi and 1 dpi compared to controls, due to a smaller proportion of microglia with phagocytic pouches (**Fig 3H and 3I**). While no significant changes in total microglial numbers were found (**Fig 3J**), there was a significant decrease in the microglial density at 1 dpi (**S3A Fig**), which can be attributed to the increase in the DG volume (due to granule cell dispersion; **S3B Fig**), typical of both human and mouse MTLE. As a result of the decreased Ph capacity induced by KA, the Ph/A coupling ratio dramatically decreased (**Fig 3K**). In summary, instead of an increase in phagocytosis after the KA challenge matching the increase in apoptosis, as expected from our experiments with excitotoxicity and inflammation, the microglial phagocytic response was reduced as early as 6 hpi following the KA challenge.

We then argued that microglial phagocytosis impairment could be compensated by the recruitment of other resident cells endowed with phagocytic potential, such as astrocytes [14] or neuroblasts [15], which do not normally phagocytose hippocampal apoptotic cells in resting conditions [9]. To test this hypothesis, we used transgenic mice in which the expression of fluorescent reporters is controlled by cell-type specific promoters, i.e., human glial fibrillary acidic protein (hGFAP) for astrocytes and proopiomelanocortin (POMC), which in the hippocampus is only expressed in neuroblasts [16]. We found that both cell types were engaged in phagocytosis after 1 dpi of KA, but nonetheless, they only engulfed a small proportion of the apoptotic cells compared to microglia (**S3C and S3E Fig**). Therefore, even at 1 dpi after KA, microglia remained the most determinant phagocyte in the hippocampus.

### Subacute Partial Recovery of Microglial Phagocytosis Coincides with Multinuclearity and Phagoptosis, and Is Unrelated to Monocyte Invasion

**Fig 4:**
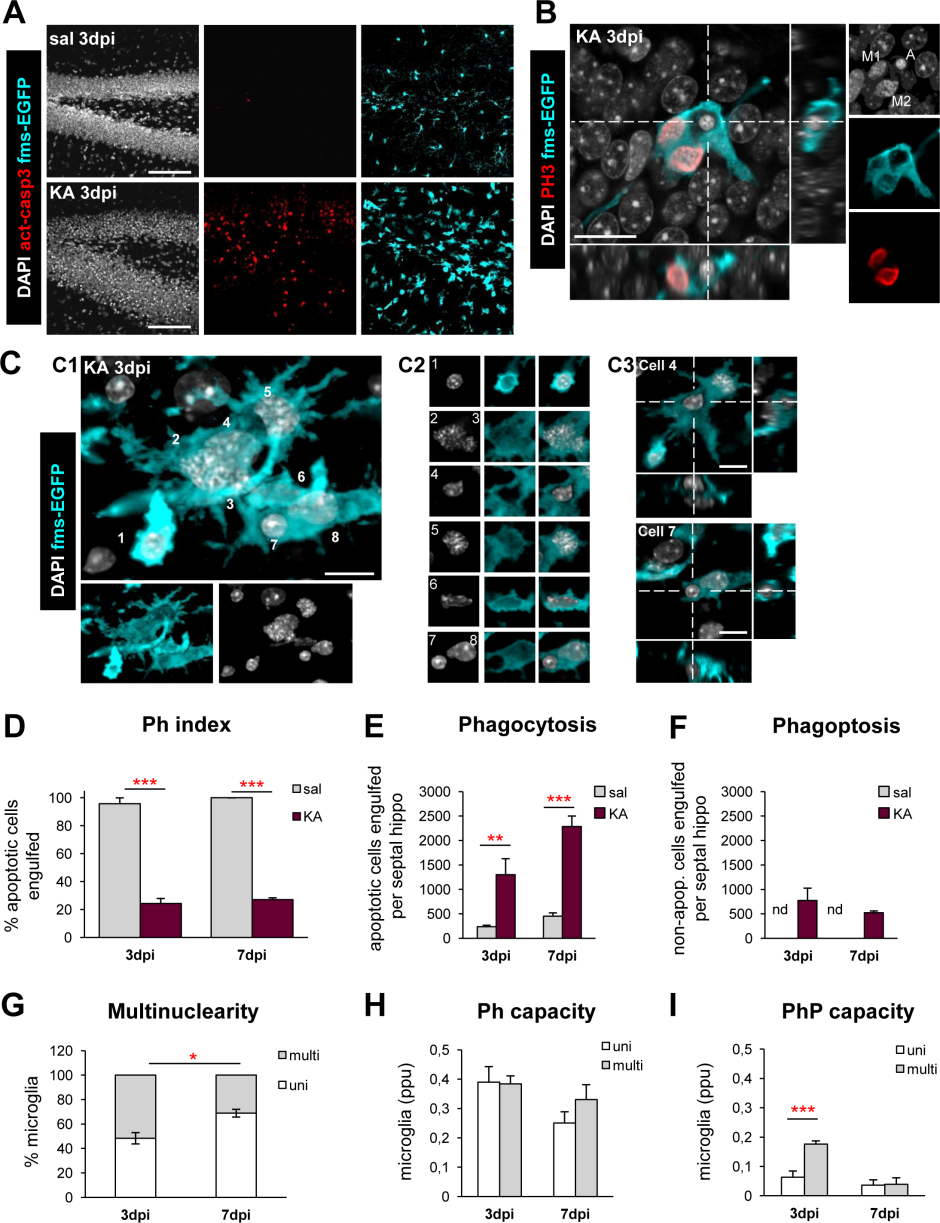
Partial recovery of microglial phagocytic efficiency at 3 and 7 dpi is accompanied by multinuclearity and phagoptosis. (**A**) Representative confocal z-stacks of saline and KA (3 dpi) hippocampi labeled with DAPI (nuclear morphology, white), activated caspase 3 (act-casp3^+^, red, for apoptotic cells), and fms-EGFP (cyan, microglia). At 3 dpi, KA led to an accumulation of apoptotic cells throughout the DG. (**B**) Orthogonal projection of a confocal z-stack from the hippocampus of a KA-treated mouse (3 dpi) showing a binucleated microglia cell (fms-EGFP^+^, cyan) undergoing division (phosphohistone 3, PH3, red) and phagocytosing an apoptotic cell (pyknotic by DAPI, white; A). Note the condensed chromosomes in the microglial nuclei, typical of mitosis (M1, M2). (**C**) Representative confocal z-stack of a large multinucleated phagoptotic microglia (fms-EGFP^+^, cyan) from the hippocampus of a KA mouse (3 dpi). Another example is shown in **S4A Fig**. (**C1**). 3-D-rendered image showing the continuum of EGFP through the microglial cytoplasm and within their nuclei. Up to eight nuclei were contained. (**C2**) Panel showing each nucleus individually. Nuclei 2, 3, and 5 showed condensed chromosomes, characteristic of an ongoing mitosis. Nuclei 4 and 7 were small but not pyknotic; they did not contain EGFP and thus were not microglial but rather surrounded by pouches of microglial cytoplasm. (**C3**) Orthogonal projections of nuclei 4 and 7 showing their complete engulfment by microglial processes (phagoptosis). (**D**) Ph index (in % of cells) in the septal hippocampus at 3 and 7 dpi (*n* = 3–7 per group). (**E**) Total number of phagocytosed apoptotic cells in the septal hippocampus after treatment with KA (3 and 7 dpi). (**F**) Total number of phagoptosed nonapoptotic cells in the septal hippocampus after treatment with KA (3 and 7 dpi). Nonphagoptosed cells were found in control animals. nd, not detected. (**G**) Proportion (in % of cells) between uni- and multinucleated microglia in the hippocampus of KA-treated mice at 3 and 7 dpi. (**H**) Weighted Ph capacity in the septal hippocampus after treatment with KA (3 and 7 dpi). (**I**) Weighted PhP (phagoptosis) capacity in the septal hippocampus after treatment with KA (3 and 7 dpi). Bars show mean ± SEM. * indicates *p* < 0.05, ** indicates *p* < 0.01, and *** indicates *p* < 0.001 by Student´s *t* test (G) or by Holm-Sidak posthoc test after two-way ANOVA (D, E) or one-way ANOVA (I) were significant at *p* < 0.05. Only significant effects are shown. Scale bars = 50 μm (A), 10 μm (B, C); z = 25 μm (A), 10.5 μm (C). Underlying data is shown in **S1 Data**.

To determine the extent of microglial impairment over time, we analyzed apoptosis and phagocytosis over a time course of 3 to 7 dpi after the KA challenge (**Fig 4**). The number of apoptotic cells continued to increase over this period (**Fig 3C**), but now the cells had a wider distribution throughout the DG (**Fig 4A**). In contrast to 1 dpi, phagocytosis was evident in KA mice at 3 dpi and 7 dpi (**Fig 4B and 4C**), although the Ph index remained significantly lower than in control mice (**Fig 4D**), indicating a continued impairment. Nonetheless, some recovery of phagocytosis occurred, because the net phagocytosis increased significantly (**Fig 4E**). In addition to the phagocytosis of apoptotic cells, we found some cases of phagoptosis, i.e., engulfment of nonapoptotic cells. This phenomenon occurred at lower frequency than the phagocytosis of apoptotic cells (**Fig 4C and 4F**). Phagoptosis, which is a process triggered by inflammation [17], was undetectable in control mice (**Fig 4F**), LPS-treated mice, or omega 3-deficient mice at the time points tested.

Furthermore, microglia developed a hypertrophic, seemingly ameboid morphology, which was accompanied by an incomplete mitosis (nucleokinesis without cytokinesis), as determined by staining with the cell cycle marker phosphohistone 3 (PH3) (**Fig 4B**). While in saline mice all microglia had a single nucleus, KA treatment significantly increased multinuclearity (**Fig 4G**). In KA mice, uni- and multinucleated cells performed phagocytosis similarly (**Fig 4H**). Phagoptosis was executed mostly by multinucleated cells at 3 dpi, and similarly by uni and multinucleated microglia at 7 dpi (**Fig 4I**). Because multinucleated microglia frequently overlapped spatially, it was not possible to estimate their numbers precisely, and the Ph/A coupling ratio was not calculated. To further characterize microglia in this context, we analyzed their proliferation with the cell cycle marker Ki67 [18] and their expression of classic activation markers such as CD11b (integrin αM) and CD68 (macrosialin) [19]. We found that DG microglia began to proliferate at 3 dpi (**S5 Fig**), coincident with the proliferation of other cell types and a large activation of the proliferative molecular program induced by KA [13]. We also found an increased expression of CD11b and CD68 along the time course that reached a maximum at 7 dpi and was not restricted to the DG as it spread over the whole hippocampus (**Fig 7 and S6 Fig**). Nonetheless, we found no apparent correlation between the expression of these markers and the phagocytosis impairment. Thus, at 3 and 7 dpi, there was a partial recovery of microglial phagocytosis, coincidental with multinuclearity and phagoptosis.

**Fig 5:**
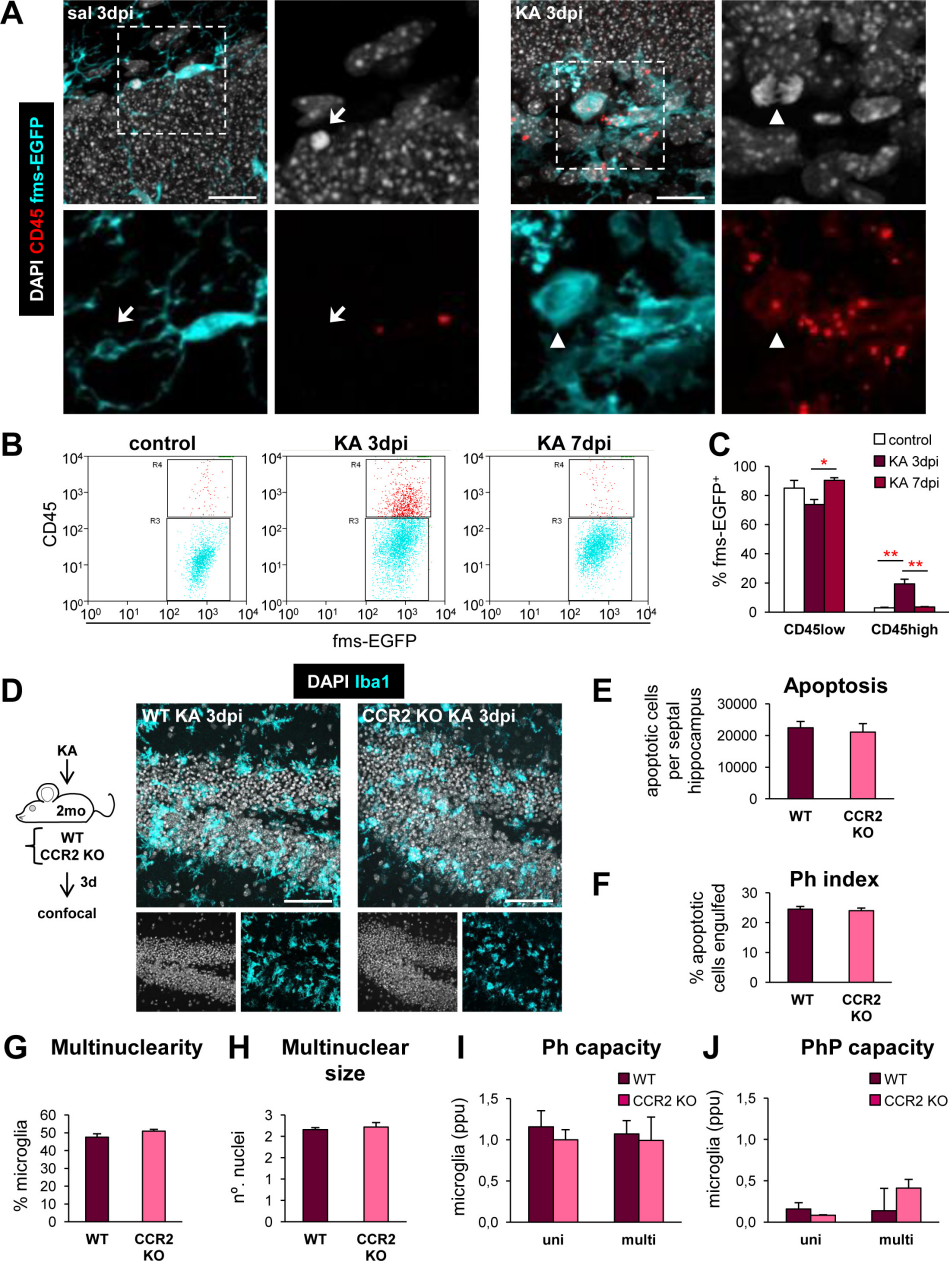
Microglial phagocytosis impairment is unrelated to monocytes. (**A**) CD45 staining in saline- and KA-injected mice at 3 dpi. Cell nuclei are shown in white (DAPI), microglia in cyan (fms-EGFP), and CD45 in red. In control mice, the expression of CD45 was dim, showing diffuse cytoplasmic inclusions within microglia. A CD45^+^ cell is shown engulfing an apoptotic cell (arrow, enlarged). In KA mice, CD45 had a higher and more widespread expression in all microglial cells, including a dividing cell (arrowhead, enlarged). A clear distinction between CD45^high^ and CD45^low^ cells was not evident. (**B**) Flow cytometry analysis of the expression of CD45 in fms-EGFP^+^ hippocampal cells from control and KA-treated mice. Gates for CD45^low^ (cyan) and CD45^high^ (red) were defined based on the distribution of the fms-EGFP^+^ cells in control (not injected) mice. 3 dpi after the KA injection, more cells were found in the CD45^high^ gate, although the fms-EGFP^+^ cells were in fact distributed along a continuum of CD45 expression, all of them with higher expression than control mice. At 7 dpi, the expression of CD45 returned to basal levels. The gating strategy is shown in **S4B Fig**. (**C**) Percentage of fms-EGFP^+^ cells that expressed low or high levels of CD45 in control or KA-treated mice determined by flow cytometry (*n* = 4 per group). (**D**) Experimental design and representative confocal z-stacks of the hippocampus of CCR2^-/-^ (CCR2 KO) mice and control WTs (C57BL/6) injected with KA (3 dpi). No obvious differences in the status epilepticus, neuronal damage, microglial morphology, nor in the DG volume (**S4C Fig**), or neutrophil infiltration were found (**S4D and S4E Fig**). (**E**) Number of apoptotic (pyknotic/karyorrhectic) in the septal DG in WT and CCR2 KO mice 3 dpi after KA (*n* = 4 per group). (**F**) Ph index in the septal DG (in % of apoptotic cells) in WT and CCR2^-/-^ mice 3 dpi after KA. (**G**) Multinuclearity in WT and CCR2^-/-^ mice. (**H**) Size of multinucleated cells in WT and CCR2^-/-^ mice. (**I**) Weighted Ph capacity in WT and CCR2^-/-^ mice. Note that the Ph capacity is higher than in our previous time course (**Fig 4H**), reflecting an increased number of apoptotic cells in this experiment compared to the previous one, possibly because it was performed in different animal facilities. (**J**) Weighted PhP (phagoptosis) capacity in the septal DG in WT and CCR2^-/-^ mice. Data are shown as mean ± SEM. * indicates *p* < 0.05, ** indicates *p* < 0.01, and *** indicates *p* < 0.001 by Holm-Sidak posthoc test, after one-way ANOVA was significant at *p* < 0.05; only significant interactions are shown. Scale bars = 20 μm (A), 50 μm (D); z = 14.7 μm (A), 12.6 μm (D). Underlying data is shown in **S1 Data**.

The abnormal microglial phenotype we observed in KA-treated mice might be related to the invasion of bone marrow-derived monocytes. To test this possibility, we analyzed the expression of CD45, a lymphocyte antigen with higher expression in circulating cells compared to resident microglia [10]. CD45 expression was undetectable in saline-injected mice but was evident in all fms-EGFP-expressing cells at 3 dpi after KA by immunofluorescence (**Fig 5A**). Flow cytometry analysis showed a transiently increased CD45 expression in the fms-EGFP population at 3 dpi that returned to basal levels by 7 dpi (**Fig 5B and 5C**). This finding could be interpreted as resulting from a transient overexpression of CD45 in resident microglia, or from an invasion of CD45^high^ monocytes that either died or down-regulated the antigen later on. To further test the role of invading monocytes, we injected KA in mice lacking CCR2, the main receptor for the chemokine MCP1 (monocyte chemoattractant protein), which is involved in the recruitment of circulating monocytes into the damaged central nervous system [20]. CCR2^-/-^ mice have a greatly decreased population of circulating monocytes and recruitment into the brain parenchyma [21]. CCR2^-/-^ mice reached similar levels to WT mice in the Racine scale (3–4) after KA, and the amount of apoptosis at 3 dpi was similar in wild type and CCR2^-/-^ mice (**Fig 5D and 5E**). As noted above, the number of microglial cells in these mice could not be accurately assessed because of their ameboid, multinucleated morphology, but no obvious differences were observed. Importantly, both genotypes showed a similar phagocytosis impairment determined by the Ph index (**Fig 5F**), the percentage of multinuclear microglia, and the average number of nuclei per cell (**Fig 5G and 5H**), without any significant changes in DG volume (**S4C Fig**). Likewise, phagocytosis and phagoptosis by uni- and multinucleated microglia were similar in both genotypes (**Fig 5I and 5J**). We also tested potential compensatory mechanisms such as neutrophil invasion. However, the presence of circulating neutrophils labeled with myeloperoxidase [22] was very low in both genotypes (3.8 ± 2.0 versus 3.0 ± 2.1 cells per hippocampus in WT versus CCR2 KO mice, respectively; **S4D and S4E Fig**). Overall, these data indicate that invading peripheral immune cells do not contribute significantly to the microglial population in the early stages of experimental MTLE.

### Chronic Impairment of Microglial Phagocytosis in Mouse and Human MTLE

**Fig 6:**
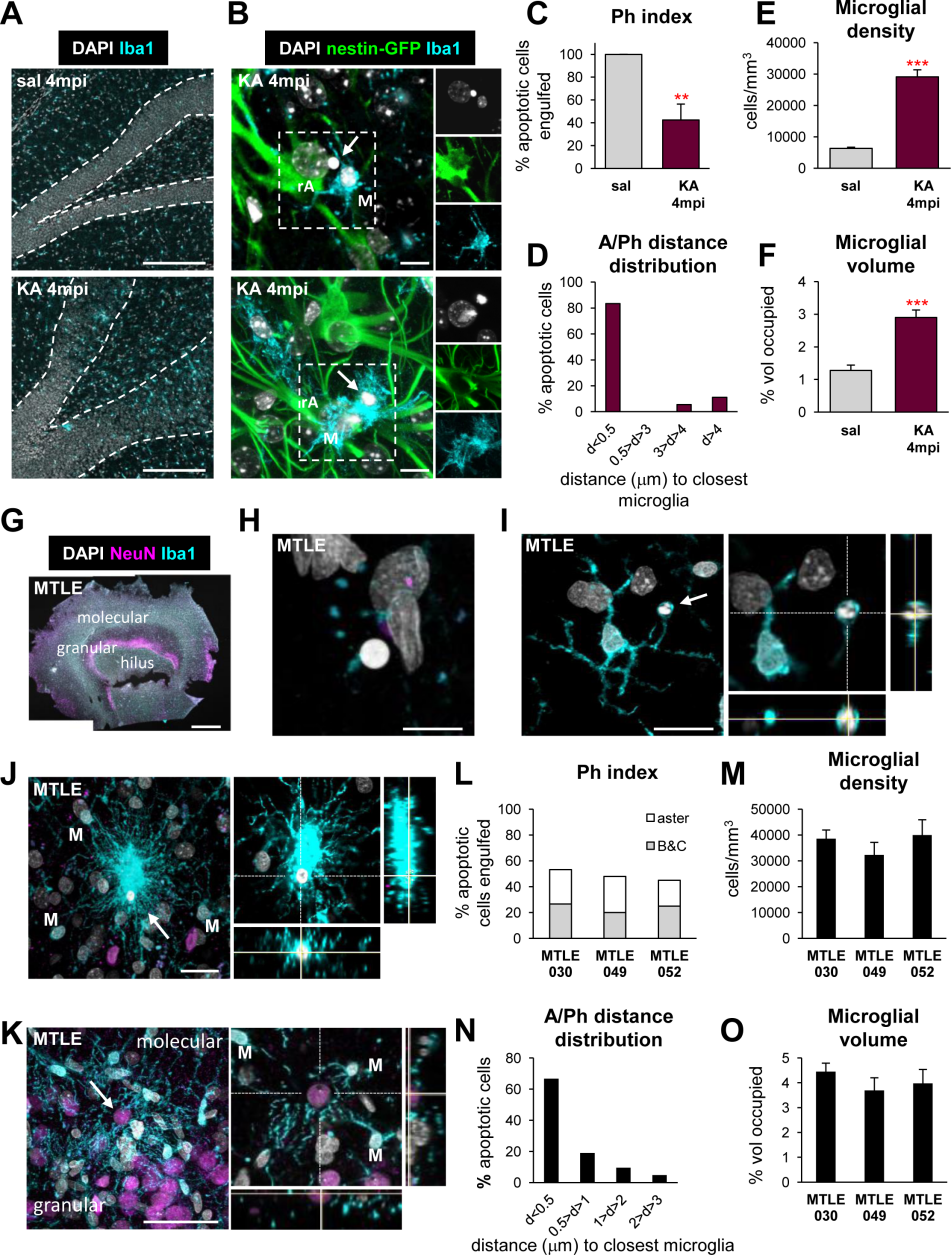
Long-term impairment of microglial phagocytosis in mouse and human MTLE. (**A**) Representative confocal images of the DG of saline- and KA-injected mice at 4 mpi showing the nuclei (with DAPI, in white) and microglia (Iba1^+^, in cyan). Note the gross dispersion of the DG in KA injected mice (**S4F Fig**). The number of apoptotic cells in control and KA-treated mice at 4 mpi is shown in **S4G Fig**. (**B**) Upper panel: representative confocal z-stack of an apoptotic cell (pyknotic, with DAPI, in white; arrowhead) located nearby a hypertrophic reactive astrocyte (rA; visualized with nestin-GFP^+^, in green) and a microglial cell (M; Iba1^+^, in cyan) at 4 mpi after KA. Lower panel: representative confocal z-stack of an apoptotic cell phagocytosed by microglia at 4 mpi after KA. A representative image of phagocytosis by a reactive astrocyte at 4 mpi after KA is shown in **S4H Fig**. (**C**) Ph index in the DG (% of apoptotic cells engulfed). (**D**) Histogram showing the distribution of the distance (in μm) of apoptotic cells to microglia at 4 mpi after KA (in %). (**E**) Density of microglial cells (in cells/mm^3^). (**F**) Microglial volume (in % of volume of DG occupied). (**G**) Representative confocal tiled image of a slice of the human hippocampus from an MTLE patient showing cell nuclei (with DAPI, white), neuronal nuclei (NeuN^+^, magenta), and microglia (Iba1^+^, cyan). (**H**) Representative confocal image of a nonphagocytosed apoptotic cell (pyknotic, with DAPI) adjacent to a microglial process (Iba1^+^) in the hippocampus of an MTLE patient. (**I**) Representative confocal image of phagocytosis by a ball-and-chain mechanism in the hippocampus from an individual with MTLE. The apoptotic cell (pyknotic, with DAPI in white; arrow) was engulfed by a terminal branch of a nearby microglia (Iba1^+^, cyan). The right panel shows an orthogonal projection of the same cell, where the 3-D engulfment is evident. (**J**) Representative confocal z-stack of phagocytosis by an aster mechanism in the hippocampus from an individual with MTLE. The apoptotic cell (pyknotic, with DAPI in white; arrow) was engulfed by a mesh of processes from many surrounding microglia (Iba1^+^, cyan; M). The right panel shows an orthogonal projection of the same cell. (**K**) Representative confocal z-stack of a granule neuron in the DG (NeuN^+^, magenta; arrow) targeted by the processes of several surrounding microglia (Iba1^+^). Nuclei are shown in white (DAPI). The right panel shows an orthogonal projection of the same neuron directly targeted the processes of up to three microglia (M). Another example is shown in **S8A Fig** and further data in **S1 Table**. (**L**) Ph index in the human DG (% of apoptotic cells engulfed). (**M**) Density of microglial cells (in cells/mm^3^) in the DG of three hippocampal samples from human MTLE patients. (**N**) Histogram showing the distribution of the distance of apoptotic cells (in %) to Iba1^+^ microglial processes in the DG of MTLE patients (*n* = 21 cells from 3 patients). (**O**) Microglial volume (in % of volume of DG occupied) in the three hippocampal samples from individuals with MTLE. Bars represent mean ± SEM (C, E, F), the individual values of all the pooled cells for each patient (L), the average values for measures in different z-stacks for each patient (M, O), or the sum of cells in each distance slot (D, N). ** represents *p* < 0.01 by Student´s *t* test (C, E, F). Scale bars = 50μm (A, K), 10 μm (B, H, I), 1 mm (G), 20 μm (J). *z* = 25 μm (A), 6.6 μm (B, upper panel), 12.7 μm (B, lower panel), 2.8 μm (H), 2.6 μm (I), 5.2 μm (J), 12 μm (K). Underlying data is shown in **S1 Data**.

We next sought to confirm that microglial phagocytosis is also impaired in human MTLE. Because in humans MTLE is a chronic disease, we first analyzed microglial phagocytosis in KA-treated mice 4 mo postinjection (mpi), when animals have chronic epilepsy [13]. At this time, hippocampal sclerosis characterized by granule cell dispersion and the appearance of reactive, hypertrophic astrocytes [23] was evident in KA mice (**Fig 6A and S4F Fig**). At 4 mpi after KA, apoptosis had returned to basal levels (**S4G Fig**), but a substantial number of apoptotic cells were not phagocytosed whether by microglia (Ph index = 43 ± 14%) (**Fig 6B and 6C**) or by reactive astrocytes (Ph index = 5 ± 3%) (**S4H Fig**). Furthermore, over 80% of the nonphagocytosed cells analyzed were less than 0.5 μm apart of a microglial process (**Fig 6D**). To test whether this impaired microglial phagocytosis was related to a decreased surveillance, we analyzed the density of microglial cells and the percentage of the parenchyma occupied by microglial processes (microglial volume), and found that both parameters increased significantly in KA mice at 4 mpi compared to controls (**Fig 6E and 6F**). Thus, impairment of microglial phagocytosis was long-lasting in our chronic MTLE experimental model in spite of their increased density and volume occupied.

Next, we tested microglial phagocytosis in hippocampal tissue resected from drug-resistant MTLE patients, in which the hippocampal formation was removed to control the seizures. The tissue had minimal postoperative delay before it was fixed (under 40 min), which preserved antigenicity and prevented further neuronal damage (**Fig 6G**). In the three MTLE patients analyzed (two males, one female, aged 38–56), apoptosis was low (typically 10–20 apoptotic cells per slice). As in the mouse MTLE model, many apoptotic cells remained not phagocytosed in the hippocampus (**Fig 6H; S1 Table**). Some of the apoptotic cells were phagocytosed by terminal or en passant microglial branches (ball-and-chain mechanism) (**Fig 6I**), similar to phagocytosis by mouse microglia in vivo in physiological conditions, after LPS [9], or early after KA challenge (**Fig 3E**). In addition, we observed a unique type of microglial phagocytosis in the human brain in which several microglia formed a mesh surrounding the apoptotic cell, in an aster-like structure (**Fig 6J**). Furthermore, in all patients we found many instances where several microglia directly projected their processes towards nonapoptotic neurons (**Fig 6K and S8A Fig**), which can be interpreted either as the initiation of a phagoptotic process or of the aster-shaped phagocytosis uniquely found in the human tissue. Importantly, the three samples from MTLE patients analyzed showed a low Ph index (on average, 49 ± 4%; 26 ± 3% ball-and-chain, 23 ± 4% aster; **Fig 6L**), and a large proportion of apoptotic cells in close proximity to a microglial process (up to 67% of cells under 0.5μm; **Fig 6H and 6N**), similar to that of KA mice at 4 mpi. The density of microglial cells and the volume occupied by microglia were also consistent among patients (**Fig 6M and 6O**) and remarkably similar to those in our mouse model of MTLE at 4 mpi after the injection of KA.

To confirm this data, we obtained autopsy hippocampal tissue from epileptic patients (not MTLE) and nondemented controls from the Netherlands Brain Bank (**S8B and S8C Fig**). The long postmortem (PM) delays (ranging from 3 to 22 h) complicated the interpretation of the data, as phagocytosis was mostly absent in the autopsy tissue. The low phagocytosis in autopsy human tissue is in agreement with data obtained PM in the spinal cord of mice, where the microglial motility and damage response were strongly prevented as early as 3 h PM, likely due to a depletion of energy sources in the dead tissue [24]. Nonetheless, we found a significant decrease in the Ph index in the hippocampus of nondemented controls compared to epileptic patients (22 ± 3% versus 1.3 ± 1.3%, respectively from 6 control and 3 epileptic individuals; *p* = 0.002; **S1 Table**). Overall, our data demonstrate that microglial phagocytosis is impaired in human epilepsy.

### Phagocytosis Impairment Is Triggered by Widespread ATP Release during Seizures

**Fig 7:**
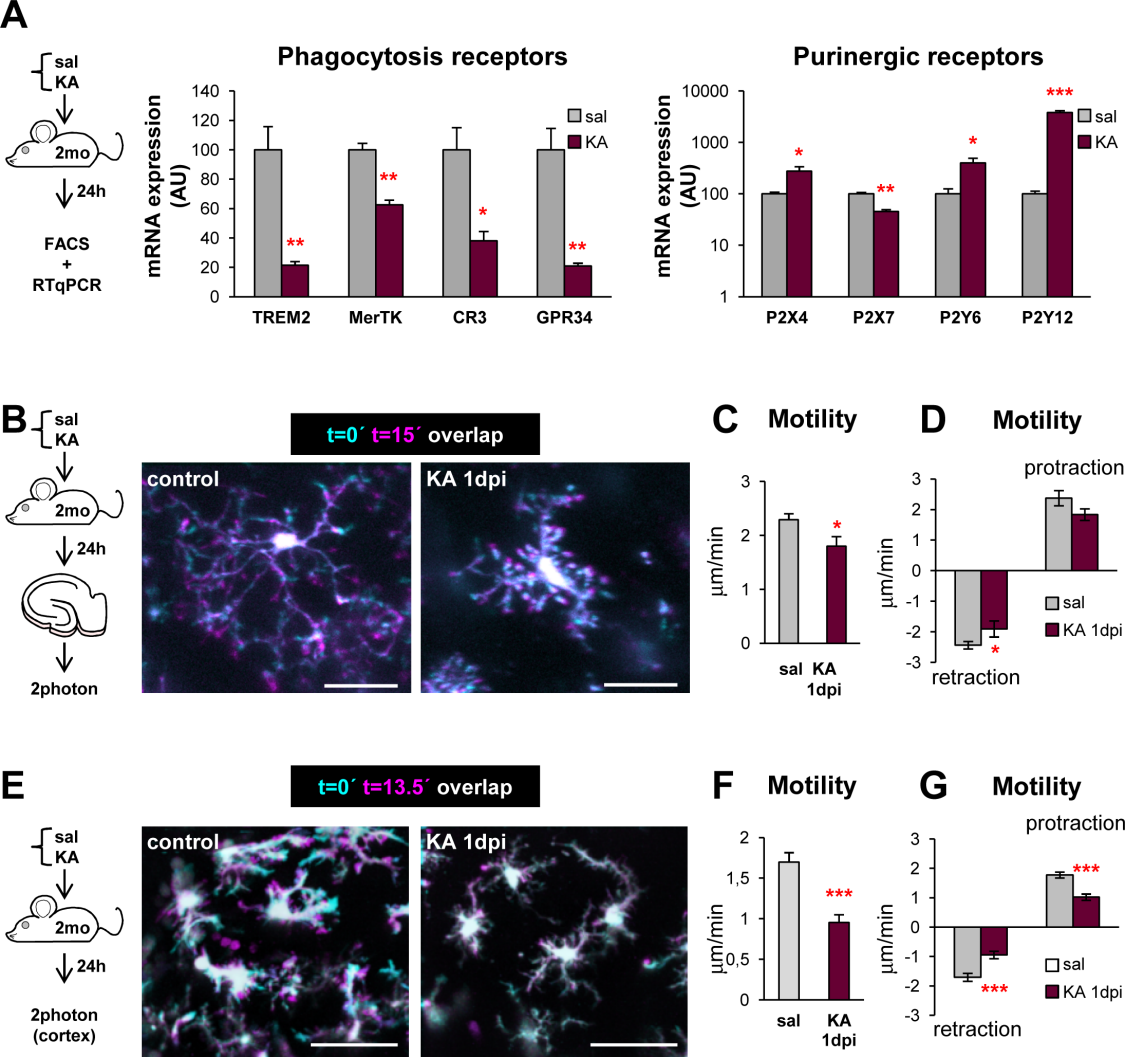
Early phagocytic impairment is related to reduced expression of phagocytosis receptors and reduced motility. (**A**) Experimental design and expression of phagocytosis and purinergic receptors by RTqPCR in FACS-sorted microglia from control and KA mice at 1 dpi (*n* = 3 from 8 pooled hippocampi). HPRT was used as a reference gene. (**B**) Experimental design and representative projections of 2-photon microscopy images of microglia at t0 (cyan) and 15 min later (magenta) from the DG of controls and KA-treated mice (1 dpi). (**C**) Motility of microglial processes by 2-photon microscopy in acute slices from CX3CR1^GFP/+^ mice after in vivo injection of KA (1 dpi; *n* = 4–5 cells from 3–4 mice per group). (**D**) Retraction and protraction of microglial processes by 2-photon microscopy in acute slices from CX3CR1^GFP/+^ mice after in vivo injection of KA (1 dpi). (**E**) Experimental design and representative projections of 2-photon images of microglia at t0 (cyan) and 13.5 min (magenta) in the cortex of controls and KA-treated mice (1 dpi). (**F**) Motility of microglial processes by 2-photon microscopy in the living cortex of CX3CR1^GFP/+^ mice after the injection of KA (1 dpi; *n* = 6 cells from 3 mice per group). (**G**) Retraction and protraction of microglial processes by 2-photon microscopy in the living cortex of CX3CR1^GFP/+^ mice after the injection of KA. Bars represent mean ± SEM. * indicates *p* < 0.05, ** indicates *p* < 0.01, and *** indicates *p* < 0.001 by Student´s *t* test (A, C, D). Scale bars = 20 μm (B), 50 mm (E). z = 50 μm (A), 40 μm (B). Underlying data is shown in **S1 Data**.

We next investigated potential mechanisms underlying the impairment of microglial phagocytosis in the acute phase of epilepsy. Such impairment occurred as early as 6 hpi, before a significant increase in the number of apoptotic cells and decreased microglial density were detectable (at 1 dpi; **Fig 3 and S3A Fig**). Strangely, we observed that in KA mice many nonphagocytosed apoptotic cells were localized in direct apposition to a microglial process (**Fig 3D and 3E**). While in control mice the average distance between an apoptotic cell and the closest microglial process was 1.3 ± 0.3 μm, this was increased significantly at 6 hdpi and 1 dpi following KA challenge (**S3F Fig**). In KA-treated mice at 1 dpi, 25% of nonphagocytosed apoptotic cells were 3–10 μm away, and up to 15% were over 10 μm away from a microglial process (**Fig 3L**). These results suggested two potential mechanisms for the phagocytosis impairment: a defect in recognition and phagocytosis initiation (which would result in apoptotic cells apposed to microglia but not phagocytosed) and a defect in microglial surveillance and/or targeting of apoptotic cells (which would result in far-off apoptotic cells). To verify the first hypothesis, we analyzed the microglial expression of main receptors involved in phagocytosis: triggering receptor expressed in myeloid cells 2 (Trem2) [25], Mer Tyrosine Kinase (MerTK) [26], complement receptor 3 (CR3) [27], and the G protein coupled receptor GPR34 [28]. We also analyzed the expression of main receptors for ATP and UTP, well-described apoptotic cell “find-me” signals, such as the ionotropic P2X_4_ and P2X_7_ and the metabotropic P2Y_6_ and P2Y_12_ receptors [29–31]. We acutely purified microglia from the hippocampus of control and KA mice at 1 dpi by fluorescent-activated cell sorting (FACS) and quantified the expression of these receptors by RTqPCR. We found that whereas P2X_4_, P2Y_6_, and P2Y_12_ receptors significantly increased, the apoptotic cell recognition receptors Trem2, MerTK, CR3, and GPR34 were significantly decreased in microglia from KA mice, explaining the deficient apoptotic cell targeting (**Fig 7A**).

We next explored the second hypothesis and reasoned that the impaired targeting could result from an impaired motility of the microglial processes, among other possible mechanisms. To assess the motility of microglial processes we resorted to an ex vivo approach using acute hippocampal slices and 2-photon microscopy [32,33]. As predicted, KA induced a 22% decrease in basal microglial motility at 1 dpi, mostly due to a decreased retraction of their processes (**Fig 7B–7D**), which could lead to a decreased surveillance capacity. To further test the impairment of microglial motility, we imaged the living cerebral cortex overlying the hippocampus, where we had previously detected phagocytosis impairment (**S2C Fig**). In the living cortex of KA-injected mice at 1 dpi, microglia showed a 37% decrease in their basal motility compared to saline-injected mice, which affected both the retraction and protraction (**Fig 7E–7G; S1 and S2 Movies**). Together, the decreased expression of phagocytosis receptors and the reduced motility would explain the defect in microglial phagocytosis of apoptotic cells observed after seizures.

**Fig 8:**
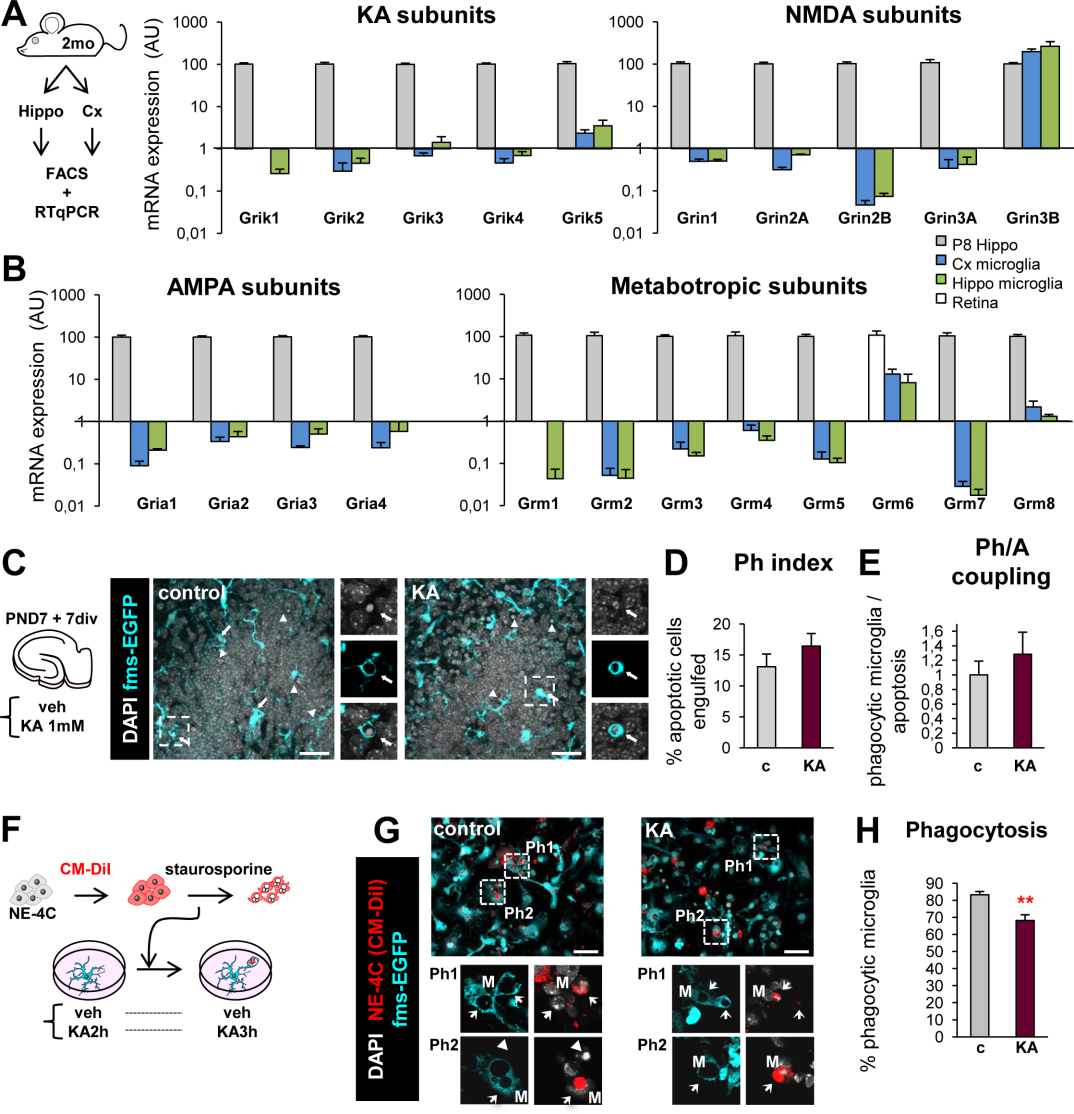
Phagocytosis impairment is not directly mediated by glutamate receptors on microglia. (**A, B**) Experimental design for RTqPCR expression of KA, NMDA, AMPA and metabotropic, receptor subunits in acutely purified microglia (FACS-sorted) from the hippocampus and the cortex of 2 mo mice (*n* = 4 samples of 8 pooled hippocampi and cortices each). The relative expression was compared to a positive control, a PND8 hippocampus, except for Grm6, where the retina from a 2-mo mouse was used. L27A was used as a reference gene. Amplification plots and denaturing curves for each target gene are shown in **S9 Fig**. (**C**) Experimental design and representative projections of confocal z-stacks of organotypic slices from fms-EGFP mice treated with vehicle (control) or KA (1 mM) for 6 h. The number of apoptotic cells, Ph capacity, and number of microglia in the DG is shown in **S10A–S10C Fig**. (**D**) Ph index in the DG organotypic slices (in % of apoptotic cells). (**E**) Ph/A coupling (in fold-change) in organotypic slices treated with KA. (**F**) Experimental design to test the effect of KA on microglial phagocytosis in vitro. Primary cultures were pre-treated with KA (1 mM) for 2 h prior to adding apoptotic NE-4C cells (treated with 5 μM CM-DiI for 25 min and 10 μM staurosporine for 4 h). NE-4C cells were left in the culture for another 3 h in the presence or absence of KA. (**G**) Representative confocal z-stacks of fms-EGFP^+^ microglia phagocytosing apoptotic CM-DiI^+^ NE-4C cells. (**H**) Percentage of phagocytic microglia in cultures (*n* = 2 independent experiments in triplicate). Bars represent mean ± SEM. ** indicates *p* < 0.01 by Student´s *t* test (H). Scale bars = 30 μm (C, G). z = 6.3 μm (F, J). Underlying data is shown in **S1 Data**.

We then asked whether KA could be acting directly on microglia to cause the observed phagocytosis impairment. We determined the expression of glutamate receptors in microglia ex vivo and assessed the direct effect of KA in organotypic slices and primary cultures. We performed a systematic analysis of the expression of all ionotropic and metabotropic glutamate receptor subunits by RTqPCR in fluorescence-activated cell sorting (FACS) purified microglia from the hippocampus and cortex of control 2-mo mice (**Fig 8A and 8B and S9 Fig**). We detected a residual expression of all ionotropic and metabotropic subunits in hippocampal and cortical microglia, unlikely to lead to the formation of functional receptors. In parallel, we tested the effect of KA (1 mM) in hippocampal organotypic slices, and found that it did not impair microglial phagocytosis of apoptotic cells (**Fig 8C–8E and S10A–S10C Fig**), likely because KA did not induce seizures in vitro. Finally, we tested the direct effect of KA on microglia in an in vitro model of phagocytosis, in which primary cultures of microglia derived from PND0 mice were fed with a neuronal cell line, NE-4C, previously treated with staurosporine to induce apoptosis. There, KA only produced a small but significant reduction in the percentage of phagocytic microglia (**Fig 8F–8H**). Therefore, the strong impairment of microglial phagocytosis that we observed in vivo after KA injection unlikely resulted from a direct effect of KA on microglial KA receptors.

**Fig 9:**
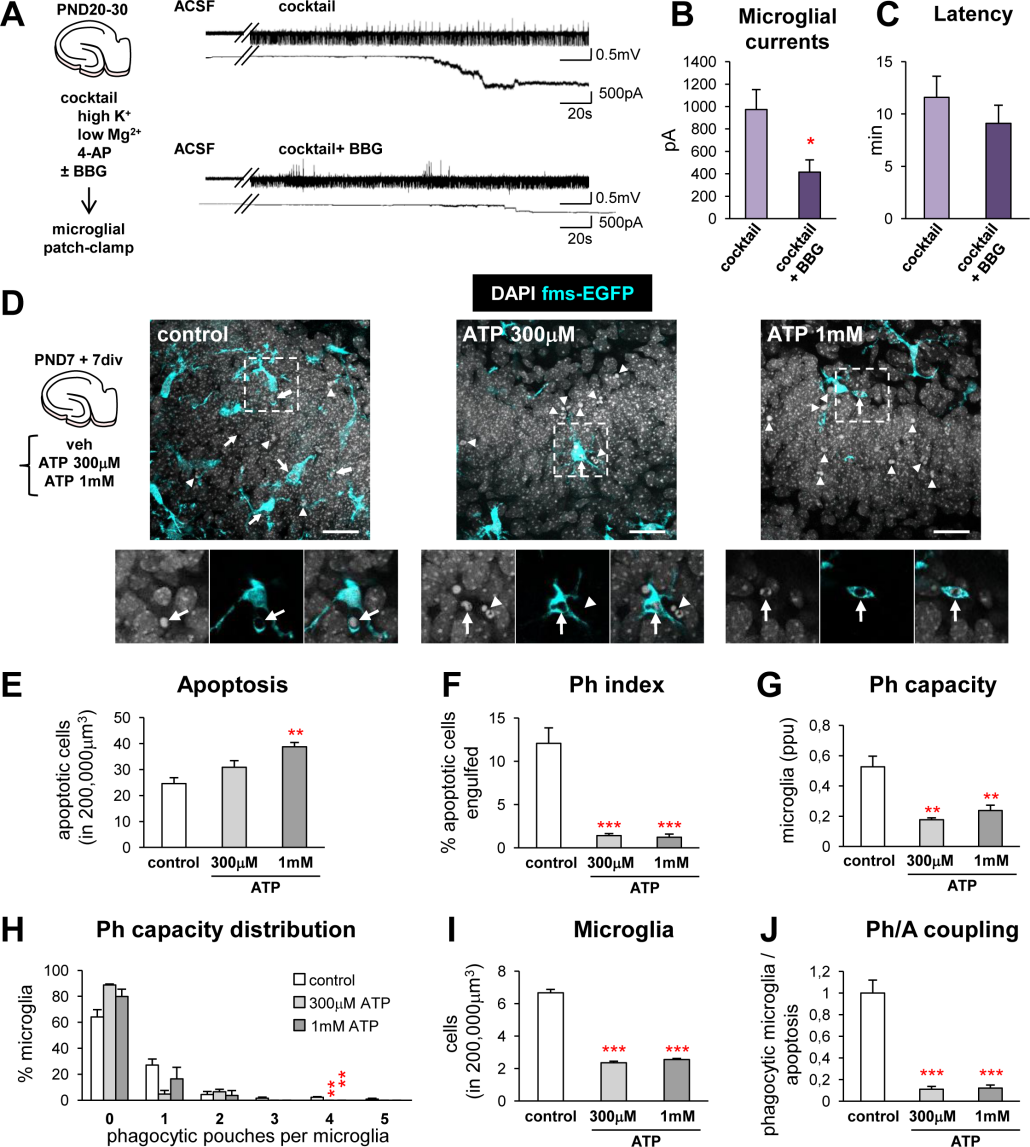
Seizures trigger ATP-mediated microglial activation and impairment of phagocytosis. (**A**) Experimental design used to induce seizures in acute hippocampal slices with an epileptogenic cocktail that included high K^+^, low Mg^2+^, and 4-AP in ACSF, in the presence or absence of the broad P2X receptor antagonist BBG (5 μM). Seizure activity was recorded in CA1, where it had higher amplitude than in the DG. Top, extracellular recording (mV) and bottom, simultaneous microglia patch clamp recording (pA) before and after seizure induction in the absence or presence of BBG. BBG did not alter seizure frequency or amplitude (**S10K–S10M Fig**). (**B**) Patch-clamp currents (in pA) induced in microglia by the seizure activity after the epileptogenic cocktail in the absence (control, *n* = 18 cells) or presence of the P2X antagonist BBG (*n* = 11 cells). (**C**) Latency (in minutes) of the currents induced in microglia by the seizure activity. (**D**) Experimental design and representative projections of confocal z-stacks of fms-EGFP organotypic slices treated with vehicle (control) or ATP (300 μM and 1 mM) for 4 h. Normal or apoptotic nuclear (pyknotic/karyorrhectic) morphology was visualized with DAPI (white) and microglia by the transgenic expression of fms-EGFP (cyan). The high magnification inserts show single images of apoptotic cells phagocytosed by microglia (arrows) or not phagocytosed (arrowheads). (**E**) Number of apoptotic cells in a 200.000 μm^3^ volume containing the DG in organotypic slices treated with ATP (*n* = 3 slices per group). (**F**) Ph index in organotypic slices (in %). (**G**) Weighted Ph capacity (in ppu) in organotypic slices. (**H**) Histogram showing the Ph capacity of microglia distribution (in % of cells) in organotypic slices treated with ATP. (**I**) Number of microglia in organotypic slices. (**J**) Ph/A coupling (in fold-change) in organotypic slices. Bars represent mean ± SEM. * indicates *p* < 0.05, ** indicates *p* < 0.01, and *** indicates *p* < 0.001 by Student´s *t* test (B) or by Holm-Sidak posthoc test after one-way ANOVA (E–J) was significant at *p* < 0.05. Scale bars = 30 μm. z = 6.3 μm. Underlying data is shown in **S1 Data**.

A strong candidate to mediate the effects of seizures is the extracellular nucleotide ATP. In addition to being released by apoptotic cells [2], ATP is also released during seizures, either from neurons or from astrocytes [34,35] and mediates the effects of glutamate on microglial motility in retinal explants [36]. Direct measurement of the ATP release in vivo is complicated by the fact that is rapidly degraded by several ectonucleotidases. Thus, we resorted to indirectly determining the action of ATP released during seizures on microglia in vitro. We induced seizures using a cocktail containing low Mg^2+^, high K^+^, and the nonselective blocker of voltage-dependent potassium channels 4-aminopyridine (4-AP). This cocktail led to impaired microglial phagocytosis in hippocampal organotypic slices as early as 1 h, as microglia failed to proportionally increase their Ph capacity in response to the increase in apoptosis, further confirming our in vivo data that seizures impair phagocytosis in mouse and human MTLE (**S10D–S10J Fig**). Next, we treated acute hippocampal slices with the epileptogenic cocktail and recorded microglial currents by patch-clamp. After a latency period of 11 ± 2 min, seizures induced large inward currents in microglia that were blocked by the broad purinergic P2X receptor antagonist BBG (Brilliant Blue G), (**Fig 9A–9C**), indicating a cationic current through these channels. BBG did not alter the frequency or amplitude of the epileptic discharges (**S10K–S10M Fig**). Overall, these data demonstrate that microglia sense seizures via ATP.

Because disrupting ATP gradients by saturating the cortical tissue with a high concentration bath of ATP decreases the extension rate of microglia towards a laser-induced lesion [4], we tested the hypothesis that large concentrations of ATP would disrupt the local “find-me” gradients in hippocampal organotypic slices and impair phagocytosis. Bathing the slices in ATP (1 mM) significantly increased the number of apoptotic cells in the slice (**Fig 9D and 9E**). This effect was possibly due both to direct neuronal death [37,38] and a block of microglial phagocytosis. The Ph index was substantially decreased (**Fig 9F**) as a result of decreased Ph capacity (**Fig 9G and 9H**) and decreased microglial density (**Fig 9I**). We did not observe microglial apoptosis induced by ATP but instead, microglia migrated towards the edges of the slice, an effect that we attribute to the chemotactic nature of ATP. Ultimately, the Ph/A coupling was lost (**Fig 9J**). Overall, these data indicate that ATP impairs microglial phagocytosis and suggest a yet unexplored mechanism underlying the early phagocytic impairment in experimental MTLE.

**Fig 10:**
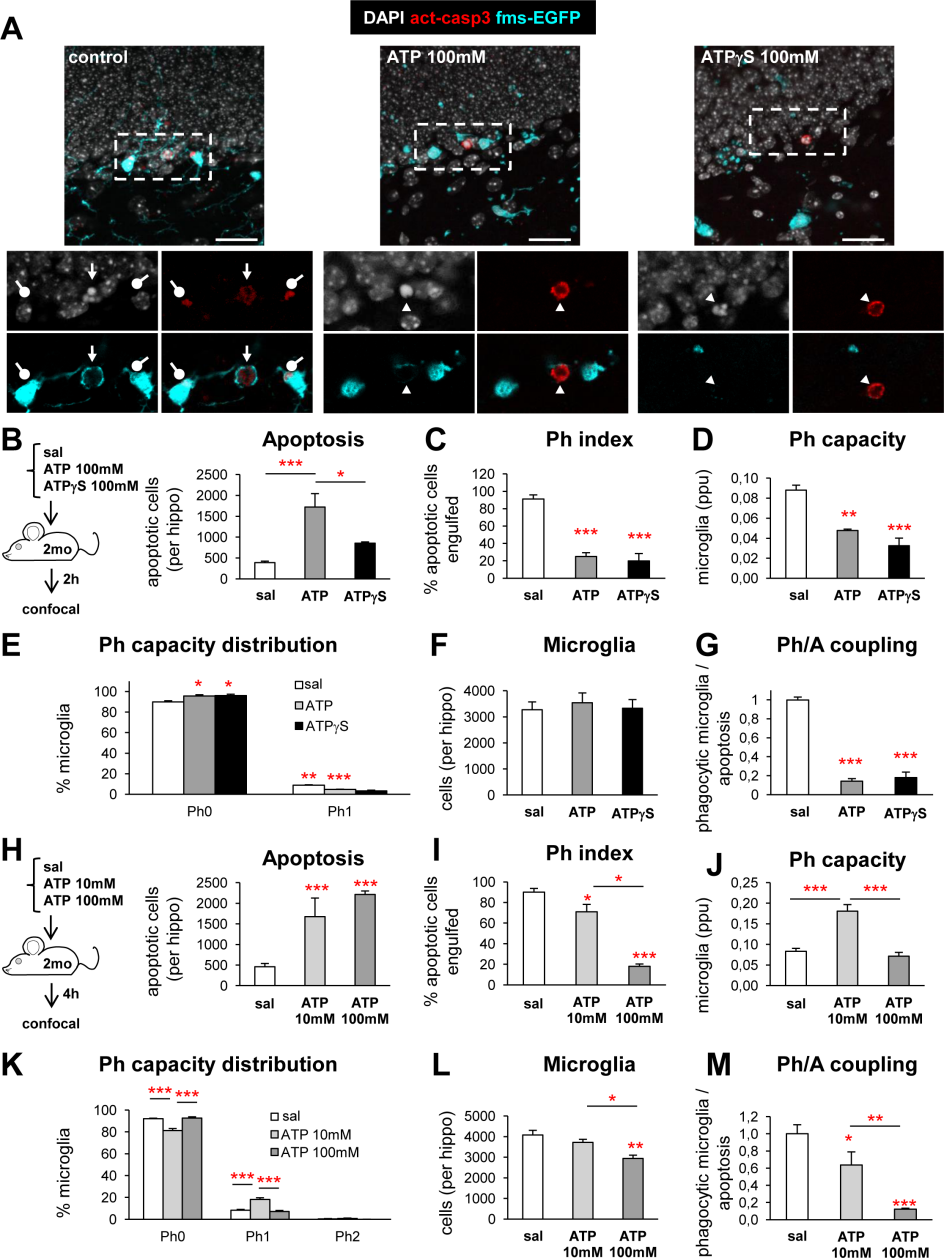
ATP impairs microglial phagocytosis in vivo. (**A**) Representative confocal z-stacks of saline, 100 mM ATP and 100 mM ATPγS (2 hpi) DG labeled with DAPI (nuclear morphology, white), activated caspase 3 (act-casp3^+^, red, for apoptotic cells), and fms-EGFP (cyan, microglia). Arrow points to a phagocytosed apoptotic cell, whereas arrowheads point to nonphagocytosed apoptotic cells. Activated-caspase 3 puncta within microglia are labeled with a round-ended arrow. (**B, H**) Experimental designs (**B**, 100 mM of ATP and ATPγS, 2 h; **H**, 10 and 100 mM ATP, 4 h; *n* = 3–4 per group) and number of apoptotic (pyknotic/karyorrhectic and act-casp3^+^) in the septal DG (*n* = 3–4 per group). No changes in the volume of the DG were found in either experiment (**S11C Fig**). (**C, I**) Ph index in the septal DG (in % of apoptotic cells). (**D, J**) Weighted Ph capacity of hippocampal microglia (in ppu). (**E, K**) Histogram showing the Ph capacity distribution of microglia (in % of cells) in the septal DG. (**F, L**) Total number of microglial cells (fms-EGFP^+^) in the septal DG. (**G, M**) Ph/A coupling (in fold change) in the septal DG. Bars represent mean ± SEM, * indicates *p* < 0.05, ** indicates *p* < 0.01, and *** indicates *p* < 0.001 by Holm-Sidak posthoc test after one-way ANOVA were significant at *p* < 0.05. Scale bars = 50 μm, z = 11.9 μm (control, ATP), 9.8 μm (ATPγs). Inserts are single plane images of the corresponding confocal z-stacks. Underlying data is shown in **S1 Data**.

To further support our hypothesis that widespread release of ATP impairs microglial phagocytosis, we injected ATP and its nondegradable analog ATPγS directly into the DG and assessed their effect on microglial phagocytosis at 2 hpi. We injected a relatively high dose (100 mM) compared to conventional doses used in vitro, as in our organotypic slices experiments, to account for their diffusion over the whole hippocampus (spanning an area of several cubic mm). Indeed, we found a noticeable alteration in microglial morphology with processes retraction throughout the septal hippocampus, largely restricted to the DG (**S11A Fig**). No signs of cell death or shrinkage due to a potential osmosis imbalance were observed at the injection site in spite of the high osmolarity of the injected solutions (PBS: 286 mmol/kg; ATP: 473 mmol/kg; ATPγS: 635 mmol/kg), likely because of their diffusion over the hippocampal parenchyma. ATP, but not ATPγS, resulted in an increased number of apoptotic cells in the DG (**Fig 10A and 10B**). Both treatments induced a significant reduction of the Ph index (**Fig 10C**) and Ph capacity (**Fig 10D and 10E**) without altering the number of microglia (**Fig 10F**), ultimately resulting in a strong reduction of the Ph/A coupling (**Fig 10G**). Nonetheless, apoptotic microglia could be occasionally observed in the ATP (126 ± 46 apoptotic microglia per septal hippocampus) but not in the ATPγS- treated DG.

To disregard the possibility that changes in phagocytosis efficiency in ATP-treated mice were the result of reduced microglial viability, we performed a second experiment with 10 mM (304 mmol/kg) and 100 mM ATP. It is also important to note that, as the average clearance time is 1.2–1.5 h [9], at 2 hpi we could detect only a fraction of the cells that had impaired recognition, as the cells that started phagocytosis before the injection would still be in the process of degrading the apoptotic cell. Thus, we decided to analyze microglial phagocytosis at a later time point (4 hpi) to let them fully digest their prior cargo. In contrast, in this later time point degradation of the injected ATP by ectonucleotidases was more likely to occur. At 4 hpi, both 10 and 100 mM ATP increased the number of apoptotic cells in the DG (**Fig 10H and S11B Fig**) and, as expected, the phagocytosis impairment indicated by the drop in the Ph index was more obvious in mice treated with the higher dose (**Fig 10I**). Following 100 mM ATP, the Ph index dropped and the Ph capacity remained constant, indicating a recovery from the 2 hpi likely because of a wash-out or degradation of the injected ATP (**Fig 10I and 10J**). The number of microglia decreased with ATP 100 mM (**Fig 10L**), indicating the expected loss of viability. Microglial apoptosis was observed at 100 mM ATP (34 ± 16 apoptotic microglia per septal DG) but not at 10 mM ATP. Furthermore, at 10 mM ATP, the Ph index dropped and the Ph capacity increased but not sufficiently to counteract the increase in apoptosis, without affecting microglial numbers (**Fig 10L**), ultimately resulting in a decreased Ph/A coupling (**Fig 10M**). We finally tested the alternative hypothesis that impaired recognition in epileptic mice was due to a defective signaling from apoptotic cells, possibly due to an altered expression of pannexin, one route through which ATP is released [39]. However, we found a low expression of pannexin throughout the hippocampus and no expression in apoptotic cells, either phagocytosed or not, both in control or KA-treated mice (**S12 Fig**). Thus, in the hippocampus apoptotic cells may signal to microglia via mechanisms unrelated to pannexin channels. Overall, the results obtained with ATPγS (100 mM, 2 h) and ATP (10 mM, 4 h) confirm our in vitro data and demonstrate that disrupting ATP gradients impairs microglial phagocytosis.

### Seizures Lead to the Accumulation of Nonphagocytosed Apoptotic Cells In Vivo

The impairment in microglial phagocytosis should result in an accumulation of apoptotic cells. To directly test this hypothesis, we estimated the clearance time of well-identified cell populations undergoing apoptosis. Our data showed that the majority of apoptotic cells at 1 dpi after KA were located in the SGZ (**Fig 3B**), the niche where new neurons are born, in agreement with previous publications showing that seizures lead to apoptosis of newborn cells [40,41]. Thus, we studied the effect of KA-induced seizures on the apoptosis and survival of newborn cells, and focused on their previously identified early (3 d old, do) and late (8 do) critical periods of survival [9,42]. A single injection of the thymidine analog bromo-deoxyuridine (BrdU, 150 mg/kg) was administered 2 or 7 d prior to the injection of saline or KA, and mice were killed 1 d later (KA 1 dpi). Unexpectedly, we found no significant changes in the number of 3 and 8 do BrdU^+^ cells, indicating that KA did not affect their survival (**S13A and S13B Fig**).

**Fig 11:**
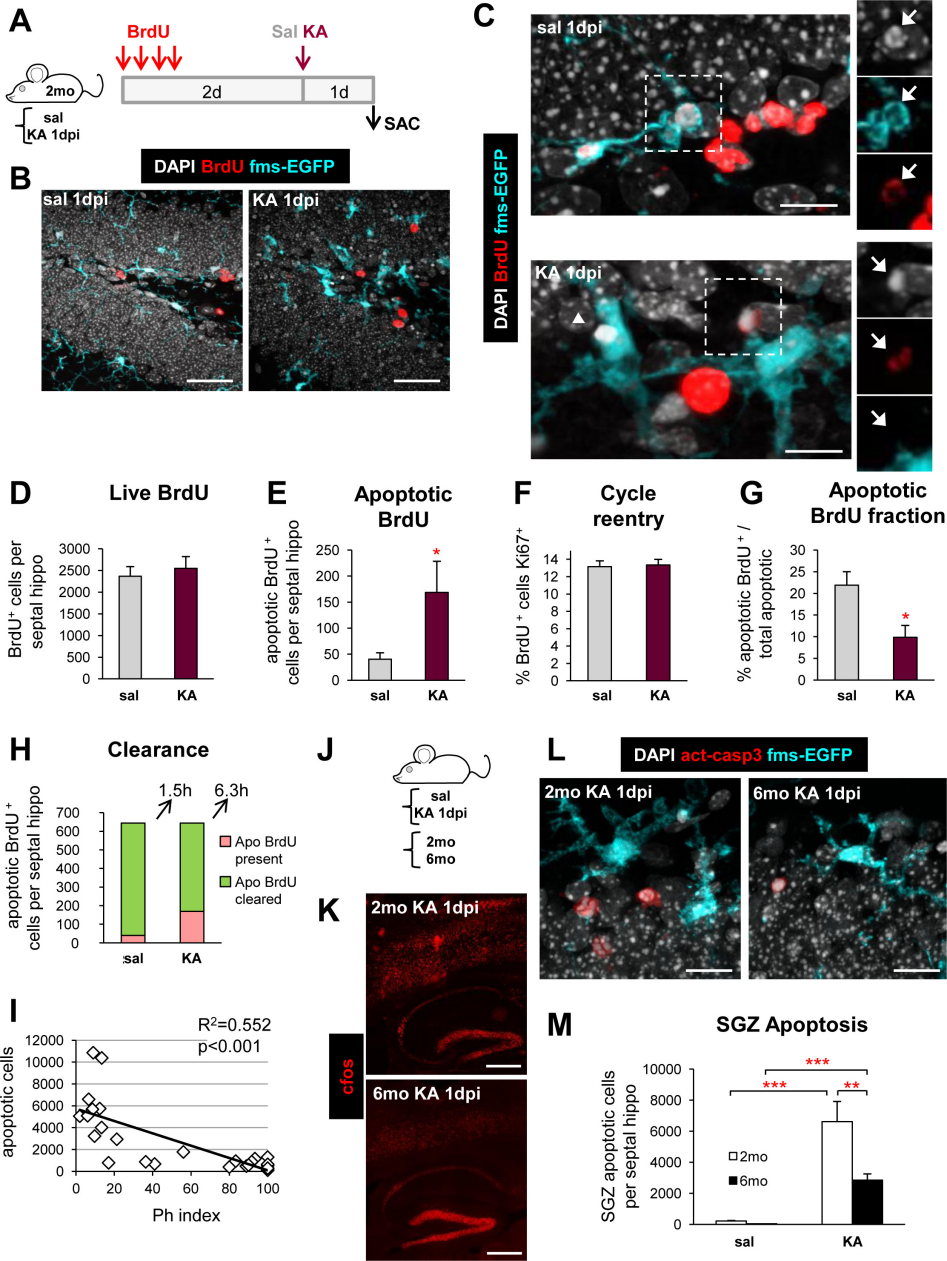
Microglial phagocytic impairment leads to delayed clearance of apoptotic cells at 1 dpi. (**A**) Experimental design used to analyze the survival of 3 do cells after the injection of saline (*n* = 7) or KA (*n* = 8) in mice. (**B**) Representative confocal z-stacks of the DG of control and KA-injected mice (1 dpi). The damage induced by KA was evidenced by the presence of cells with abnormal nuclear morphology (DAPI, white), and the altered morphology of microglia (fms-EGFP^+^, cyan). (**C**) Representative confocal images of 3 do apoptotic (pyknotic, DAPI, white) cells labeled with BrdU (red; arrows) in the SGZ of the hippocampus of saline and KA-injected mice at 1 dpi. In the saline mouse, the BrdU^+^ apoptotic cell, next to a cluster of BrdU^+^ cells, was phagocytosed by a terminal branch of a nearby microglia (fms-EGFP, cyan), whose nucleus was also positive for BrdU. In the KA mouse, the apoptotic BrdU^+^ cell was not phagocytosed by microglia. A nearby apoptotic cell (BrdU^-^; arrowhead) was partially engulfed by microglia. (**D**) Total number of live 3 do BrdU^+^ cells (nonapoptotic) in the septal hippocampus after treatment with KA. The total number of 3 do and 8 do BrdU^+^ cells by a single BrdU injection in saline and KA-injected mice is shown in **S13A and S13B Fig**. (**E**) Total number of apoptotic 3 do BrdU^+^ cells in the septal hippocampus after treatment with KA. (**F**) Percentage of 3 do BrdU^+^ cells that re-enter cell cycle, assessed by their colabeling with the proliferation marker Ki67 after treatment with KA. Representative confocal z-stacks of BrdU/Ki67 cells are found in **S13C Fig**. (**G**) Percentage of apoptotic BrdU^+^ cells over total apoptotic cells in the septal hippocampus. (**H**) Estimated clearance of apoptotic cells in the septal hippocampus. The total number of apoptotic BrdU^+^ (from E) present in the tissue was added to the number of estimated apoptotic BrdU^+^ cells that had been cleared. In saline mice, this number was calculated using the clearance time formula shown in Methods with a clearance time of 1.5 h [9]. As the total number of cells should be identical in saline and KA mice, the number of cleared apoptotic cells in KA mice was calculated as the difference between the total (in saline) and the number of present apoptotic cells (in KA). From here, we calculated a new clearance time using the same formula as in saline mice, of 6.3 h. (**I**) Linear regression analysis of the relationship between apoptosis and phagocytosis (Ph index) in saline and KA-injected mice (6 hpi and 1 dpi). (**J**) Experimental design used to compare SGZ apoptosis induced by KA at 1 dpi in young (2 mo) and mature (6 mo) mice. (**K**) Representative epifluorescent tiling image of the hippocampus and surrounding cortex of 2 and 6 mo mice injected with KA at 1 dpi stained with the neuronal activation marker c-fos. The same pattern of expression was found in young and mature mice throughout the DG, CA2, CA1 and the above cortex. (**L**) Representative confocal z-stacks of the apoptotic (pyknotic, white; act-casp3^+^, red) cells in the SGZ of the hippocampus of 2 mo and 6 mo mice injected with KA (1 dpi). The microglial phagocytosis impairment was similar in the two age groups (**S13D Fig**). (**M**) Total number of apoptotic cells in the SGZ of 2 and 6 mo mice treated with saline or KA (1 dpi; *n* = 4–5 per group). Bars show mean ± SEM. * indicates *p* < 0.05, ** *p* < 0.01, and *** *p* < 0.001 by Student´s *t* test (E, G) or by Holm-Sidak posthoc test after one-way ANOVA (M) was significant at *p* < 0.05. Scale bars = 50 μm (B), 20 μm (C), 500 μm (K), 25 μm (L). z = 14 μm (B), 12.6 μm (C, sal), 15.4 μm (C, KA), 25 μm (L). Underlying data is shown in **S1 Data**.

To increase the probability of observing apoptotic BrdU^+^ cells, we switched to a semicumulative BrdU administration paradigm and focused on the early critical period (3 d) (**Fig 11**). We administered BrdU every 2 h for 6 h (**Fig 11A**) 2 d prior to KA injection and quantified the number of live and apoptotic BrdU^+^ cells 1 day later (KA 1 dpi) (**Fig 11B and 11C**). Again, we found no significant changes in the number of live BrdU^+^ cells (**Fig 11D**), but a seemingly contradictory significant increase in the number of apoptotic BrdU^+^ cells (**Fig 11E**). To exclude the possibility that an (undetectable) loss of BrdU^+^ cells was compensated by their increased proliferation, we analyzed their reentry into the cell cycle by calculating the percentage of BrdU^+^ cells expressing the cell division marker Ki67. We found no evidence of increased proliferation of the 3 do BrdU^+^ cell population due to KA injection, as the percentage of BrdU^+^ cells colabeled with the proliferation marker Ki67 remained unchanged between control and KA-injected mice (**Fig 11F and S13C Fig**). Together, these results demonstrate that the increase of apoptotic 3 do BrdU^+^ cells was not due to de novo apoptosis, but rather, to the accumulation of nonphagocytosed cells that were already undergoing apoptosis prior to the KA injection. Nonetheless, the apoptotic BrdU^+^ fraction significantly decreased in KA mice (**Fig 11G**), suggesting that apoptosis preferentially targeted cell populations other than the 3 do cells in the KA-injected hippocampus. Thus, the rise in apoptotic cells in KA at 1 dpi is due both to an accumulation of nonphagocytosed 3 do cells and de novo apoptosis of other populations.

For the 3 do cells, we reasoned that if KA was not affecting their survival (as there was no change in the number of live BrdU^+^ cells), the total number of apoptotic BrdU^+^ cells (present and cleared) should be identical in both control and KA mice, and used this information to estimate the clearance time in KA mice. The number of cleared apoptotic cells, i.e., the number of apoptotic cells no longer present in the tissue and eliminated by phagocytosis, can be estimated using the clearance time formula (see Materials and Methods) and the estimated clearance time in physiological conditions of 1.5 h [9]. To obtain the total number of apoptotic BrdU^+^ cells (present and cleared), we estimated the number of cleared BrdU^+^ apoptotic cells in saline mice and added it to the number of BrdU^+^ apoptotic cells present in saline mice (**Fig 11H**). To obtain the estimated number of cleared cells in KA mice, we then subtracted the number of BrdU^+^ apoptotic cells in KA mice from the above amount of total apoptotic BrdU^+^ cells. This subtraction allowed us to estimate a new clearance time of 6.3 h in KA mice (**Fig 11H**), which represents the average time at the population level required for an apoptotic cell to be eliminated by the dysfunctional microglia or the recruited astrocytes or neuroblasts. Furthermore, the decay in the Ph index predicted up to 52% of the variation in the number of apoptotic cells using a linear regression analysis (*p* < 0.001) in all saline and KA mice used (6 hpi and 1 dpi) (**Fig 11I**), providing further evidence that the impairment in phagocytosis is linked to an accumulation of apoptotic cells.

Finally, we tested the effect of KA on apoptosis in young (2 mo) and mature (6 mo) animals, in which there are fewer neuroprogenitors and therefore fewer newborn cells [43]. Because in the SGZ the vast majority of apoptotic cells are newborn cells [9], we expected to see a reduction in SGZ apoptotic cells in older mice as a consequence of the reduced neurogenesis. After KA, both young and mature animals reached level 3–4 in the Racine scale and at 1 dpi had a similar level and pattern of activation of the hippocampal circuitry (**Fig 11J and 11K**), as determined by staining with c-fos, an immediate early gene that has been used as an indirect marker of neuronal activation [44]. In addition, at 1 dpi after KA, microglial phagocytosis was similarly impaired in 2 and 6 mo mice (**S13D Fig**), but as expected, there were fewer SGZ apoptotic cells in 6 mo than in 2 mo mice (**Fig 9L and 9M**). This difference in apoptosis between young and mature mice can be attributed to the reduced proliferation and neurogenesis found in mature animals, confirming our hypothesis that the rise of apoptotic cells in the SGZ induced by KA is largely due to accumulation of the nonphagocytosed newborn cells that undergo apoptosis in physiological conditions.

### Microglial Phagocytic Impairment Correlates with Inflammation

**Fig 12:**
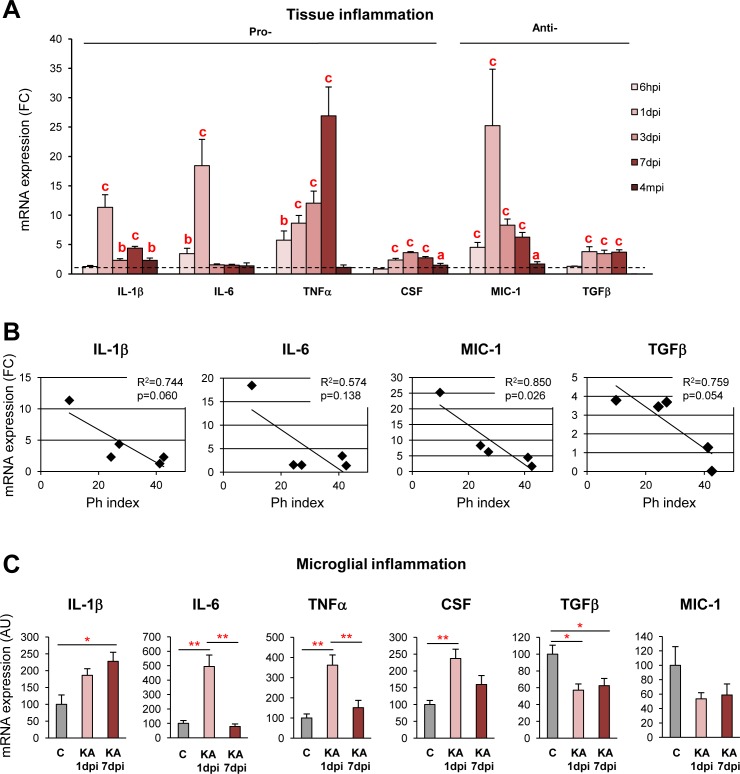
Phagocytosis impairment correlates with inflammation in mouse MTLE. (**A**) RTqPCR quantification of a panel of pro- and anti-inflammatory cytokines in the hippocampus of mice injected with saline or KA over a time course. Expression was normalized with the reference gene L27A and expressed as fold change (FC) over the saline injected mice (dashed line). The expression of the cytokines was linearized by a logarithmic transformation; only significant interactions are shown. (**B**) Linear regression analysis of the relationship between the average expression of tissue cytokines (IL-1β, IL-6, MIC, TGFβ) and average Ph index in KA-injected mice along the time course. The Ph index explained a large percentage of the variation of the expression of these cytokines, although significance at *p* = 0.05 was only reached for MIC-1 likely due to the small number of time points analyzed. (**C**) RTqPCR quantification of a panel of pro- and anti-inflammatory cytokines in FACS-sorted fms-EGFP^+^ microglia. In A and C data are shown as mean ± SEM. a, * indicates p < 0.05; b, ** indicates p < 0.01; and c,*** indicates p < 0.001 by Holm-Sidak posthoc test compared to their respective time point controls, after one-way ANOVA was significant at p < 0.05. Underlying data is shown in **S1 Data**.

We reasoned that, because phagocytosis of apoptotic cells is actively anti-inflammatory in vitro [45], the impairment of phagocytosis should correlate with the development of an inflammatory response. We tested this hypothesis by analyzing the expression of a panel of pro- and anti-inflammatory cytokines by RTqPCR in hippocampal tissue samples and in microglia from acutely purified microglia along the time course (6 hpi to 4 mpi) (**Fig 12**). At the tissue level, we found that proinflammatory interleukin 1 beta (IL-1β) and interleukin 6 (IL-6) as well as of anti-inflammatory macrophage inhibitory cytokine 1 (MIC-1) peaked at 1 dpi and decreased afterwards up to 4 mpi (**Fig 12A**), a pattern that paralleled the impairment of phagocytosis over time. In agreement, the average expression of these cytokines as well as anti-inflammatory TGFβ correlated with the Ph index over the 4 mpi time course (**Fig 12B**). We next compared the expression of these cytokines in FACS-sorted microglia from KA-treated mice. At 1 dpi, microglia from KA mice expressed the highest levels of the proinflammatory TNFα and IL-6 (IL-1β showed a strong tendency but was not significant), as well as CSF, and low levels of the anti-inflammatory TGFβ (MIC-1 showed a strong tendency but was not significant) compared to microglia from control mice. At 7 dpi, microglia from KA mice expressed higher levels of IL-1β and TNFα, and lower levels of TGFβ (**Fig 12C**). These data demonstrate that the impaired microglia are in a proinflammatory state.

## Discussion

**Fig 13:**
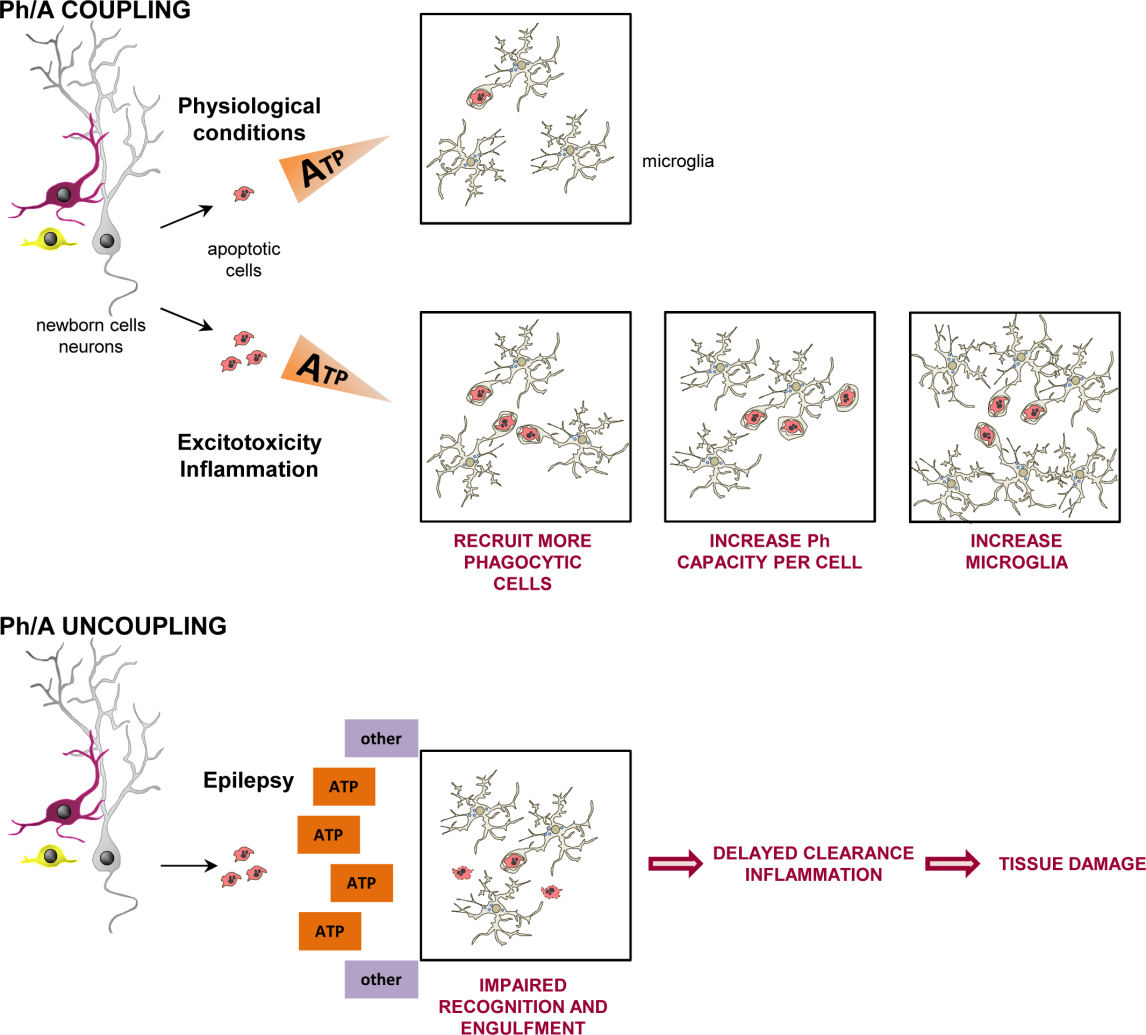
Microglial phagocytosis/apoptosis coupling in health and disease. In physiological conditions as well as during excitotoxicity and inflammation, microglial phagocytosis is tightly coupled to apoptosis due to “find-me” signals released by apoptotic cells, such as ATP. Microglia display a combination of three adaptation strategies to boost their phagocytic efficiency: recruit more phagocytic cells, increase the phagocytic capacity per cell, and/or increase the number of cells. In contrast, neuronal hyperactivity induced by seizures leads to a widespread release of ATP, among other possible signals, and interferes with the ability of microglia to recognize and engulf apoptotic cells, resulting in their delayed clearance. Because phagocytosis is actively anti-inflammatory, the phagocytosis impairment was associated with the production of proinflammatory, epileptogenic cytokines.

In the present study, we examined and quantified for the first time the microglial phagocytic behavior in times of brain distress and report the following original findings (**Fig 13**). First, we have uncovered a generalized response of microglia to brain damage by excitotoxicity or inflammation in which they plastically adapt their phagocytic efficiency to the amount of apoptosis. Second, we have revealed an unexpected chronic impairment of microglial phagocytosis in an experimental model of MTLE as well as in hippocampal tissue resected from pharmaco-resistant MTLE patients. Third, we show that the microglial phagocytic impairment is not directly due to KA receptors on microglia but is rather a complex phenomenon related to an impaired recognition as well as impaired motility and targeting, mediated at least partially by altered ATP microgradients. Fourth, we demonstrate that the microglial phagocytic impairment leads to the accumulation of apoptotic cells and contributes to the development of an inflammatory response in the mouse model of MTLE. Overall, the data presented herein illuminate a novel generalized response of microglia to phagocytic challenge in the DG. Importantly, we have observed a similar degree of impairment in CA regions and the cortex, although in these latter regions the lack of apoptotic cells implies that the basal phagocytic efficiency could not be determined. In addition, our data compellingly demonstrates that the impairment of microglial phagocytosis is a novel mechanism contributing to the pathophysiology of MTLE. Ultimately, the efficiency of microglial phagocytosis is a decisive factor regulating the dynamics of neuronal death in the diseased brain.

### Phagocytosis/Apoptosis Coupling in Health and Disease

In contrast to the long-standing assumption that phagocytosis is executed only by ameboid-shaped microglia [46], we initially found that in physiological conditions, phagocytosis is efficiently enacted by unchallenged, ramified, surveillant microglia. Using as a model the adult hippocampal neurogenic cascade, where newborn neurons undergo apoptosis throughout adulthood, we previously found that the vast majority of apoptotic cells are in the process of being engulfed by microglia (Ph index > 90%) through a ball-and-chain mechanism and a short clearance time (under 1.5 h) [9]. Thus, in physiological conditions, the high efficiency of microglial phagocytosis leads to a large underestimation of the total amount of apoptosis.

We have now studied the microglial response to phagocytic challenges and found a generalized microglial response. When subjected to a phagocytic challenge induced by acute or chronic inflammation or excitotoxicity, microglia stood up to the increased apoptosis combining three different strategies: recruiting more phagocytic cells, increasing the phagocytic capacity of each cell, and/or increasing microglial numbers. The combination of these three adaptation strategies allowed microglia to boost its phagocytic efficiency and match the apoptotic challenge, therefore maintaining the phagocytosis/apoptosis ratio. Furthermore, hippocampal microglia displayed up to four phagocytic pouches in vivo and up to seven in organotypic slices, although most cells had a single pouch and many cells remained nonphagocytic. Therefore, microglia have an enormous reservoir for phagocytosis that can be reached by recruiting 100% of cells with their maximum Ph capacity. This data suggests that enhancing phagocytosis could be a novel therapeutic approach to accelerate tissue recovery after brain injury.

### Neuronal Hyperactivity Leads to Phagocytosis–Apoptosis Uncoupling In Vivo

To our surprise, the microglial phagocytic potential was not employed to counteract the damage resulting from neuronal hyperactivity in a mouse model of MTLE. In contrast, we found an impairment of microglial phagocytosis, as shown by decreased Ph capacity and Ph index, which was not compensated by recruiting other phagocytic cells, such as astrocytes or neuroblasts. This impairment was not simply an inability of microglia to cope with too much apoptosis because it occurred as early as 6 hpi after KA, before any significant accumulation of apoptotic cells took place, and because microglia dispatched this amount of apoptosis in other conditions (for instance, LPS in 1 mo mice). Microglial phagocytosis was not impaired by inflammation nor by excitotoxicity per se, as shown above, and we thus postulated that the impairment was related to the KA-induced seizures.

However, the impairment was unlikely a direct effect on microglia of either KA or glutamate released during seizures, as microglia expressed residual levels of ionotropic and metabotropic glutamate subunits, in agreement with previous reports showing the lack of functional receptors in microglia in acute hippocampal slices [47] or retinal explants [36]. Furthermore, KA had a very small effect on phagocytosis of apoptotic cells in primary cultures and no effect in organotypic slices. Although KA is known to induce membrane ruffling and morphological alterations in cultured microglia [48], which would explain our results in primary cultures treated with KA, whether microglia express functional glutamate receptors in vivo is still under discussion [36,47,49–52].

Nonetheless, the level of neuronal activity is known to affect microglial motility and behavior, acting via extracellular ATP. In retinal explants and acute hippocampal slices, neuronal glutamate signaling via NMDA receptor activation leads to the release of ATP, which in turn alters microglial motility and morphology [36,52,53] and triggers microglial process convergence towards neuronal dendrites [54]. ATP is also released in large amounts during seizures in vivo and in vitro [34,35], although it has not been directly tested in our KA model due to the limited tools to measure extracellular ATP levels in vivo. Nonetheless, here we demonstrate that in vitro microglia acutely sense seizures, resulting in large inward currents that depend at least partially on P2X receptors, similar to those observed during ischemia [55]. Interestingly, we observed a delayed microglial response (11 min latency time) that is consistent with the time it takes to reach the maximum ATP release evoked by depolarization [56]. In addition to being a neuro- and gliotransmitter, ATP is a well-known “find-me” signal released by apoptotic cells [2,57] and mediates the rapid attraction of microglial processes towards laser-induced injuries [4]. In spite of the complex metabolism and signaling of released nucleotides, we have mirrored the seizure-induced phagocytosis impairment by disrupting ATP gradients in organotypic slices and in vivo. The KA-triggered microglial phagocytosis impairment is consistent with a seizure-related widespread release of ATP that disrupts “find-me” signaling gradients and turns microglia “blinded” to apoptotic cells.

ATP signals to microglia on a plethora of promiscuous P2X (ionotropic) and P2Y (metabotropic) receptors and is degraded by extracellular ectonucleotidases to adenosine, whose receptors are expressed by microglia as well [51]. While the full pharmacological characterization of the seizure-induced current and phagocytosis impairment will be carried out in future studies, we have evidence that several purinergic receptors might be involved in these phenomena, as we have detected a down-regulation of P2X_7_ and up-regulation of P2X_4_, P2Y_6_, and P2Y_12_ in microglia at 1 dpi after the KA injection. In agreement, seizures induce changes in the intrinsic electrophysiological properties of microglia (input resistance, membrane capacitance) and an enhanced response to ATP and UDP that are mediated by P2X_4_, P2Y_6_, and P2Y_12_ receptors, respectively [58,59]. Similar to ATP, UDP is a “find-me” signal released by apoptotic cells [60], and P2Y_6_ antagonists prevent the phagocytosis of microbeads injected into the cortex of KA-treated mice [30]. This paper also showed that the systemic injection of KA increased the capacity of cortical microglia to phagocytose latex microbeads [30], in apparent disagreement with our data. However, the mechanisms of engulfment of beads and apoptotic cells are largely different [60]. Importantly, latex microbeads do not release “find-me” signals that trigger microglial chemotaxis, a fact that would explain the discrepancy. Overall, our results show that neuronal hyperactivity interferes with the apoptotic cell “find-me” gradients by sending competing ATP signals that microglia cannot discriminate.

In agreement, we have observed a large proportion of microglia located far away from the apoptotic cells in KA mice at 6 hpi and 1 dpi, suggesting a decreased surveillance capacity. Consistently, we found a decreased microglial density in the DG, and overall motility of microglia in acute hippocampal slices and in the living cortex at 1 dpi. The level of motility impairment observed in the cortex in vivo was higher than in the acute hippocampal slices, possibly because the released ATP was washed out during the slice preparation. Our results of motility impairment are to some extent in disagreement with a recent study in acute hippocampal slices, in which they observed no difference in the processes velocity but a puzzling increase in the area of the explored territory of each process [61], in spite of the increased purinergic signaling of microglia in this model [58] and the well-established chemoattractant role of ATP in these lesions [4]. We speculate that at 2 dpi the widespread release of ATP induced by the seizures would be more attenuated than at 1 dpi, accounting for the dissimilar results on microglial motility.

In addition to the defect in motility, we have also found a defect in the apoptotic cell recognition and phagocytosis initiation. Indeed, a large proportion of apoptotic cells were located directly in apposition to microglial processes, and microglia down-regulated the expression of several phagocytic receptors, such as TREM2, CR3, MerTK, and GPR34. Therefore, the seizure-induced phagocytosis impairment is a complex phenomenon that likely implies other mechanisms in addition to the ATP widespread release. For instance, seizures affect many other signaling molecules released by neurons that control microglial function, such as fractalkine [62,63] or endocannabinoids [64,65]. In addition, status epilepticus triggers an early energy depletion at the seizure focus followed by a series of metabolic alterations in the long term [66] that affect the mitochondrial function [67], on which phagocytosis heavily relies, at least in macrophages [60]. Microglial motility and injury response also depends on energy sources, as these functions are early reduced in PM mouse tissue [24]. Therefore, some of these mechanisms are likely to play additional roles in regulating microglial phagocytosis efficiency at different stages of the disease.

### Long Term Impairment of Microglial Phagocytosis in Mouse and Human MTLE

The early phagocytosis impairment in the mouse model of MTLE was maintained in the long term, although there was a trend towards recovery of phagocytosis. In the subacute phase (3–7 dpi), microglia showed ameboid morphology and became multinucleated. This unresolved nucleokinesis was likely related to KA-induced inflammation, known to induce this phenotype in microglia in vitro [68]. Further, we also observed another inflammation-related phenotype: phagoptosis or the engulfment of nonapoptotic, seemingly live cells [17], albeit it occurred at lower levels than phagocytosis of apoptotic cells. In the chronic phase of MTLE (4 mpi), microglial phagocytosis continued to be impaired. Importantly, we obtained similar parameters (Ph index, distance to apoptotic cells, microglial density, microglial tissue volume) in hippocampal tissue from KA-treated mice and from MTLE patients. In the chronic phase of the mouse and human disease, the Ph index indicated that less than half of the apoptotic cells were being degraded by microglia, strongly suggesting that the microglial phagocytic impairment does occur in human MTLE. We have confirmed the microglial phagocytic impairment induced by seizures by comparing autopsy hippocampal tissue from age- and sex-matched nondemented controls and people diagnosed with epilepsy, although the PM delay severely interfered with the estimation of phagocytosis in this kind of tissue. In the human MTLE hippocampus, we have also discovered a novel form of phagocytosis (aster-type), executed by several ramified microglia with confluent processes towards the apoptotic cell, reminiscent of the microglial response to a laser-induced photo lesion observed by 2-photon imaging in the live mouse cortex [4]. While the functional relevance of the aster-phagocytosis and targeting of live neurons remains to be determined, the cellular mechanism of cell clearance in the epileptic mouse and human brain are remarkably similar.

### Detrimental Consequences of Microglial Phagocytic Impairment

We speculate that the early microglial phagocytosis impairment has detrimental consequences on the hippocampal function. Indeed, we have shown that the increased number of 3 do apoptotic cells found in the SGZ neurogenic niche after KA injection was not due to de novo apoptosis of this population induced by KA but instead, to an accumulation of 3 do newborn cells that undergo apoptosis under physiological conditions and are not phagocytosed by the impaired microglia. The nonphagocytosed apoptotic cells would evolve into secondary necrotic cells that lose membrane integrity and start leaking out intracellular contents [69], contributing to damaging the surrounding tissue. Recent data provides direct evidence of the beneficial effects of microglial phagocytosis, because transgenic silencing of the phagocytosis receptor TREM2 impairs microglial phagocytosis in vitro and exacerbates ischemic damage in experimental stroke [70]. In addition, we found that the impairment of phagocytosis could predict a large percentage (over 50%) of the variation in the levels of apoptosis, demonstrating that the efficiency of phagocytosis determines apoptosis dynamics in epilepsy and possibly in other brain diseases as well.

Finally, a strong body of in vitro data on macrophages and microglia has shown that phagocytosis of apoptotic cells is actively anti-inflammatory (reviewed in [2]). As we expected, impairment of microglial phagocytosis correlated with the expression of pro- and anti-inflammatory cytokines in hippocampal tissue. Inflammation per se did not impair phagocytosis, and thus this data suggests that the impairment of phagocytosis releases the brake on inflammation. Importantly, the same microglial population that exhibited impaired phagocytosis also had a strong proinflammatory profile. Increased expression of proinflammatory cytokines has already been observed in experimental and human MTLE tissue [71–73]. Because proinflammatory IL-1β enhances NMDA excitatory currents, it is speculated to contribute to development of chronic seizures [74], and drugs designed to prevent IL-1β activation and signaling are currently in clinical trials to prevent epileptogenesis [75]. Our data strongly support that this inflammation may at least in part originate from the microglial phagocytic impairment, but whether this dysfunction contributes to seizures remains to be determined. In summary, our results demonstrate that in the epileptic brain, microglia are not merely “reactive” to the neuronal damage but have their basal phagocytic function impaired. Because neuronal death and inflammation are hallmarks of all major brain diseases, such as ischemic stroke, Alzheimer, Parkinson, or multiple sclerosis, harnessing microglial phagocytosis may serve to control tissue damage and inflammation as a novel strategy to accelerate brain recovery.

## Materials and Methods

### Animals

All experiments were performed in fms-EGFP (MacGreen) mice, except the analysis of tissue cytokines by RTqPCR and the analysis of microglial motility, which were performed in C56BL/6 (Harlan, Boxmeer, the Netherlands) and CX3CR1^GFP/+^ mice, respectively. In both fms-EGFP [10,76] and CX3CR1^GFP/+^ mice [77], all microglia express the fluorescent reporter. Analysis of phagocytosis by nonprofesional phagocytes was done in POMC-EGFP [16], hGFAP-GFP [78], and nestin-GFP [79,80]. Analysis of the effect of infiltrating monocytes was carried out in CCR2^-/-^ mice [20]. All mice used were in a C57BL/6 background, except experiments with fatty acid diets, which were performed in CD-1 Swiss mice. Omega 3 deficient (containing 6% fat in the form of sunflower oil, rich in linoleic acid) or omega 3 balanced (containing a mixture of different oils rich in alpha-linolenic acid) diets were given immediately after mating and through gestation and lactation [11]; both diets were isocaloric and only their lipid composition was different (**S2 Table**). Mice were housed in 12:12h light cycle with ad libitum access to food and water. All procedures followed the European Directive 2010/63/EU, NIH guidelines, and Canadian Council on Animal Care guidelines, and were approved by the Ethics Committees of the University of the Basque Country EHU/UPV (Leioa, Spain; CEBA/205/2011, CEBA/206/2011, CEIAB/82/2011, CEIAB/105/2012), Bordeaux University (protocol number 5012094-A), and Southampton University (in accordance with United Kingdom Home Office licensing; project license 30/3056); the Baylor College of Medicine Institutional Animal Care and Use Committee (Houston, TX, US; AN- 5004); and the Animal Care Committee of Université Laval (protocol number 2013102–1). Unless otherwise stated, mice were 8 weeks old at the time of the KA injection.

### Intrahippocampal Injections

Induction of epilepsy was achieved by intrahippocampal injection of KA (Sigma-Aldrich, St Louis, MO, US) [81]. In brief, mice were anesthetized with ketamine/xylazine (10/1 mg/kg) and received a single dose of the analgesic buprenorphine (1 mg/kg) subcutaneously. After positioning in the stereotaxic apparatus, a 0.6 mm whole was drilled at coordinates taken from Bregma: AP −1.7 mm, laterolateral (LL) −1.6 mm. A pooled glass microcapillary was inserted at −1.9 mm dorsoventral (DV), and 50 nL of saline or KA (20 mM) were delivered into the right hippocampus using a microinjector (Nanoject II, Drummond Scientific, Broomal, PA, US). ATP (10, 100 mM; Sigma) and ATPγS (100 mM; Tocris) pH-balanced solutions were injected directly into the DG at coordinates AP −1.7 mm, LL −1.4 mm, DV −2.3 mm. After 2 min, the microcapillary was retracted, and the mice sutured and maintained in a thermal blanket until recovered from anesthesia. Some mice were implanted with platinum iridium, Teflon-coated deep electrodes (PlasticsOne, Roanoke, VA, US) immediately after intrahippocampal injection. Four recording electrodes were positioned at −1.6 mm AP, +1.8 mm LL, −1.8 mm DV (left hippocampus); −1.6 mm AP, −1.8 mm LL, −1.8 mm DV (right hippocampus); −0.1 mm AP, −1.8 mm LL, −2 mm DV (right cortex): −0.1 mm LL, +1.8 mm LL, −2 mm DV (left cortex). The reference electrode was placed at the frontal lobe at +0.1 mm AP, +0.1 mm LL, −0.5 mm DV, and the ground electrode was positioned over the cervical paraspinous area [13]. Four hours after implantation, every two days for the next week, and once a week for another 7 weeks, mice were attached to a Nicolet video-electroencephalogram (vEEG) system (NicView 5.71, CareFusion, San Diego, CA, US), and were recorded in 4 h sessions.

### Primary Microglia Cultures

Primary microglia cultures were performed as previously described [82]. Briefly, P0-P1 fms-EGFP mice pup brains were carefully peeled off meninges in Hank's balanced salt solution (HBSS, Hyclone) under a magnifying scope, and enzymatically digested with papain (20 U/ml, Sigma) and DNAse (150 U/μl, Invitrogen) for 15 min at 37ºC. The homogenization process was also helped by carefully pipetting. The resulting cell suspension was then filtered through a 40 μm nylon cell strainer (Fisher) and transferred to a 50 ml Falcon tube quenched by 5 ml of 20% heat inactivated Fetal Bovine Serum (FBS; Gibco) in HBSS. Afterwards, the cell suspension was centrifuged at 200 g for 5 min, the pellet was resuspended in 1ml Dulbecco’s Modified Eagle’s Medium/F12 (DMEM/F12, Gibco) complemented with 10% FBS and 1% Penicillin-Streptomycin (Gibco), and seeded in Poly-L-Lysine-coated (15 μl/ml, Sigma) culture flasks with a density of two brains per flask. Medium was changed every 3–4 d and enriched with granulocyte-macrophage colony stimulating factor (5 ng/ml GM-CSF, Sigma). After confluence (at 37°C, 5% CO_2_ for approximately 14 d), microglia cells were harvested by shaking at 100 rpm, 37°C, 4 h. Isolated cells were counted and seeded in a density of 80.000 cell/well on poly-l-lysine-coated 24-well plates. Microglia were allowed to rest and settle for at least 24 h before phagocytosis experiments. Primary microglia cells were fed for 3 h with NE-4C (American Type Culture Collection), a mouse neural stem cell line derived from the cortex of 9 do p53 knock-out embryos. NE-4C were previously labeled with the membrane marker CM-DiI (5 μM; 10 min at 37^°^C, 15 min at 4^°^C; Invitrogen) and treated with staurosporine (10 μM, 4h; Sigma) to induce apoptosis. This treatment resulted in 27.4% ± 8.5% of apoptotic and 1.2% ± 0.9% of necrotic NE-4C cells, as determined by flow cytometry analysis with Annexin V and PI; and in 35.0% ± 6.1% of apoptotic cells with pyknotic/karyorrhectic nuclear morphology determined with DAPI in immunofluorescent assays. Apoptotic NE-4C cells were added to the microglial cultures in a proportion 10:1 approximately. For the KA treatment, microglia were pretreated with 1 mM KA (Sigma) for 2 h before adding the apoptotic cells and remained present for the 3 h of the phagocytosis assay, accounting for a total of 5 h of KA treatment [48].

### Organotypic Hippocampal Slice Cultures

Organotypic hippocampal slice cultures were prepared as described previously [83] with minor modifications. In brief, 7 do fms-EGFP pups were decapitated and the brains extracted and placed in cold HBSS. Both hippocampi were dissected and cut into 350 μm slices using a tissue chopper (McIlwain). Slices were then transferred to 0.4 μm culture plate inserts (Millipore, PICM0RG50), each containing four slices. These membranes were placed in six-well plates, each well containing 1 ml of fresh organotypic culture medium. The medium consisted of 50% Neurobasal medium supplemented with 0.5% B27, 25% horse serum, 1% Glutamax, 1% penicillin/streptomycin, and 1% glucose solution in HBSS. Culture medium was changed the first day after doing the culture and every 2 d afterwards. Slices were kept in culture for 7 d before performing the experiments. For induction of excitotoxicity, hippocampal slices were treated with media containing 50 μM NMDA at day in vitro 7 for 4 h; another batch of slices were then placed in fresh culture medium for another 24 h. For KA experiments, hippocampal slices were treated with media containing 1 mM KA (Sigma) for 6 h. For ATP experiments, hippocampal slices were treated with media containing 300 μM and 1 mM ATP (Sigma) for 4 h. For experiments with the epileptogenic cocktail, hippocampal slices were treated for 1 h with either vehicle (oxygenated (95% O_2_, 5% CO_2_) ACSF, pH 7.4, containing 124 mM NaCl, 25 mM NaHCO_3_, 1.25 mM NaH_2_PO_4_, 2.5 mM KCl, 2.5 mM CaCl_2_, 1.3 mM MgCl_2_, and 10 mM D-glucose) or proepileptogenic cocktail (ACSF, with high K^+^ (8 mM), low Mg^2+^ (0.25 mM) and 4-AP (100 μM)) [84]. PI (5 μg/ml, Sigma) was added to the cultures in the last hour of the treatment.

### Human Samples from Individuals with MTLE

Freshly resected hippocampi from adult drug-resistant MTLE patients were obtained from the Basque Biobank at the Cruces University Hospital (Bilbao, Spain) with the patient’s written consent and with approval of the University of the Basque Country Ethics committee (CEISH/154/2012). The patient´s anonymity was preserved for this study. Immediately after surgery, the tissue was immersed in saline and maintained refrigerated until transported to the Pathology Unit of the Hospital (under 40 min), where it was manually sectioned in 1–2 mm thick coronal sections and transferred to 4% paraformaldehyde (PFA) in PBS, pH 7.4 for 30 min, then washed in PBS and kept in cryoprotectant (30% sucrose, 30% ethyleneglycol in PBS) at −20°C. Parafin-embedded hippocampal tissue from epileptic patients and nondemented controls was obtained from The Netherlands Brain Bank, Netherlands Institute for Neuroscience, Amsterdam (open access www.brainbank.nl). All material was collected from donors for or from whom a written informed consent for a brain autopsy and the use of the material and clinical information for research purposes had been obtained by the NBB.

### Immunofluorescence

Mice were transcardially perfused with 30 ml of PBS followed by 30 ml of 4% PFA. The brains were removed and postfixed with the same fixative for 3 h at room temperature, then washed in PBS and kept in cryoprotectant at −20°C. Six series of 50 μm-thick sections of mouse (sagital) or human (coronal) brains were cut using a Leica VT 1200S vibrating blade microtome (Leica Microsystems GmbH, Wetzlar, Germany). Fluorescent Immunostaining was carried out following standard procedures [9]. Free-floating vibratome sections or organotypic slices were incubated in permeabilization solution (0.3% Triton-X100, 0.5% BSA in PBS; all from Sigma) containing 5% NGS for 1 hr at room temperature, and then incubated overnight with the primary antibodies diluted in the permeabilization solution at 4°C. For BrdU staining, sections were pretreated with 2M HCl for 15 min at 37°C and washed with 0.1 M sodium tetraborate for 10 min at RT prior to staining with the primary antibodies. After thorough washing with PBS, the sections were incubated with fluorochrome-conjugated secondary antibodies and DAPI (5 mg/ml; Sigma) diluted in the permeabilization solution for 2 hr at room temperature. Primary microglial cultures were fixed for 10 min and organotypic slices for 40 min and then transferred to PBS. Coverslips with primary microglial cultures were blocked in 1% normal goat serum (NGS, Sigma), 0.2% Triton X-100 in PBS for 30 min. The cells were then incubated with primary antibodies in 0.2% Triton X-100 PBS for 1 h at RT, washed in PBS and incubated in the secondary antibodies containing DAPI (5 mg/ml) in the same solution for 1 h at RT. After washing with PBS, the sections, organotypic cultures, and primary cultures were mounted on glass slides with DakoCytomation Fluorescent Mounting Medium (DakoCytomation, Carpinteria, CA). The following antibodies were used: chicken anti-GFP (1:750; Aves Laboratories, Tigard, OR); mouse anti-NeuN (1:1,000; EMD Millipore Corporation, Billerica, MA, US); mouse anti-CD45.2 (1:100; BD Pharmingen, Spain); rabbit antiactivated-caspase-3 (1:100; Cell Signaling Technology, Danvers, MA); rabbit anti-cfos (1:1,000; Santa Cruz Biotechnologies, Heidelberg, Germany); rabbit anti-Ki67 (1:1,000; Vector Laboratories, Burlingame, CA, USA); rabbit anti-Iba1 (1:1,000; Wako Chemicals, GMBH, Germany); rabbit anti-phosphoHistone 3 (1:1,000; Millipore); rat anti-BrdU (1:300; AbD Serotech, Kidlington, UK). Secondary antibodies coupled to AlexaFluor 488, Rodhamine Red X, or AlexaFluor 647 were purchased from Molecular Probes (Willow Creek Road, Eugene, O) or from Jackson Immunoresearch (West Grove, PA).

### Image Analysis

All fluorescence immunostaining images were collected using an Olympus Fluoview or a Leica SP8 laser scanning microscope using a 40X oil-immersion objective and a z-step of 0.7 μm. All images were imported into Adobe Photoshop 7.0 (Adobe Systems Incorporated, San Jose, CA) in tiff format. Brightness, contrast, and background were adjusted equally for the entire image using the “brightness and contrast” and “levels” controls from the “image/adjustment” set of options without any further modification. 3D-rendering of phagocytic cells was performed using ImageSurfer (NIH) or ImageJ (Fiji distribution). Quantitative analysis of apoptosis and phagocytosis was performed using unbiased stereology methods as previously described [9]. For mouse tissue sections, 2–3 20 μm-thick z-stacks located at random positions containing the DG were collected per hippocampal section, and a minimum of 6 sections per series were analyzed. For human tissue, 2–3 coronal sections were fully scanned under the microscope to find all apoptotic cells. For organotypic cultures, 3 20 μm-thick random z-stacks of the DG were collected per hippocampal slice, using a 60X oil-immersion objective. For primary cultures, over 10 random z-stacks were obtained per coverslip.

### Phagocytosis Analysis

Apoptotic cells were defined based on their nuclear morphology after DAPI staining as cells in which the chromatin structure (euchromatin and heterochromatin) was lost and appeared condensed and/or fragmented (pyknosis/karyorrhexis); they also colocalized with activated-caspase-3, a well-known marker of apoptosis. Phagocytosis was defined as the formation of an enclosed, three-dimensional pouch of microglial processes surrounding an apoptotic cell. In tissue sections and organotypic cultures, the number of apoptotic cells, phagocytosed cells, BrdU^+^ cells, and microglia were estimated in the volume of the DG contained in the z-stack (determined by multiplying the thickness of the stack by the area of the DG at the center of the stack using ImageJ (Fiji)). To obtain the absolute numbers (in tissue sections), this density value was then multiplied by the volume of the septal hippocampus (spanning from −1 mm to −2.5 mm in the AP axes, from Bregma; approximately six slices in each of the six series), which was calculated using Fiji from a Zeiss Axiovert epifluorescent microscope images collected at 20X. In organotypic cultures, the number of apoptotic cells and microglia in the DG was given as a density, over a 200.000 μm^3^ volume (roughly, a 100 x 100 μm^2^ area of 20 μm of thickness). In primary cultures, the percentage of phagocytic microglia was defined as cells with pouches containing NE-4C nuclei and/or CM-DiI particles. The following formulae were used to estimate microglial phagocytic efficiency in tissue or organotypic cultures:

$$\text{Ph index}=\frac{{apo}^{Ph}}{{apo}^{tot}}$$

$$\text{Ph capacity}=\frac{{{mg}^{Ph1}+2{mg}^{Ph2}+3{mg}^{Ph3}\ldots +nmg}^{Phn}}{mg}$$

$$\text{Ph}/\text{A coupling}=\frac{\mathrm{Ph capacity x microglia}}{{apo}^{tot}}$$

$$\text{Clearance time}=\frac{{apo}^{tot}(t2)x\;\Delta t}{\Delta BrdU}$$

where apo^Ph^ is the number of apoptotic cells phagocytosed; apo^tot^ is the total number of apoptotic cells; and mg^Phn^ is the proportion of microglia with n phagocytic pouches.

The percentage of volume occupied by Iba1^+^ microglia was estimated in confocal z-stacks of the DG and hilus. First, microglia were manually selected in each z-stack using the “Threshold” tool (Fiji) to mask only the pixels of the image with Iba1^+^ staining. Then, the percentage of pixels occupied by microglia was calculated using the Area Fraction parameter of the “Measure” tool (Fiji). All commands were automated in an ImageJ macro (Fiji). The average Area Fraction from a minimum of ten images per z-stack was calculated. For mouse tissue, 2–3 20 μm-thick z-stacks containing the DG and hilus were collected per hippocampal section and a minimum of six sections per series were analyzed. For human tissue, 3–4 12 μm-thick z-stacks located at random positions containing the DG and hilus were analyzed.

### Two-Photon Imaging on Acute Hippocampal Slices

Brain slices were obtained from CX3CR1^GFP/+^ mice aged 2 mo 1 dpi or 7 dpi of KA or saline. As we previously described [32], animals were quickly anesthetized with isoflurane, and 300 μm-thick coronal slices were made using a Vibratome (VT1000S, Leica, Nanterre, France). Slices were then stored at room temperature (20 to 23°C) for one hour before imaging in an oxygenated artificial cerebrospinal fluid (ACSF) containing 126 mM NaCl, 2.0 mM CaCl_2_, 2.0 mM MgCl_2_, 2.5 mM KCl, 1.25 mM NaH_2_PO_4_, 26 mM NaHCO_3_, 10 mM glucose, 1 mM ascorbic acid, 4 mM sodium pyruvate, and saturated with 95% O_2_ and 5% CO_2_ (310 ± 5 mOsm). Slices were transferred to a recording chamber and perfused with oxygenated aCSF at a rate of 1–3 ml/min and maintained at 25°C with an inline heater. Two-photon imaging was performed with a laser-scanning microscope Leica DMLFSA TCS SP2 on an upright stand (Leica Microsystems, Mannheim, Germany) coupled to a femtosecond pulsed Ti:Sapphire laser (Mira 900, Coherent Laser Group, Santa Clara, CA, US). The laser was tuned to the excitation wavelength for GFP (900 nm), and there was no photobleaching nor was there any evidence of cellular damage during extensive scanning to obtain time lapse images. The laser intensity was carefully monitored in all instances and kept comparable between all experiments. A HCX IR Apo L 25X NA 0.95 (Olympus) water-immersion objective lens was used. Imaging was done at depths in brain slices >50 μm and up to 100 μm. The mean depth for imaging lesions was 75 μm. Voxel size was adjusted to 0.1 x 0.1 μm, and z-stacks were taken in 1 μm steps. The mean scan time for z-stack was approximately 45 s. 3-D reconstruction of microglia and automated assessment of the number of branches was performed using the “filament tracer program” (Matlab algorithm) of Imaris 7.6 (Bitplane AG), after correction for drift in *x*- and *y*-axis (Stackreg [85] and Multistackreg [86] plugins, Fiji) and drift in z-axis ("correct 3-D axis", Fiji module of Imaris 7.6). This allowed us to isolate each microglial process and to follow its length modification all along the recording period. In both saline- and KA-injected animals, we analyzed 4–5 cells (distributed through the hilus and the DG) per animal and 3–4 animals per group.

### Two-Photon Imaging on the Living Cortex

Live imaging was performed using two-photon imaging as previously described [33]. 2 mo CX3CR1^GFP/+^ mice were injected with saline or KA as above. 24 h later, mice were anesthetized with isoflurane. The skull above the motor cortex was exposed, cleaned, glued to a thin metal plate, and carefully thinned to an approximately 20- to 30-mm thickness, using a high-speed dental drill (Osada Inc) and a microsurgical blade. Drilling was interrupted periodically, and sterile saline was applied on the skull to prevent heat-induced damage. Next, the mice were placed under an Olympus two-photon microscope FV1000MPE equipped with a Ti:Sapphire laser (Mai Tai DeepSee; Spectra Physics) tuned to 920 nm for transcranial imaging. A 25X water-immersion lens (1.05 N.A.; Olympus) was used throughout the imaging session. Z stacks taken 1 μm apart were acquired every 1.5 min for 13.5 min (ten time frames). Microglial motility was analyzed using several plugins in Fiji. In brief, images were registered using the “Affine” algorithm of the “MultiStack” plugin and aligned by using the “Correct 3D drift” plugin [87]. Background was subtracted using a difference of Gaussians and bleaching was corrected using the “Histogram Matching” algorithm of the “Bleach Correction” plugin [88]. The motility was automatically determined using a self-developed ImageJ macro that estimated the 3-D length of previously selected processes and calculated the motility as the absolute difference of length between two consecutive frames divided by the time interval (1.5 min). For automatic measurement of the length process, each selected process was reoriented vertically in the xy plane and the intensity profiles of horizontal lines run through the length of the process were obtained in each z-slice.

Intensity profiles were used to determine the x and z coordinates of the borders of the process in each line based on three parameters: background intensity, the difference between the maximum intensity and the pixels flanking the maxima, and the inflexion points of the intensity profiles. x and z coordinates of the borders were used to calculate the center of the process in each horizontal plane (xz planes). Then, the length of the 3-D skeleton of the process in each time frame was calculated as the summation of the distances between each pair of center points located in consecutive xz planes. The following formulae were used to calculate the mean motility of a process:

$$z_{C}=\frac{z_{U}+z_{B}}{2}$$

Where z_C_, z_U_, and z_B_ are the coordinates of the center, the upper z-slice and the bottom z-slice containing the process, respectively.

$$x_{C}=\frac{x_{R}+x_{L}}{2}$$

Where x_C_, x_R_, and x_L_ are the coordinates of the center, the right border and the left border of the process, respectively. If z_c_-slice was virtual (i.e., not an integer):

$$x_{R}=\frac{x_{R_{\left(z_{c}-0.5\right)}}+\;x_{R_{\left(z_{c}+0.5\right)}}}{2}$$

$$x_{L}=\frac{x_{L_{\left(z_{c}-0.5\right)}}+\;x_{L_{\left(z_{c}+0.5\right)}}}{2}$$

Once the coordinates of the center of the vertically aligned process had been defined in the X and Z planes, its length was estimated:

$$\mathrm{length}=\sum_{i=y_{0}}^{y_{n-1}}\sqrt{{{\left(y_{i+1}-y_{i}\right)}^{2}+(x_{c_{y_{i+1}}}-x_{c_{y_{i}}})}^{2}+{\left(z_{c_{y_{i+1}}}-z_{c_{y_{i}}}\right)}^{2}}$$

Where y_0_ and y_n-1_ are the y coordinates of the first and last horizontal lines, respectively, containing pixels with intensities above the background. Finally, the process motility was estimated:

$$\mathrm{motility}=\frac{\sqrt{{\left({length}_{f+1}-\;{length}_{f}\right)}^{2}}}{1.5}$$

Where f is time frame, and 1.5 corresponds to the 1.5 min of the time interval between consecutive frames. Mean motilities were used for analysis. Mean protraction and retraction of a process were calculated as the mean of the motilities from consecutive frames (f and f+1) where the length of the process was increased or decreased, respectively.

### Electrophysiology

Hippocampal slices (300 μm) from PND20-PND30 days-old fms-EGFP mice were prepared in oxygenated (95% O_2_,5% CO_2_) ACSF, pH 7.4, that contained 124 mM NaCl, 25 mM NaHCO_3_, 1.25 mM NaH_2_PO_4_, 2.5 mM KCl, 2.5 mM CaCl_2_, 1.3 mM MgCl_2_, and 10 mM D-glucose. Slices were allowed to recover for at least 1 h and were then transferred to a 37ºC chamber with continuous flow (1 mL/min) of oxygenated ACSF. To induce epileptiform activity, cells were perfused with high K^+^ (8 mM), low Mg^2+^ (0.25 mM) and 4-AP (100 μM) [84]. Extracellular field potential recording were performed in the CA1 pyramidal layer to monitor epileptiform activity, using glass electrodes (1 MΩ) filled with aCSF. Epileptiform activity was induced both in the DG and CA1, but the amplitude of the spikes was considerably larger in CA1 and thus EGFP-expressing microglial cells were simultaneously patch-clamped recorded in this region in whole-cell configuration with recording pipettes (7–10 MΩ) filled with a solution containing 135 mM KCl, 4 mM NaCl, 0.7 mM CaCl_2_, 10 mM BAPTA, 10 mM HEPES, 4 mM Mg-ATP and 0.5 mM Na_2_-GTP (pH 7.2).

### FACS Sorting

Microglia cells were isolated from brains as described previously [10]. The hippocampi and cortices from 2-mo-old fms-EGFP mice were dissected and placed in enzymatic solution (116 mM NaCl, 5.4 mM KCl, 26 mM NaHCO_3_, 1 mM NaH_2_PO_4_, 1.5 mM CaCl_2_, 1 mM MgSO_4_, 0.5 mM EDTA, 25 mM glucose, 1 mM L-cysteine) with papain (20 U/ml) and DNAse I (150 U/μl, Invitrogen) for digestion at 37ºC for 15 min. For glutamate receptor subunit expression experiments, 8 hippocampi, and 3 hemicortices from 4 mice were collected per replica, with a total of 4 replicas. For cytokine expression experiments, 4 hippocampi from saline or KA-injected mice were collected per replica, with a total of 4 replicas. After homogenization, tissue clogs were removed by filtering the cell suspension through a 40 μm nylon strainer to a 50 ml Falcon tube quenched by 5 ml of 20% heat inactivated FBS in HBSS. For further enrichment of microglia, myelin was removed by using Percoll gradients. For this purpose, cells were centrifuged at 200 g for 5 min and resuspended in a 20% Solution of Isotonic Percoll (20% SIP; in HBSS), obtained from a previous stock of SIP (9 parts Percoll per 1 part PBS 10X). Then, each sample was layered with HBSS poured very slowly by fire-polished pipettes. Afterwards, gradients were centrifuged for 20 min at 200 g with minimum acceleration and no brake so the interphase was not disrupted. Then the phase was removed, cells were washed in HBSS by centrifuging at 200 g for 5 min and pellet was resuspended in 500 μl of sorting buffer (25 mM HEPES, 5 mM EDTA, 1% BSA, in HBSS). Microglia cell sorting was performed by FACS Jazz (BD), in which the population of green fluorescent cells was selected, collected in Lysis Buffer (Qiagen) containing 0.7% β-mercaptoethanol and stored at −80ºC until processing.

### RNA Isolation and RTqPCR

The right hippocampus of wild type mice was rapidly isolated immediately after intraaortic perfusion with cold PBS under tribromoethanol overdose, and stored at −80°C until processed. Total RNA was isolated using a roto-stator homogenizer and Qiagen RNeasy Mini Kit (Alcobendas, Spain), following manufacturer’s instructions, including a DNAse treatment step. RNA was quantified in a Nanodrop 2000, and 1.5 μg were retrotranscribed using random hexamers (Invitrogen) and Superscript III Reverse Transcriptase kit (Invitrogen), following manufacturer’s instructions in a Veriti Thermal Cycler (Applied Biosystems, Alcobendas, Spain). RNA from FACS-sorted microglia was isolated by Rneasy Plus micro kit (Qiagen) according to the manufacturer instructions, and the RNA was retrotranscribed using an iScript Advanced cDNA Synthesis Kit (Biorad). qPCR was performed following MIQE guidelines (Minimal Information for Publication of Quantitative Real Time Experiments [89]). Three replicates of 1.5 μl of a 1:3 dilution of cDNA were amplified using Power SybrGreen (Biorad) for hippocampal experiments or SsoFast EvaGreen Supermix (Biorad) for FACS-sorted microglia experiments in a CFX96 Touch Real-Time PCR Detection System (Biorad). The amplification protocol for both enzymes was 3 min 95°C, and 40 cycles of 10 s at 95°C, 30 s at 60°C. Primers were designed to amplify exon–exon junctions using Primer Express (Applied Biosystems) or PrimerBlast (NIH) to avoid amplification of contaminating genomic DNA, and their specificity was assessed using melting curves and electrophoresis in 2% agarose gels. Primer sequences are listed in **S3 Table**. For each set of primers, the amplification efficiency was calculated using a standard curve of 1:2 consecutive dilutions, and was used to calculate the relative amount using the following formula:

ΔΔCt=(1+eff.targetgene)exp(Ctsample−Ctcontrol)/(1+eff.referencegene)exp(Ctsample−Ctcontrol)

Two independent reference genes were compared: L27A, which encodes a ribosomal protein of the 60S subunit [10] and OAZ-1, which encodes ornithine decarboxylase antizyme, a rate-limiting enzyme in the biosynthesis of polyamines and recently validated as reference gene in rat and human [90]. In all experiments, the pattern of mRNA expression was similar using both reference genes, and in each experiment the reference gene that rendered lower intragroup variability was used for statistical analysis.

### Statistical Analysis

SigmaPlot (San Jose, CA, USA) was used for statistical analysis. For the analysis of cytokine mRNA expression, a logarithmic transformation was performed to comply with ANOVA assumptions (normality and homocedasticity) [10]. The analysis of cytokine and apoptotic cell expression was evaluated by two-way ANOVA or the corresponding non-parametrical test (Kruskal-Wallis). When interaction between factors (time x treatment) was found, a 1-way ANOVA test of all groups was performed instead to determine the overall effect of each factor. In all cases, all-pairwise multiple comparisons (Holm-Sidak method or Tukey test) were used as a posthoc test to determine the significance between groups in each factor. Only *p* < 0.05 is reported to be significant. Data is shown as mean ± SEM (standard error of the mean).

The underlying data used in all figures are included in **S1 Data**.

## Supporting Information

S1 Data Excel files containing numerical values for all data shown in Figs 1–12 and S1–S13 Figs. (XLSX)

S2 Data Flow cytometry file containing CD45 data for control #1. (LMD)

S3 Data Flow cytometry file containing CD45 data for control #2. (LMD)

S4 Data Flow cytometry file containing CD45 data for control #3. (LMD)

S5 Data Flow cytometry file containing CD45 data for control #4. (LMD)

S6 Data Flow cytometry file containing CD45 data for KA 3 dpi #1. (LMD)

S7 Data Flow cytometry file containing CD45 data for KA 3 dpi #2. (LMD)

S8 Data Flow cytometry file containing CD45 data for KA 3 dpi #3. (LMD)

S9 Data Flow cytometry file containing CD45 data for KA 3 dpi #4. (LMD)

S10 Data Flow cytometry file containing CD45 data for KA 7 dpi #1. (LMD)

S11 Data Flow cytometry file containing CD45 data for KA 7 dpi #2. (LMD)

S12 Data Flow cytometry file containing CD45 data for KA 7 dpi #3. (LMD)

S13 Data Flow cytometry file containing CD45 data for KA 7 dpi #4. (LMD)

S1 Fig Phagocytosis in the PND developing hippocampus. (**A**) Representative confocal z-stack projections of the DG of the hippocampus at PND d 7 (PND7) and PND14 of fms-EGFP mice, the ages when organotypic cultures were cultured (PND7) and were used for experiments (PND7+7DIV). Apoptotic (pyknotic, white, DAPI; arrows) cells were phagocytosed by terminal or en passant branches of microglia (fms-EGFP^+^, cyan; M). Scale bars = 50 μm. z = 16.8 μm. (**B**) Ph index in the DG at PND7 and 14 (in % of apoptotic cells; *n* = 3–4 per group). Bars represent mean ± SEM. * indicates *p* < 0.05 and ** indicates *p* < 0.01 by one-tail Student´s *t* test. Underlying data is shown in **S1 Data**.
(TIF)

S2 Fig Effect of KA on apoptosis and phagocytosis in the DG, CA, and cortex. (**A**) Representative confocal z-stack projections of the CA1, CA3 regions of the hippocampus, and cortex (Cx) of 2 mo fms-EGFP mice injected with saline (left panels) or KA (right panels) at 1 dpi. Apoptotic cells (pyknotic, white, DAPI; arrowheads) are largely absent in control conditions but present in KA-treated mice in the three regions. Some apoptotic cells were phagocytosed (arrows) by microglia (fms-EGFP^+^, cyan) but most were not (arrowheads). Similar images of the DG are shown in **Fig 3B**. Scale bars = 50 μm. z = 18.2 μm (except in CA1 KA1 dpi = 20.3 μm, CA3 sal 1 dpi = 17.5 μm and Cx KA 1 dpi = 19.6 μm). (**B**) Density of apoptotic (pyknotic/karyorrhectic and act-casp3^+^) per mm^3^ (*n* = 3 per region and treatment). (**C**) Ph index (in % of apoptotic cells) in the different brain regions after KA. nd, not detected; na, not applicable. Bars represent mean ± SEM. ** indicates *p* < 0.01, and *** indicates *p* < 0.001 by Student’s *t* test. Underlying data is shown in **S1 Data**.
(TIF)

S3 Fig Early effects of KA in the mouse hippocampus. (**A**) Density of microglia (cells/mm^3^) in saline and KA-injected mice (*n* = 3–5 per group). At 1 dpi, KA induced a significant decrease in the density of microglia. (**B**) Volume of the septal DG (mm^3^) in saline and KA-injected mice (*n* = 3–5 per group). The volume occupied by the DG was assessed in the septal hippocampus (spanning from −1 mm to −2.5 mm in the AP axes, from Bregma) in control animals (injected with saline, pooled from different time points for robustness) or after injection of KA at 1, 3, and 7 dpi (no changes after 6 hpi were found). (**C**) Representative orthogonal projection (upper panel) and 3-D-rendered image (lower panel) of a confocal z-stack showing an apoptotic cell (pyknotic, white, DAPI) expressing activated-caspase 3 (act-casp3^+^, red) phagocytosed by a hGFAP^+^ astrocyte (green), nearby a microglial cell (Iba1^+^, cyan). (**D**) Representative orthogonal projection (upper panel) and 3-D-rendered image (lower panel) of a confocal z-stack showing an apoptotic cell (pyknotic, white, DAPI) expressing activated-caspase 3 (act-casp3^+^, red) phagocytosed by a POMC^+^ neuroblast (yellow), nearby a microglial cell (Iba1^+^, cyan). (**E**) Ph index of microglia, astrocytes and neuroblasts (in %) at 1 dpi after the injection of KA (*n* = 3–4 per group). At 1 dpi, the impaired microglia remained the major phagocytic cell in the hippocampus, as it engulfed a higher percentage of apoptotic cells. (**F**) Average distance (in μm) between apoptotic nuclei and the closest (perpendicular) microglial process. Apoptotic cells were analyzed from 3 animals per group (*n* = 6, 73, and 189 cells for control, KA 6 hpi and KA 1 dpi, respectively). The distance increased significantly by 1 dpi after KA. Bars represent mean ± SEM. In A, ** indicates *p* < 0.01 by Holm-Sidak posthoc test after two-way ANOVA was significant at *p* < 0.05. In B, a indicates *p* < 0.05 versus control, b indicates *p* < 0.05 versus KA 7 dpi by Holm-Sidak posthoc test after one-way ANOVA was significant at *p* < 0.05. In F, *** indicates *p* < 0.001 by Dunn´s posthoc test after Kruskal-Wallis test was significant at *p* < 0.05. Scale bars = 10 μm. z = 11.9 μm. Arrows, phagocytic pouches. Underlying data is shown in **S1 Data**.
(TIF)

S4 Fig Long term effects of KA in the mouse hippocampus. (**A**) Representative projection of a confocal z-stack showing several large multinucleated phagoptotic microglia (fms-EGFP^+^, cyan) from the hippocampus of a KA mouse (3 dpi). (**A1**) 3-D-rendered image showing the continuum of EGFP through the microglial cytoplasm and within their nuclei. Up to five nuclei were contained. (**A2**) Panel showing each nucleus individually. Nucleus 5 was small but not pyknotic and was surrounded by a pouch of microglial cytoplasm. (**A3**) Orthogonal projections of nucleus 5 showing its complete engulfment by microglial processes (phagoptosis). Arrowhead point towards a nonengulfed apoptotic cell. (**B**) Gating strategy used for the analysis of CD45 expression in microglia from control and KA-injected mice (3 and 7 dpi). First, debris was excluded using the R1 gate in FSC versus SSC. Next, fms-EGFP^+^ cells were gated in R2 in EGFP vs FSC. A small population of microglial processes was excluded from R2 [10]. CD45^low^ cells are shown in cyan and CD45^high^ in red (as in **Fig 5**). Original FSC files can be found in **S2–S5 Data** (control), **S6–S9 Data** (KA 3 dpi), and **S10–S13 Data** (KA 7 dpi). (**C**) Septal DG volume (in mm^3^) in WT and CCR2 KO mice at 3 dpi. (**D**) Representative projection of a confocal z-stack showing neutrophils in the spleen challenged with LPS (8 h), stained with myeloperoxidase (MPO, red) in fms-EGFP+ cells. (**E**) Representative projection of a confocal z-stack showing neutrophils in the DG of a CCR2 KO mice injected with KA at 3 dpi. Neutrophils expressed CD11b (green) and MPO (red). (**F**) Septal DG volume (in mm^3^) in saline and KA-injected mice at 4 mpi. (**G**) Number of apoptotic cells per septal hippocampus in saline (*n* = 7) and KA (*n* = 8) mice at 4 mpi after the injection of KA. (**H**) Representative projection of a confocal z-stack showing phagocytosis by a reactive astrocyte in the hippocampus of a KA mice (4 mpi). Arrows point towards the phagocytosed apoptotic cell. rA, reactive astrocyte; M, microglia. Scale bars = 10 μm (A, G), 20 μm (C, D). z = 17 μm (A), 9,8 μm (C, D), 7.2 μm (C). Bars represent mean ± SEM, *** indicates *p* > 0.001 by Student’s *t* test. Underlying data is shown in **S1 Data**.
(TIF)

S5 Fig Microglial proliferation along the time course after KA. (**A**) Representative confocal z-stack projection of uni- (left) and multinucleated (right) microglia (fms-EGFP^+^, cyan) in the DG 3 dpi after KA. Proliferating cells were labeled with Ki67 (red) and nuclei with DAPI (white). Scale bars = 10μm. z = 9.8 μm (**B**) Percentage of uni- (grey) and multinucleated (white) microglia labeled with Ki67 from 6hpi to 7 dpi. Bars represent mean ± SEM. nd, not detectable. a indicates *p* < 0.05 between uninucleated microglia at 3 and 7 dpi, bbb indicates *p* < 0.001 between multinucleated microglia at 3 and 7 dpi by Student´s *t* test. Underlying data is shown in **S1 Data**.
(TIF)

S6 Fig Expression of CD11b in microglia along the time course after KA. (**A**) Representative confocal z-stack projections of the hippocampus in control (not injected) and KA-injected mice from 6 hpi to 7 dpi. Nuclei are labeled with DAPI (white), and microglia with fms-EGFP (cyan) and CD11b (magenta). The expression of CD11b increased over the time course. Scale bars = 500 μm. z = 7.7 μm (control, KA 6 hpi), 9.1 μm (KA 1 dpi), 9.8 μm (KA 3 dpi, 7 dpi). (**B**) High magnification inserts of the DG and CA1 region in KA mice at 7 dpi. Scale bars = 100 μm. z = 14.7 μm (DG), 11.9 μm (CA1).
(TIF)

S7 Fig Expression of CD68 in microglia along the time course after KA. (**A**) Representative confocal z-stack projections of the hippocampus in control (not injected) and KA-injected mice from 6 hpi to 7 dpi. Nuclei are labeled with DAPI (white), and microglia with fms-EGFP (cyan) and CD68 (magenta). The expression of CD68 increased over the time course. Scale bars = 500 μm. z = 9.8 μm (control, KA 7 dpi), 8.4 μm (KA 6 hpi), 10.5 μm (KA 1 dpi, 3 dpi). (**B**) High magnification inserts of the DG and CA1 region in KA mice at 7 dpi. Scale bars = 100 μm. z = 12.6 μm (DG), 11.9 μm (CA1).
(TIF)

S8 Fig Microglial phagocytosis in human epilepsy. (**A**). Representative confocal z-stack projection of two neurons in the hilus (NeuN^+^, magenta; arrows) surrounded by a mesh of microglial processes (Iba1^+^) in the hippocampus of biopsy tissue obtained from an MTLE patient. Nuclei are shown in white (DAPI). The right panel shows an orthogonal projection of the same cells (N1 and N2). (**B**) Representative confocal z-stack projections of apoptotic cells (pyknotic, DAPI, white) not phagocytosed (B1, in the granular layer) and phagocytosed (B2, in the hilus) by microglia (Iba1^+^, cyan) in the hippocampus of autopsy tissue from a nondemented control. (**C**) Representative confocal z-stack projections of apoptotic cells (pyknotic, DAPI, white) not phagocytosed (C1, in the hilus) and phagocytosed (C2, in the granular layer) by microglia (Iba1^+^, cyan) in the hippocampus of autopsy tissue from an epileptic patient. The number of engulfed apoptotic cells evaluated is shown in **S1 Table**. Scale bars = 50 μm (A), 20 μm (B, C). z = 15.7 μm (A), 3.5 μm (B1), 8.05 μm (B2), 4.55 μm (C1), 9.1 μm (C2).
(TIF)

S9 Fig RTqPCR analysis of glutamate receptor subunit expression in microglia. Microglia was FACS-sorted from the hippocampus and the cortex of 2 mo mice, and their expression of ionotropic and metabotropic glutamate receptor subunit assessed by RTqPCR. Two PND8 hippocampi were used as positive control (except for Grm6, where the retina of a 2 mo mouse was used), and the RT- as negative control. (**A**) Amplification plots for each subunit showing the cycle versus the RFU (relative fluorescent units). The threshold level of fluorescence used to determine the threshold cycle is shown as a straight dark green line. Microglia had a low, but consistent expression of all subunits above the threshold and clearly different from the RT-. (**B**) Denaturing curves for each subunit showing the increase in temperature versus the decrement in fluorescence. All primers used were checked against forming primer dimer or other nonspecific products.
(TIF)

S10 Fig Seizures, but not KA, impair phagocytosis in organotypic slices. (**A**) Number of apoptotic cells in a 200.000 μm^3^ volume in organotypic slices treated with KA (1 mM, 6 h). No significant differences were found between KA (*n* = 5) and control (vehicle; *n* = 3) slices, although there was a tendency to found fewer apoptotic cells in KA-treated slices (*p* = 0.08). (**B**) Weighted Ph capacity (in ppu) in organotypic slices treated with KA. (**C**) Number of microglia within the slice in a 200.000 μm^3^ volume in organotypic slices treated with KA. (**D**) Experimental design and representative images of the DG of hippocampal organotypic slices treated with ACSF (control) or an epileptogenic cocktail (high K^+^, low Mg^2+^, 4-AP) for 1 h. Normal or apoptotic (pyknotic/karyorrhectic) nuclear morphology was visualized with DAPI (white), microglia by the transgenic expression of fms-EGFP (cyan), and membrane permeability (characteristic of necrotic cells) by PI (red). High magnification inserts show details of phagocytosed apoptotic cells in the two conditions. Arrows, phagocytosed cells; arrowheads, non-phagocytosed cell. Scale bars = 30 μm. (**E**) Number of dead apoptotic cells in 200.000 μm^3^ of the DG in organotypic slices treated with the epileptogenic cocktail. (**F**) Ph index in organotypic slices (% of apoptotic cells phagocytosed) treated with the epileptogenic cocktail. Note that the Ph index in ACSF-treated slices is higher than in organotypic culture media-treated slices (**Fig 1**). (**G**) Weighted Ph capacity of microglia (in parts per unit, ppu). (**H**) Histogram showing the Ph capacity of microglia (in % of cells). (**I**) Number of microglial cells. (**J**) Ph/A coupling (in fold-change) in organotypic slices treated with the epileptogenic cocktail. (**K**) Extracellular recording of the seizure activity induced by the epileptogenic cocktail before and after the purinergic antagonist BBG was added in acute hippocampal slices. The effect of this drug in microglial currents is shown in **Fig 9A–9C**. (**L**) Spike amplitude (in mV) induced by the epileptogenic cocktail. (**M**) Spike frequency (in Hz) induced by the epileptogenic cocktail. Bars represent mean ± SEM. * indicates *p* < 0.05 and ** *p* < 0.01 by Student´s *t* test. Underlying data is shown in **S1 Data**.
(TIF)

S11 Fig Effect of ATP in vivo. (**A**) Tiled confocal z-stack of the injection site in ATP-injected mice (100 mM, 2h). Note that the injected volume is larger than in the KA injections (1 μl versus 50 nL) and thus the tissue damage is more apparent. Nuclei are shown with DAPI in white and microglia is visualized with fms-EGFP in cyan. Inserts show details of the cortex (**A1**, **A2**), CA3 (**A3**), DG (**A4**), and CA1 (**A5**). The effect of injected ATP was restricted to the DG, as determined by the change in microglial morphology. (**B**) Representative confocal z-stack of the DG in mice injected with vehicle (control) or ATP (10 or 100 mM) at 4 hpi. (**C**) Septal DG volume (in mm^3^) in saline and ATP-injected mice at 4 hpi. Scale bars = 500 μm (A, tiled image), 100 μm (A, details), 50μm (B). z = 25.2 μm (A, tiled image), 16.8 μm (A, details), 14 μm (B). Underlying data is shown in **S1 Data**.
(TIF)

S12 Fig Expression of pannexin in the DG along the KA time course. Representative confocal z-stack projections of the DG in control (1 and 2 mo) and KA-injected mice from 6 hpi to 7 dpi showing the low expression of pannexin (magenta) in granule neurons in the DG. Pannexin was expressed at low levels by granule neurons in control mice, and appeared in puncta on their surface along the time course after KA was injected (7-point stars), and could occasionally be diffusely expressed in microglia (5-point star at 6 hpi). Pannexin expression was largely absent in apoptotic cells, either phagocytosed (arrows) or nonphagocytosed (arrowheads) in control and KA mice. We found some cases of nonphagocytosed apoptotic cells labeled with puncta of pannexin at 7 dpi (7-point star at 7 dpi). Scale bars = 20 μm. z = 14.7 μm.
(TIF)

S13 Fig Survival of newborn cells 1 dpi after KA. (**A**) Experimental design to test the effect of KA on 3 do (upper panel) and 8 do (lower panel). (**B**) Number of BrdU^+^ cells per septal hippocampus in saline or KA injected mice (*n* = 3–5 per group). 8 do cells were naturally less abundant than 3 do cells, reflecting the decreased survival of newborn cells. Nonetheless, KA did not significantly alter the number of BrdU^+^ cells born 3 or 8 d before. (**C**) Representative projections of confocal z-stacks of the DG of the hippocampus showing the colocalization between Ki67 (green) and BrdU (red), which had been injected 3 d before. The colocalization was a measure of the reentry of 3 do cells in the cell cycle and was identical in saline- and KA-injected mice. (**C**) Representative epifluorescent tiled image of the hippocampus and surrounding cortex of 2 and 6 mo mice injected with KA at 1 dpi stained with the neuronal activation marker c-fos. The same pattern of expression was found in young and mature mice throughout the DG, CA2, CA1, and the above cortex. (**D**) Ph index in the hippocampus (in % of apoptotic cells) in control and KA-injected mice at 2 and 6 mo (*n* = 4–5 per group). Scale bars = 50 μm (C). z = 28.7 μm (C, saline), 25.2 μm (C, KA). Underlying data is shown in **S1 Data**.
(TIF)

S1 Movie Basal motility of microglial processes by two-photon microscopy in the living cortex of CX3CR1^GFP/+^ mice after intrahippocampal injection of saline (1 dpi). Scale bar = 20 μm. Time is indicated as hr:min.
(AVI)

S2 Movie Basal motility of microglial processes by two-photon microscopy in the living cortex of CX3CR1^GFP/+^ mice after intrahippocampal injection of KA (1 dpi). Scale bar = 20 μm. Time is indicated as hr:min.
(AVI)

S1 Table Apoptosis and microglial phagocytosis in the human hippocampus. List of biopsy (Cruces University Hospital, Bilbao, Spain) and autopsy (Netherlands Brain Bank, NBB) hippocampal samples analyzed, including the diagnostic (ND, nondemented controls), the number of sections analyzed, the total number of apoptotic cells, the number of apoptotic cells phagocytosed by microglia, the Ph index, as well as age, sex, PM (hr:min) delay, and cause of death. Representative images can be found in **Fig 6**and **S8 Fig**.
(DOCX)

S2 Table Fatty acid composition of the dietary lipids. Percentage (in weight) in saturated, monounsaturated, omega 6 (Ω6) polyunsaturated, and omega 3 (Ω3) polyunsaturated fatty acids, as determined by gas chromatography. AA, arachidonic acid; ALA, α-linolenic acid; FAs, fatty acids; LA, linolenic acid; ND, not detected (under the limit for the detection by gas chromatography, <0.05%); PUFAs, polyunsaturated fatty acids.
(DOCX)

S3 Table qPCR primer sequences. List of primers used to amplify reference genes, cytokines, and glutamate receptor subunits. The gene name, Gene Bank accession number, amplicon size, sequence, and software used for their design are listed.
(DOCX)

## Abbreviations

ACSF: artificial cerebrospinal fluid
AP: anteroposterior
ATP: adenosine triphosphate
BBG: Brilliant Blue G
BrdU: bromo-deoxyuridine
CR3: complement receptor 3
CSF: colony stimulating factor
DG: dentate gyrus
do: days old
dpi: days postinjection
DV: dorsoventral
FACS: fluorescent-activated cell sorting
FBS: fetal bovine serum
HBSS: Hanks' balanced salt solution
hGFAP: human glial fibrillary acidic protein
hpi: hours postinjection
IL-1β: interleukin 1 beta
IL-6: interleukin 6
i.p.: intra-peritoneal
KA: kainic acid
LL: laterolateral
LPS: lipopolysaccharides
MerTK: Mer Tyrosine Kinase
MIC-1: macrophage inhibitory cytokine 1
mo: months old
mpi: months postinjection
MTLE: mesial temporal lobe epilepsy
NGS: normal goat serum
NMDA: N-methyl-D-aspartate
PBS: phosphate buffered saline
PFA: paraformaldehyde
Ph/A coupling: phagocytosis/apoptosis coupling ratio
PH3: phosphohistone 3
PI: propidium iodide
PM: postmortem
PND: postnatal day
POMC: proopiomelanocortin
RTqPCR: retrotranscription and quantitative polymerase chain reaction
SGZ: subgranular zone
ppu: parts per unit
TGFβ: transforming growth factor beta
Trem2: triggering receptor expressed in myeloid cells 2
TNFα: tumor necrosis factor alpha
UTP: uridine-5'-triphosphate
vEEG: video-electroencephalogram
wk: week
4-AP: 4-aminopyridine
