# Correction: Neuronal Hyperactivity Disturbs ATP Microgradients, Impairs Microglial Motility, and Reduces Phagocytic Receptor Expression Triggering Apoptosis/Microglial Phagocytosis Uncoupling

**DOI:** 10.1371/journal.pbio.1002508

**Published:** 2016-06-30

**Authors:** Oihane Abiega, Sol Beccari, Irune Diaz-Aparicio, Agnes Nadjar, Sophie Layé, Quentin Leyrolle, Diego Gómez-Nicola, María Domercq, Alberto Pérez-Samartín, Víctor Sánchez-Zafra, Iñaki Paris, Jorge Valero, Julie C. Savage, Chin-Wai Hui, Marie-Ève Tremblay, Juan J. P. Deudero, Amy L. Brewster, Anne E. Anderson, Laura Zaldumbide, Lara Galbarriatu, Ainhoa Marinas, Maria dM. Vivanco, Carlos Matute, Mirjana Maletic-Savatic, Juan M. Encinas, Amanda Sierra

The affiliations for three of the authors, Mirjana Maletic-Savatic, Juan M. Encinas, and Amanda Sierra, are listed incorrectly in the original article. Dr. Maletic-Savatic is not affiliated with Centre de recherche du CHU de Québec, but rather Baylor College of Medicine. Dr. Encinas is not affiliated with Baylor College of Medicine, but rather Ikerbasque Foundation. Dr. Sierra is not affiliated with Baylor College of Medicine, but rather Ikerbasque Foundation. The complete list of authors with the correct affiliations is listed below:

Oihane Abiega^1,2☯^, Sol Beccari^1,2☯^, Irune Diaz-Aparicio^1,2☯^, Agnes Nadjar^3^, Sophie Layé^3^, Quentin Leyrolle^3^, Diego Gómez-Nicola^4^, María Domercq^1,2^, Alberto Pérez-Samartín^1,2^, Víctor Sánchez-Zafra^1,2^, Iñaki Paris^1,2^, Jorge Valero^1,2,5^, Julie C. Savage^6,7^, Chin-Wai Hui^6,7^, Marie-Ève Tremblay^6,7^, Juan J. P. Deudero^8^, Amy L. Brewster^8^, Anne E. Anderson^8^, Laura Zaldumbide^9^, Lara Galbarriatu^9^, Ainhoa Marinas^9^, Maria dM. Vivanco^10^, Carlos Matute^1,2^, Mirjana Maletic-Savatic^8^, Juan M. Encinas^1,2,5^, Amanda Sierra^1,2,5^*

1 Achucarro Basque Center for Neuroscience, Bizkaia Science and Technology Park, Zamudio, Spain,

2 University of the Basque Country, Leioa, Spain,

3 Université Bordeaux Segalen, Bordeaux, France,

4 Centre for Biological Sciences, University of Southampton, Southampton, United Kingdom,

5 Ikerbasque Foundation, Bilbao, Spain,

6 Centre de recherche du CHU de Québec, Axe Neurosciences, Québec, Canada,

7 Université Laval, Département de médecine moléculaire, Québec, Canada,

8 Baylor College of Medicine, The Jan and Dan Duncan Neurological Research Institute at Texas Children’s Hospital, Houston, Texas, United States of America,

9 University Hospital of Cruces, Bilbao, Spain,

10 CIC BioGUNE, Derio, Spain

☯ OA, SB, and IDA contributed equally to this work.

* a.sierra@ikerbasque.org
