# Correction: Neuronal Hyperactivity Disturbs ATP Microgradients, Impairs Microglial Motility, and Reduces Phagocytic Receptor Expression Triggering Apoptosis/Microglial Phagocytosis Uncoupling

**DOI:** 10.1371/journal.pbio.1002554

**Published:** 2016-09-20

**Authors:** Oihane Abiega, Sol Beccari, Irune Diaz-Aparicio, Agnes Nadjar, Sophie Layé, Quentin Leyrolle, Diego Gómez-Nicola, María Domercq, Alberto Pérez-Samartín, Víctor Sánchez-Zafra, Iñaki Paris, Jorge Valero, Julie C. Savage, Chin-Wai Hui, Marie-Ève Tremblay, Juan J. P. Deudero, Amy L. Brewster, Anne E. Anderson, Laura Zaldumbide, Lara Galbarriatu, Ainhoa Marinas, Maria dM. Vivanco, Carlos Matute, Mirjana Maletic-Savatic, Juan M. Encinas, Amanda Sierra

The authors would like to report a typographical error that states the concentration of staurosporine used for the induction of apoptosis is 10 μM. The actual concentration used is 1 μM. The authors confirm that the changes do not affect the results of the experiments.

The typographical error can be found in the following two instances and are corrected here:

In the Figure 8 legend, Panel F (page 17 in the PDF):

“Primary cultures were pre-treated with KA (1 mM) for 2 h prior to adding apoptotic NE-4C cells (treated with 5 μM CM-DiI for 25 min and 1 μM staurosporine for 4 h).”

Under Materials and Methods, in the second paragraph under the ‘Primary Microglia Cultures’ heading (Page 32 in the PDF):

“NE-4C were previously labeled with the membrane marker CM-DiI (5 μM; 10 min at 37°C, 15 min at 4°C; Invitrogen) and treated with staurosporine (1 μM, 4h; Sigma) to induce apoptosis.”

## References

[1] Abiega O, Beccari S, Diaz-Aparicio I, Nadjar A, Layé S, Leyrolle Q, et al (2016) Neuronal Hyperactivity Disturbs ATP Microgradients, Impairs Microglial Motility, and Reduces Phagocytic Receptor Expression Triggering Apoptosis/Microglial Phagocytosis Uncoupling. PLoS Biol 14(5): e1002466 doi: 10.1371/journal.pbio.1002466 27228556 10.1371/journal.pbio.1002466 PMC4881984

