# Supplementary figures and images for: Neuronal Hyperactivity Disturbs ATP Microgradients, Impairs Microglial Motility, and Reduces Phagocytic Receptor Expression Triggering Apoptosis/Microglial Phagocytosis Uncoupling
Source: PLoS Biol. 2016 May 26;14(5):e1002466. doi: 10.1371/journal.pbio.1002466 (PMC4881984; doi:10.1371/journal.pbio.1002466)

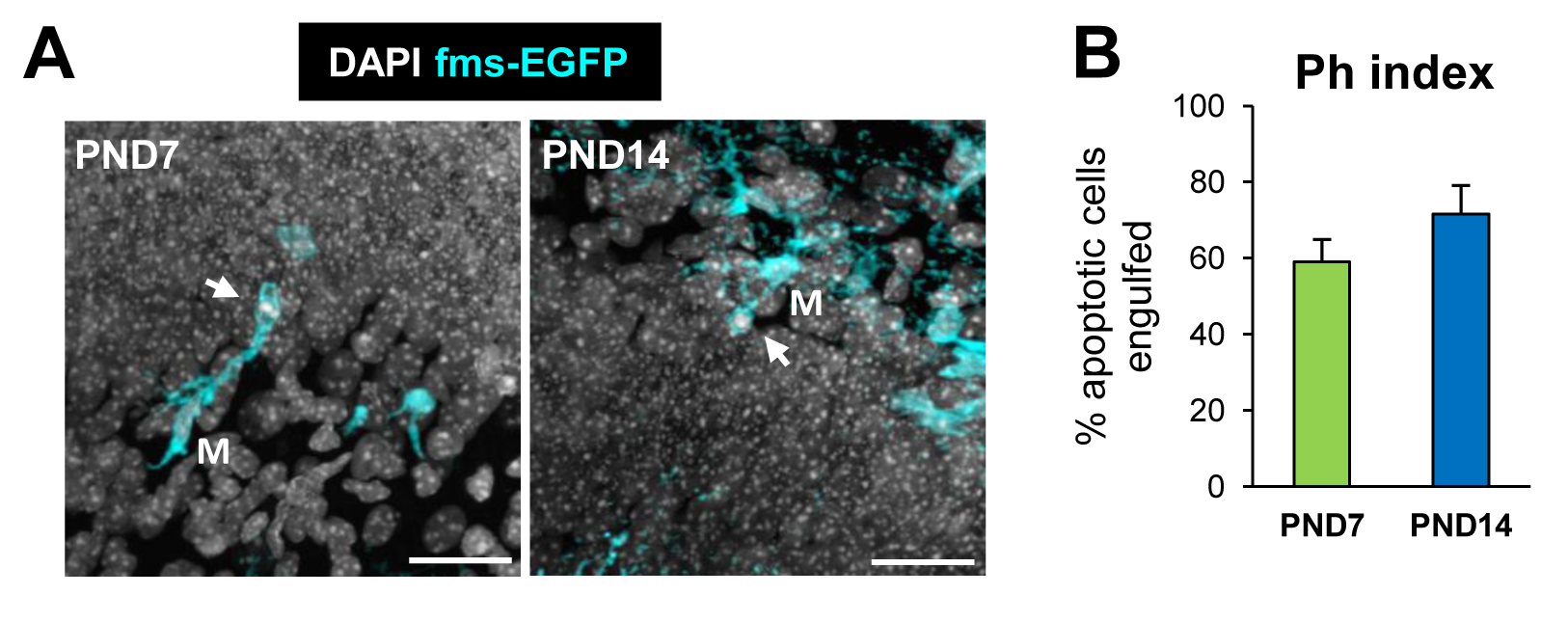

Supplement: S1 Fig — (A) Representative confocal z-stack projections of the DG of the hippocampus at PND d 7 (PND7) and PND14 of fms-EGFP mice, the ages when organotypic cultures were cultured (PND7) and were used for experiments (PND7+7DIV). Apoptotic (pyknotic, white, DAPI; arrows) cells were phagocytosed by terminal or en passant branches of microglia (fms-EGFP+, cyan; M). Scale bars = 50 μm. z = 16.8 μm. (B) Ph index in the DG at PND7 and 14 (in % of apoptotic cells; n = 3–4 per group). Bars represent mean ± SEM. * indicates p < 0.05 and ** indicates p < 0.01 by one-tail Student´s t test. Underlying data is shown in S1 Data. (TIF) [file pbio.1002466.s014.tif]

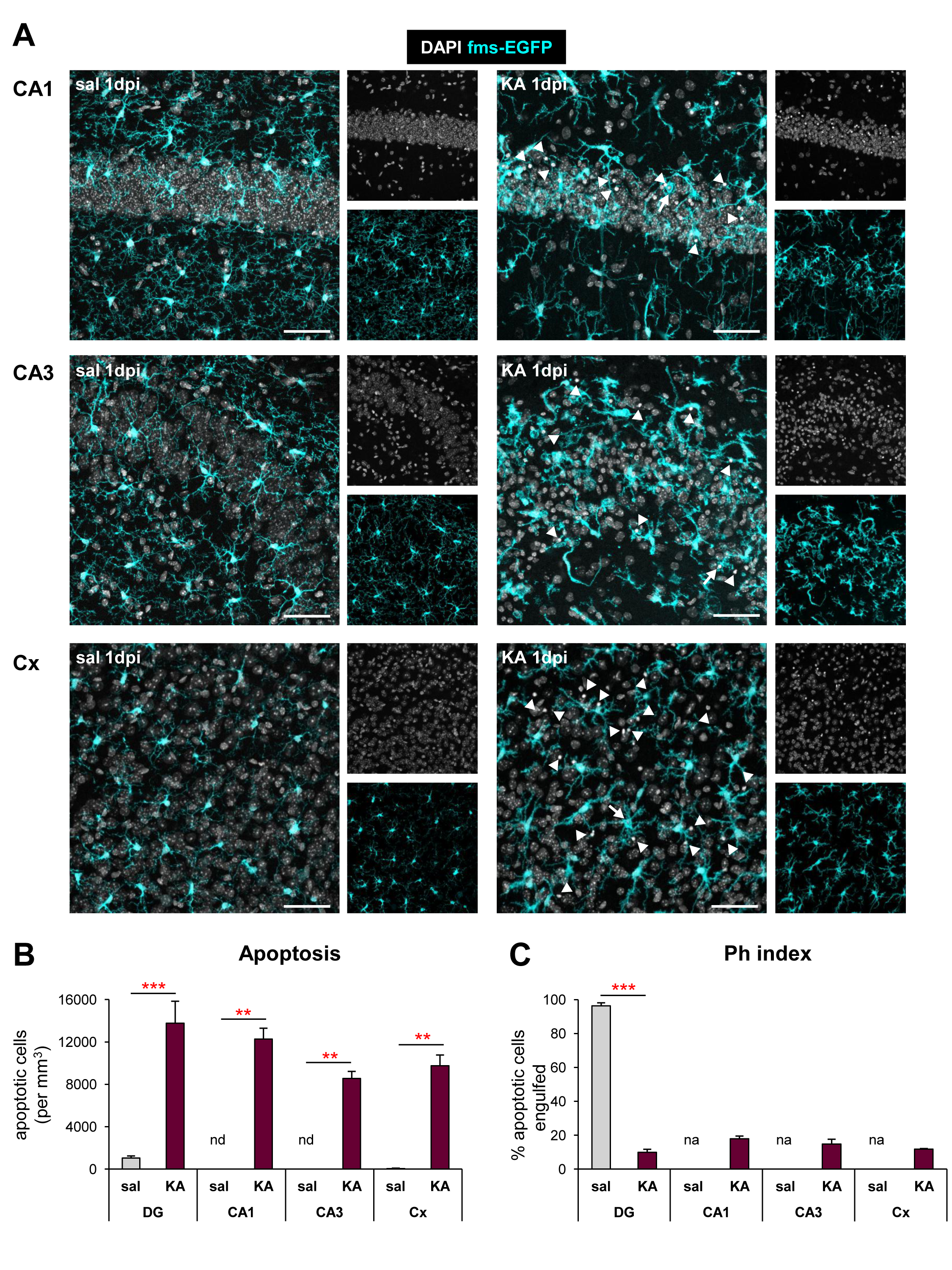

Supplement: S2 Fig — (A) Representative confocal z-stack projections of the CA1, CA3 regions of the hippocampus, and cortex (Cx) of 2 mo fms-EGFP mice injected with saline (left panels) or KA (right panels) at 1 dpi. Apoptotic cells (pyknotic, white, DAPI; arrowheads) are largely absent in control conditions but present in KA-treated mice in the three regions. Some apoptotic cells were phagocytosed (arrows) by microglia (fms-EGFP+, cyan) but most were not (arrowheads). Similar images of the DG are shown in Fig 3B. Scale bars = 50 μm. z = 18.2 μm (except in CA1 KA1 dpi = 20.3 μm, CA3 sal 1 dpi = 17.5 μm and Cx KA 1 dpi = 19.6 μm). (B) Density of apoptotic (pyknotic/karyorrhectic and act-casp3+) per mm3 (n = 3 per region and treatment). (C) Ph index (in % of apoptotic cells) in the different brain regions after KA. nd, not detected; na, not applicable. Bars represent mean ± SEM. ** indicates p < 0.01, and *** indicates p < 0.001 by Student’s t test. Underlying data is shown in S1 Data. (TIF) [file pbio.1002466.s015.tif]

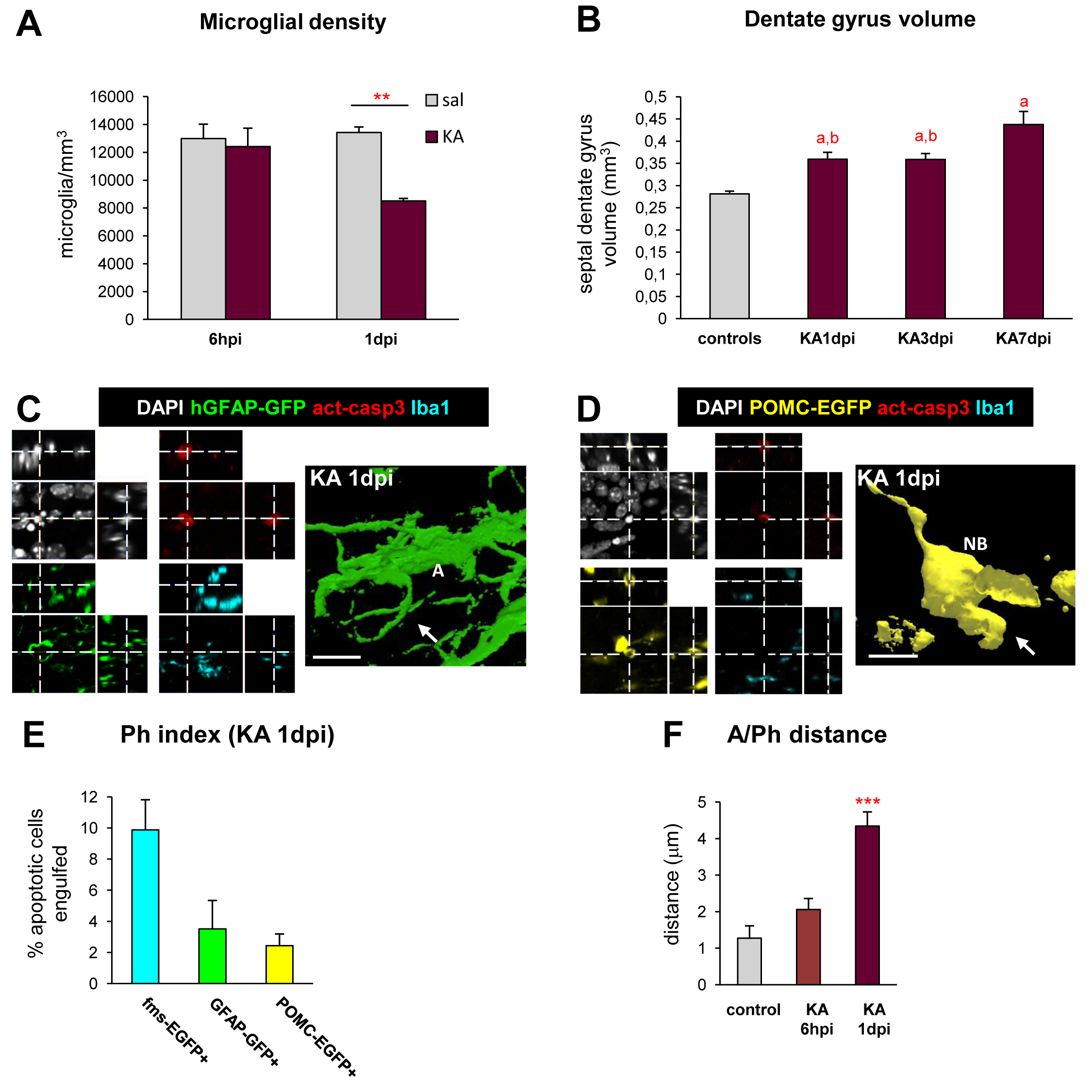

Supplement: S3 Fig — (A) Density of microglia (cells/mm3) in saline and KA-injected mice (n = 3–5 per group). At 1 dpi, KA induced a significant decrease in the density of microglia. (B) Volume of the septal DG (mm3) in saline and KA-injected mice (n = 3–5 per group). The volume occupied by the DG was assessed in the septal hippocampus (spanning from −1 mm to −2.5 mm in the AP axes, from Bregma) in control animals (injected with saline, pooled from different time points for robustness) or after injection of KA at 1, 3, and 7 dpi (no changes after 6 hpi were found). (C) Representative orthogonal projection (upper panel) and 3-D-rendered image (lower panel) of a confocal z-stack showing an apoptotic cell (pyknotic, white, DAPI) expressing activated-caspase 3 (act-casp3+, red) phagocytosed by a hGFAP+ astrocyte (green), nearby a microglial cell (Iba1+, cyan). (D) Representative orthogonal projection (upper panel) and 3-D-rendered image (lower panel) of a confocal z-stack showing an apoptotic cell (pyknotic, white, DAPI) expressing activated-caspase 3 (act-casp3+, red) phagocytosed by a POMC+ neuroblast (yellow), nearby a microglial cell (Iba1+, cyan). (E) Ph index of microglia, astrocytes and neuroblasts (in %) at 1 dpi after the injection of KA (n = 3–4 per group). At 1 dpi, the impaired microglia remained the major phagocytic cell in the hippocampus, as it engulfed a higher percentage of apoptotic cells. (F) Average distance (in μm) between apoptotic nuclei and the closest (perpendicular) microglial process. Apoptotic cells were analyzed from 3 animals per group (n = 6, 73, and 189 cells for control, KA 6 hpi and KA 1 dpi, respectively). The distance increased significantly by 1 dpi after KA. Bars represent mean ± SEM. In A, ** indicates p < 0.01 by Holm-Sidak posthoc test after two-way ANOVA was significant at p < 0.05. In B, a indicates p < 0.05 versus control, b indicates p < 0.05 versus KA 7 dpi by Holm-Sidak posthoc test after one-way ANOVA was significant at p < 0.05. In F, *** ind [file pbio.1002466.s016.tif]

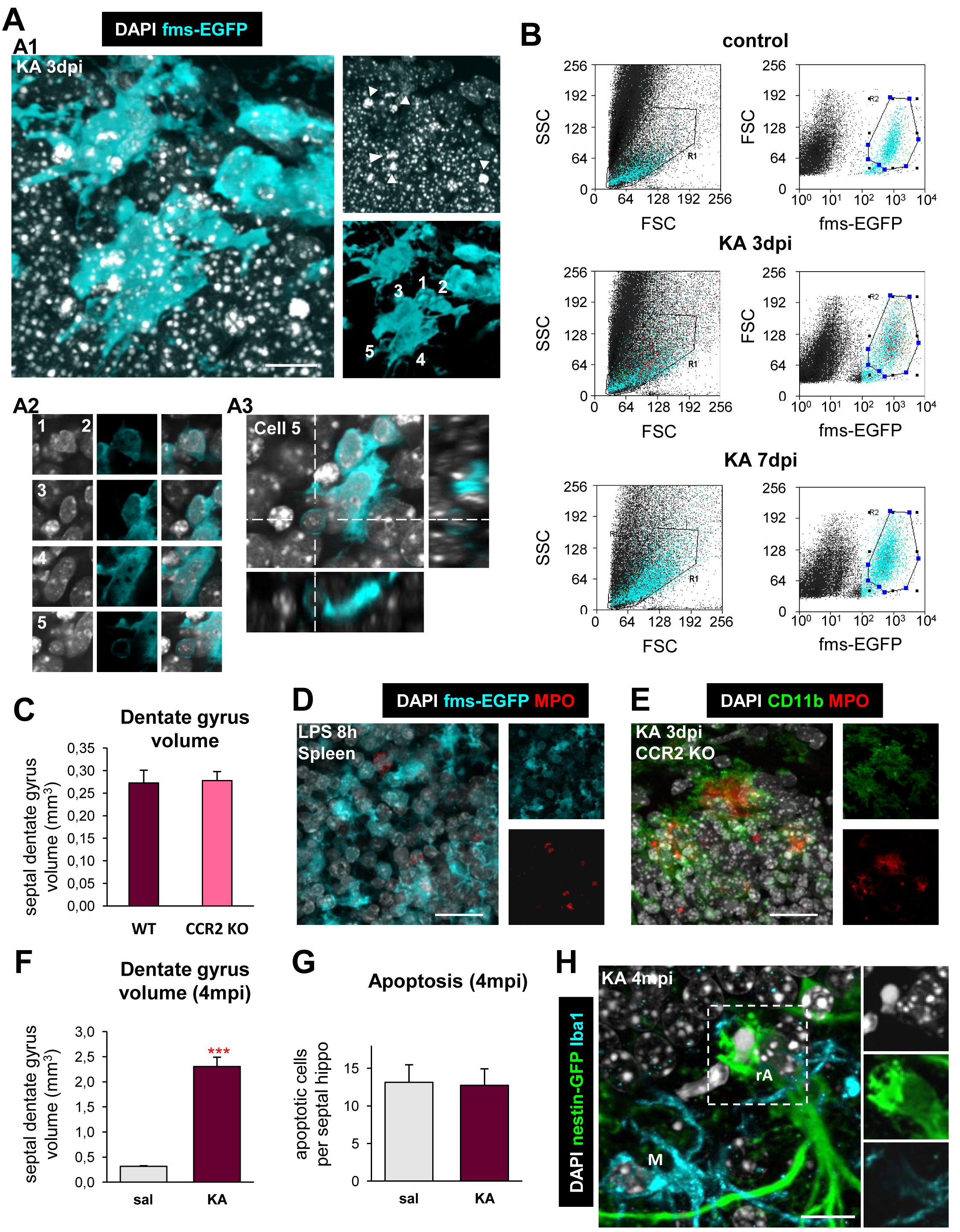

Supplement: S4 Fig — (A) Representative projection of a confocal z-stack showing several large multinucleated phagoptotic microglia (fms-EGFP+, cyan) from the hippocampus of a KA mouse (3 dpi). (A1) 3-D-rendered image showing the continuum of EGFP through the microglial cytoplasm and within their nuclei. Up to five nuclei were contained. (A2) Panel showing each nucleus individually. Nucleus 5 was small but not pyknotic and was surrounded by a pouch of microglial cytoplasm. (A3) Orthogonal projections of nucleus 5 showing its complete engulfment by microglial processes (phagoptosis). Arrowhead point towards a nonengulfed apoptotic cell. (B) Gating strategy used for the analysis of CD45 expression in microglia from control and KA-injected mice (3 and 7 dpi). First, debris was excluded using the R1 gate in FSC versus SSC. Next, fms-EGFP+ cells were gated in R2 in EGFP vs FSC. A small population of microglial processes was excluded from R2 [10]. CD45low cells are shown in cyan and CD45high in red (as in Fig 5). Original FSC files can be found in S2–S5 Data (control), S6–S9 Data (KA 3 dpi), and S10–S13 Data (KA 7 dpi). (C) Septal DG volume (in mm3) in WT and CCR2 KO mice at 3 dpi. (D) Representative projection of a confocal z-stack showing neutrophils in the spleen challenged with LPS (8 h), stained with myeloperoxidase (MPO, red) in fms-EGFP+ cells. (E) Representative projection of a confocal z-stack showing neutrophils in the DG of a CCR2 KO mice injected with KA at 3 dpi. Neutrophils expressed CD11b (green) and MPO (red). (F) Septal DG volume (in mm3) in saline and KA-injected mice at 4 mpi. (G) Number of apoptotic cells per septal hippocampus in saline (n = 7) and KA (n = 8) mice at 4 mpi after the injection of KA. (H) Representative projection of a confocal z-stack showing phagocytosis by a reactive astrocyte in the hippocampus of a KA mice (4 mpi). Arrows point towards the phagocytosed apoptotic cell. rA, reactive astrocyte; M, microglia. Scale bars = 10 μm (A, G), 20 μm (C, D). z = 17 [file pbio.1002466.s017.tif]

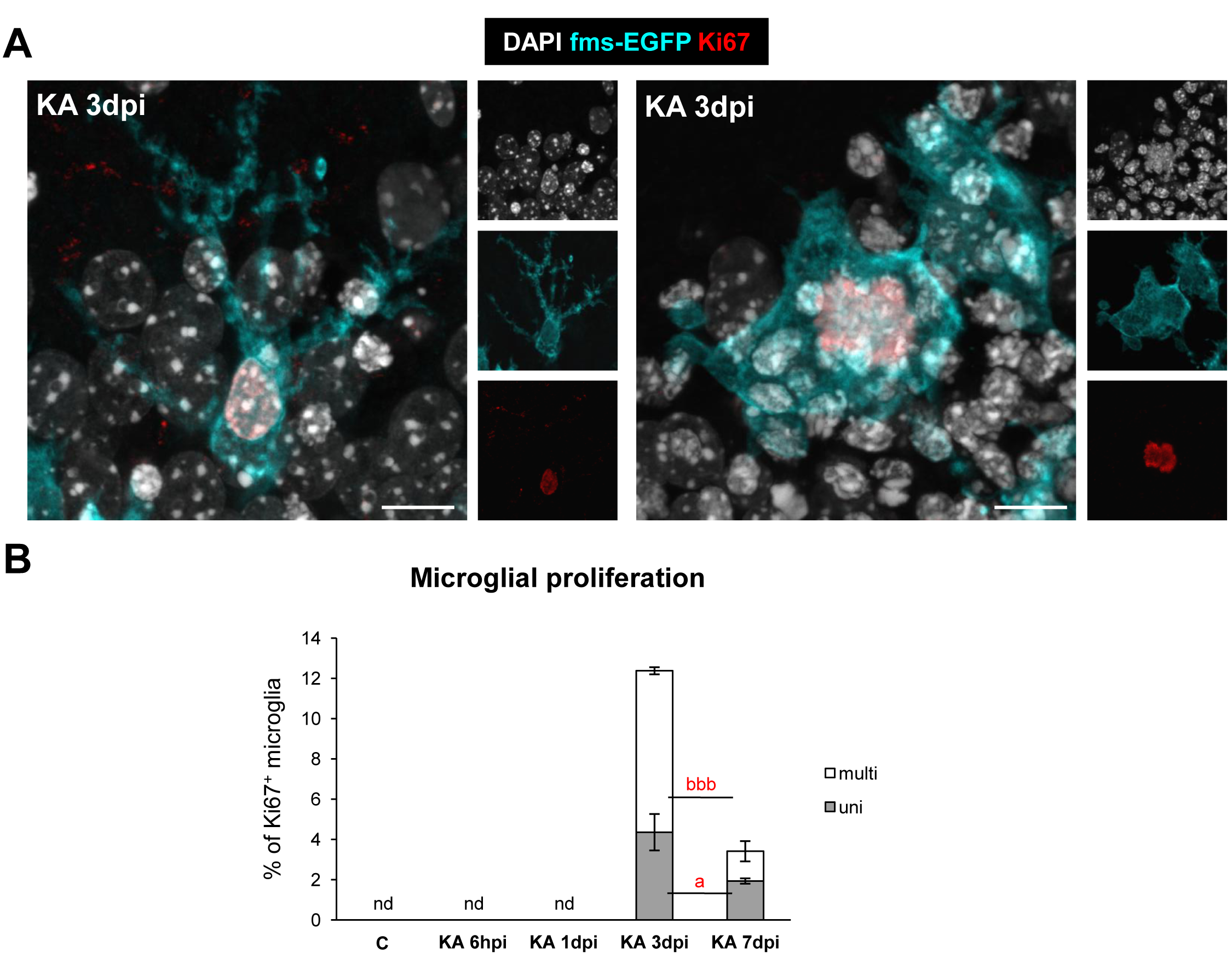

Supplement: S5 Fig — (A) Representative confocal z-stack projection of uni- (left) and multinucleated (right) microglia (fms-EGFP+, cyan) in the DG 3 dpi after KA. Proliferating cells were labeled with Ki67 (red) and nuclei with DAPI (white). Scale bars = 10μm. z = 9.8 μm (B) Percentage of uni- (grey) and multinucleated (white) microglia labeled with Ki67 from 6hpi to 7 dpi. Bars represent mean ± SEM. nd, not detectable. a indicates p < 0.05 between uninucleated microglia at 3 and 7 dpi, bbb indicates p < 0.001 between multinucleated microglia at 3 and 7 dpi by Student´s t test. Underlying data is shown in S1 Data. (TIF) [file pbio.1002466.s018.tif]

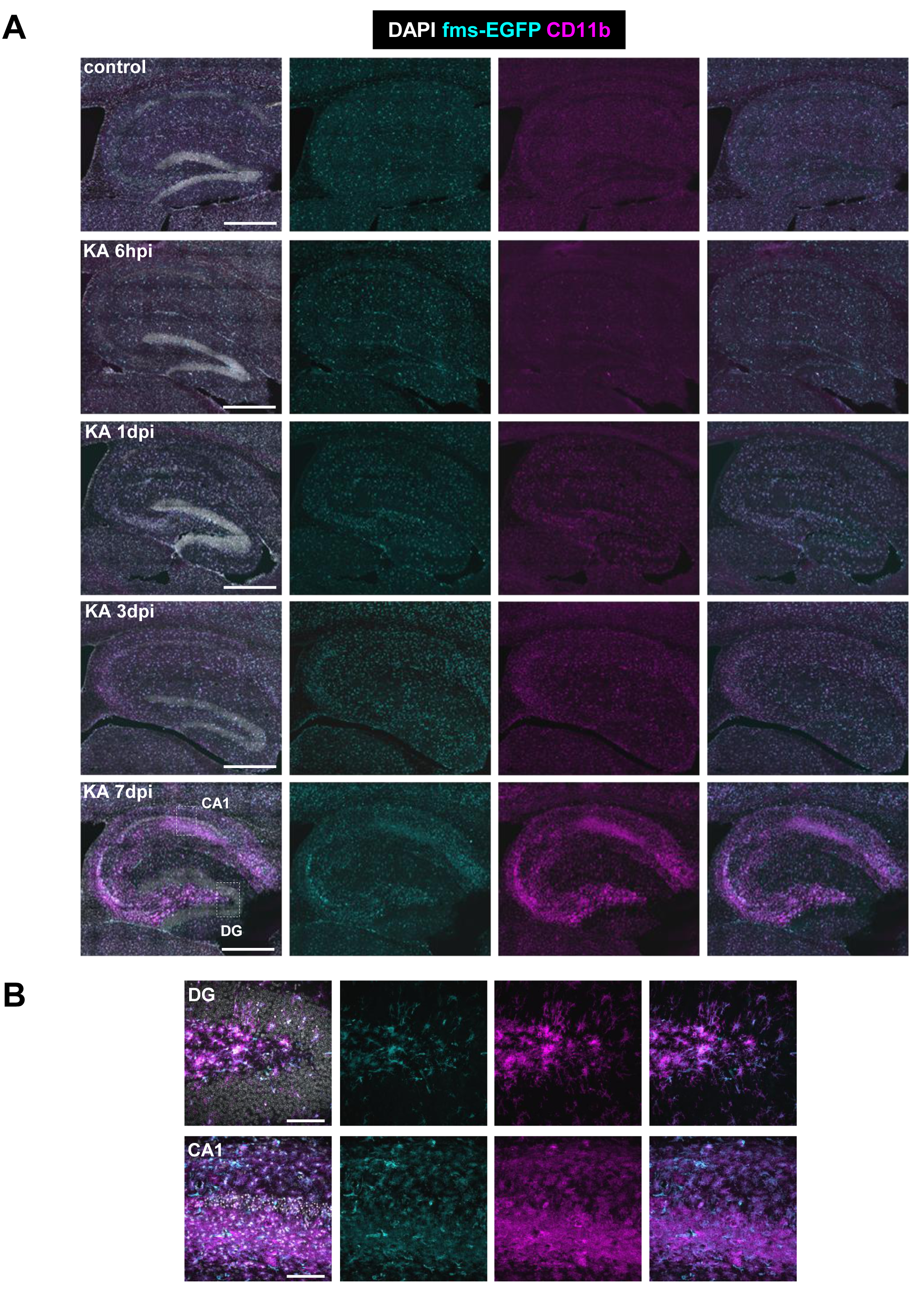

Supplement: S6 Fig — (A) Representative confocal z-stack projections of the hippocampus in control (not injected) and KA-injected mice from 6 hpi to 7 dpi. Nuclei are labeled with DAPI (white), and microglia with fms-EGFP (cyan) and CD11b (magenta). The expression of CD11b increased over the time course. Scale bars = 500 μm. z = 7.7 μm (control, KA 6 hpi), 9.1 μm (KA 1 dpi), 9.8 μm (KA 3 dpi, 7 dpi). (B) High magnification inserts of the DG and CA1 region in KA mice at 7 dpi. Scale bars = 100 μm. z = 14.7 μm (DG), 11.9 μm (CA1). (TIF) [file pbio.1002466.s019.tif]

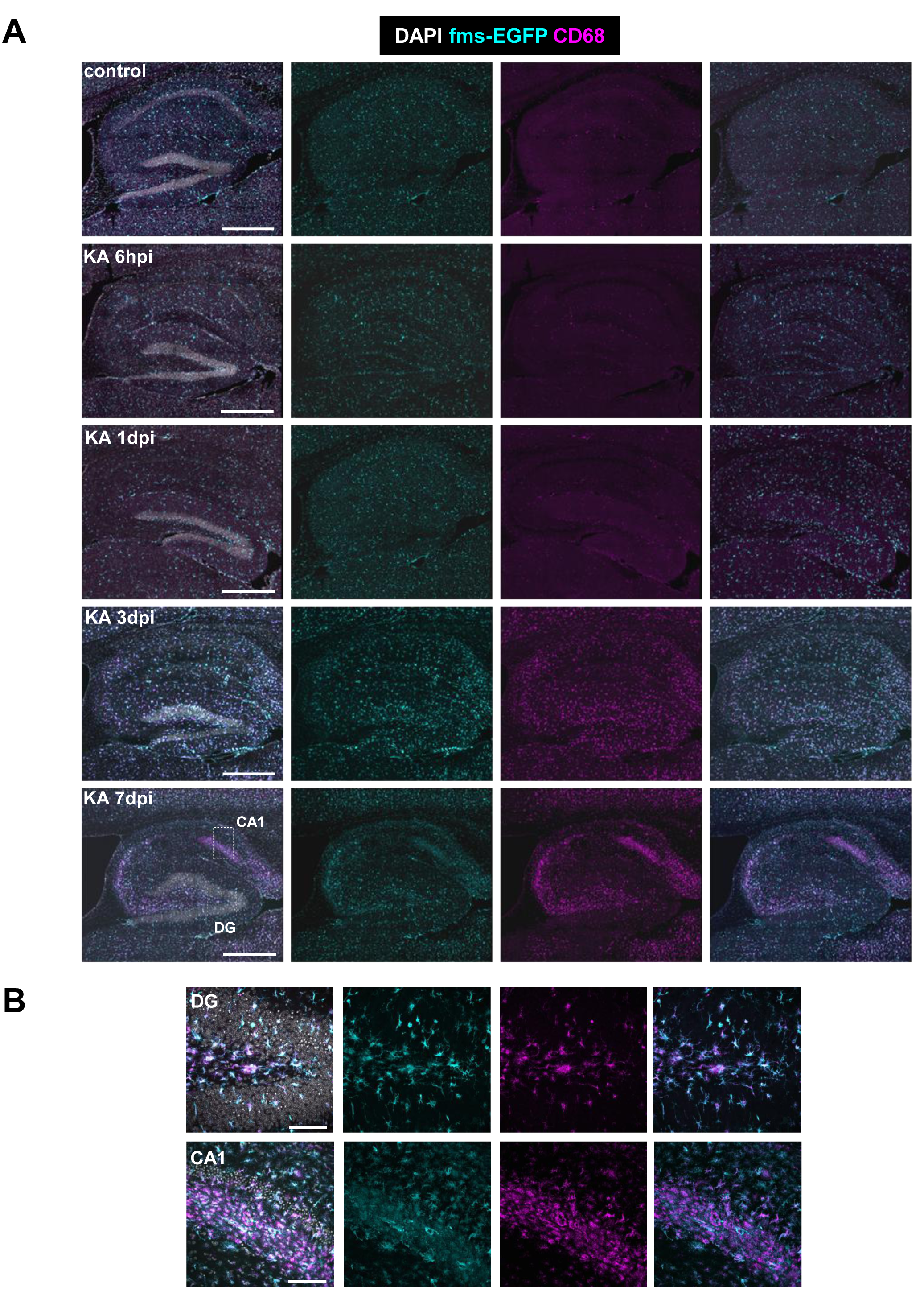

Supplement: S7 Fig — (A) Representative confocal z-stack projections of the hippocampus in control (not injected) and KA-injected mice from 6 hpi to 7 dpi. Nuclei are labeled with DAPI (white), and microglia with fms-EGFP (cyan) and CD68 (magenta). The expression of CD68 increased over the time course. Scale bars = 500 μm. z = 9.8 μm (control, KA 7 dpi), 8.4 μm (KA 6 hpi), 10.5 μm (KA 1 dpi, 3 dpi). (B) High magnification inserts of the DG and CA1 region in KA mice at 7 dpi. Scale bars = 100 μm. z = 12.6 μm (DG), 11.9 μm (CA1). (TIF) [file pbio.1002466.s020.tif]

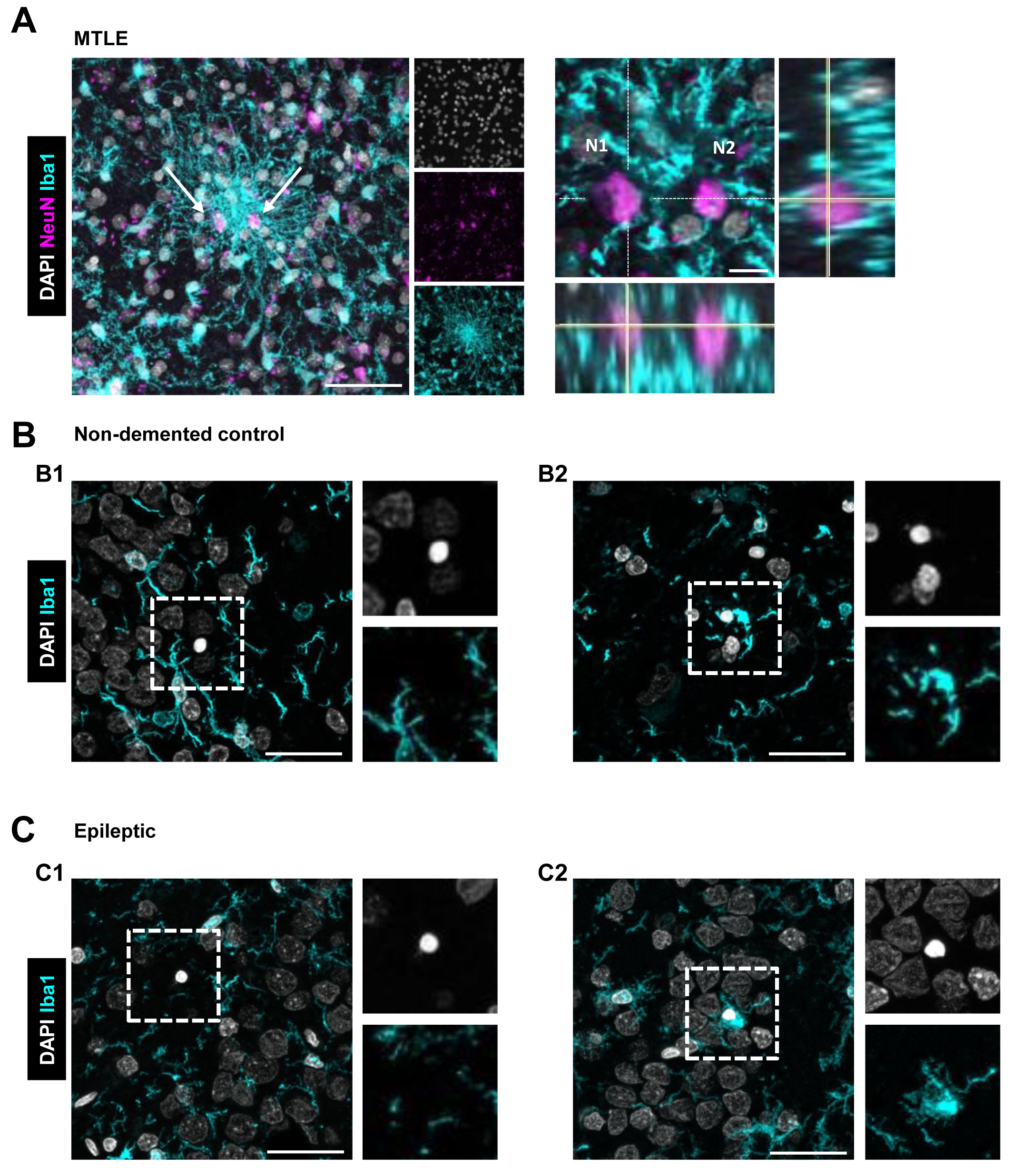

Supplement: S8 Fig — (A). Representative confocal z-stack projection of two neurons in the hilus (NeuN+, magenta; arrows) surrounded by a mesh of microglial processes (Iba1+) in the hippocampus of biopsy tissue obtained from an MTLE patient. Nuclei are shown in white (DAPI). The right panel shows an orthogonal projection of the same cells (N1 and N2). (B) Representative confocal z-stack projections of apoptotic cells (pyknotic, DAPI, white) not phagocytosed (B1, in the granular layer) and phagocytosed (B2, in the hilus) by microglia (Iba1+, cyan) in the hippocampus of autopsy tissue from a nondemented control. (C) Representative confocal z-stack projections of apoptotic cells (pyknotic, DAPI, white) not phagocytosed (C1, in the hilus) and phagocytosed (C2, in the granular layer) by microglia (Iba1+, cyan) in the hippocampus of autopsy tissue from an epileptic patient. The number of engulfed apoptotic cells evaluated is shown in S1 Table. Scale bars = 50 μm (A), 20 μm (B, C). z = 15.7 μm (A), 3.5 μm (B1), 8.05 μm (B2), 4.55 μm (C1), 9.1 μm (C2). (TIF) [file pbio.1002466.s021.tif]

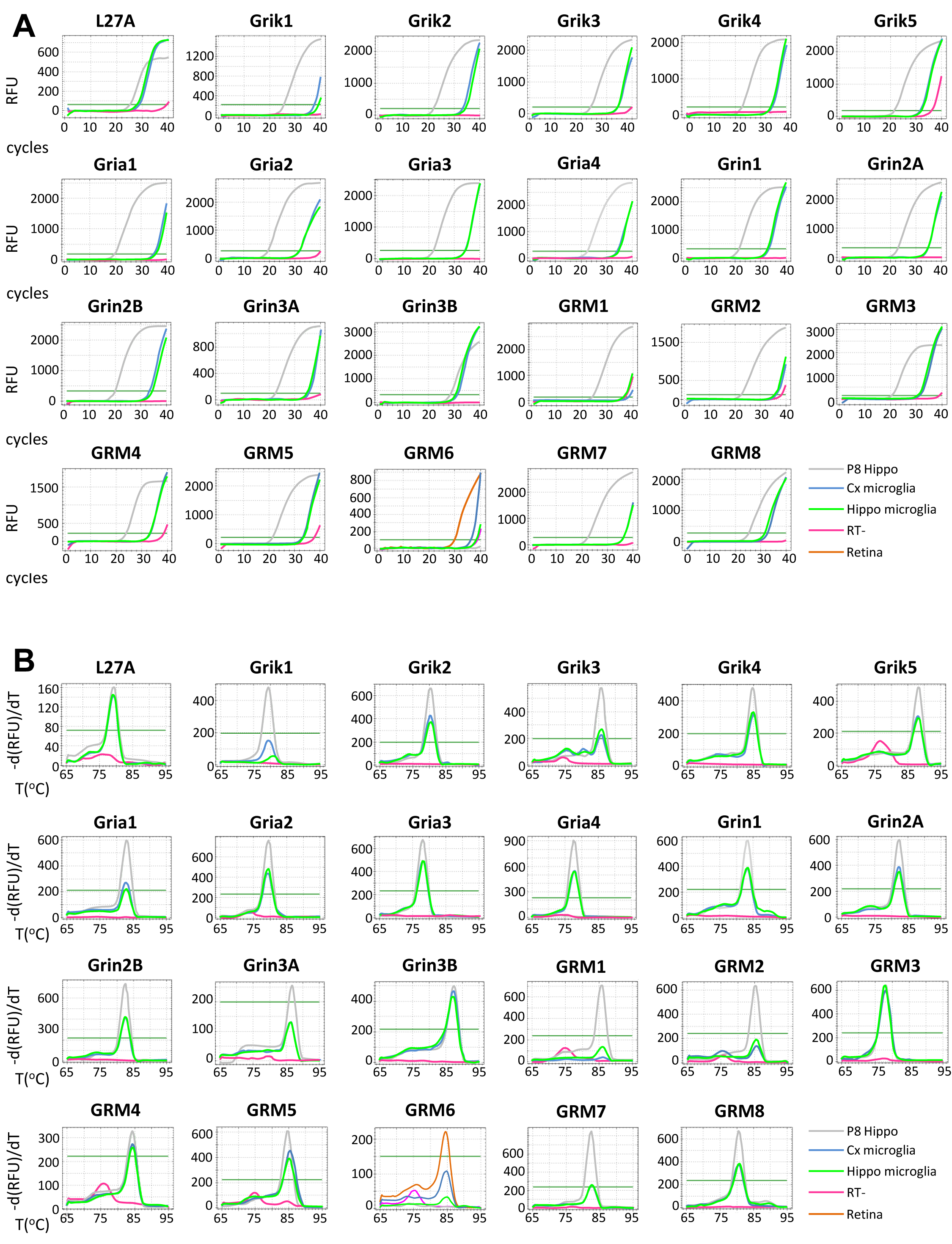

Supplement: S9 Fig — Microglia was FACS-sorted from the hippocampus and the cortex of 2 mo mice, and their expression of ionotropic and metabotropic glutamate receptor subunit assessed by RTqPCR. Two PND8 hippocampi were used as positive control (except for Grm6, where the retina of a 2 mo mouse was used), and the RT- as negative control. (A) Amplification plots for each subunit showing the cycle versus the RFU (relative fluorescent units). The threshold level of fluorescence used to determine the threshold cycle is shown as a straight dark green line. Microglia had a low, but consistent expression of all subunits above the threshold and clearly different from the RT-. (B) Denaturing curves for each subunit showing the increase in temperature versus the decrement in fluorescence. All primers used were checked against forming primer dimer or other nonspecific products. (TIF) [file pbio.1002466.s022.tif]

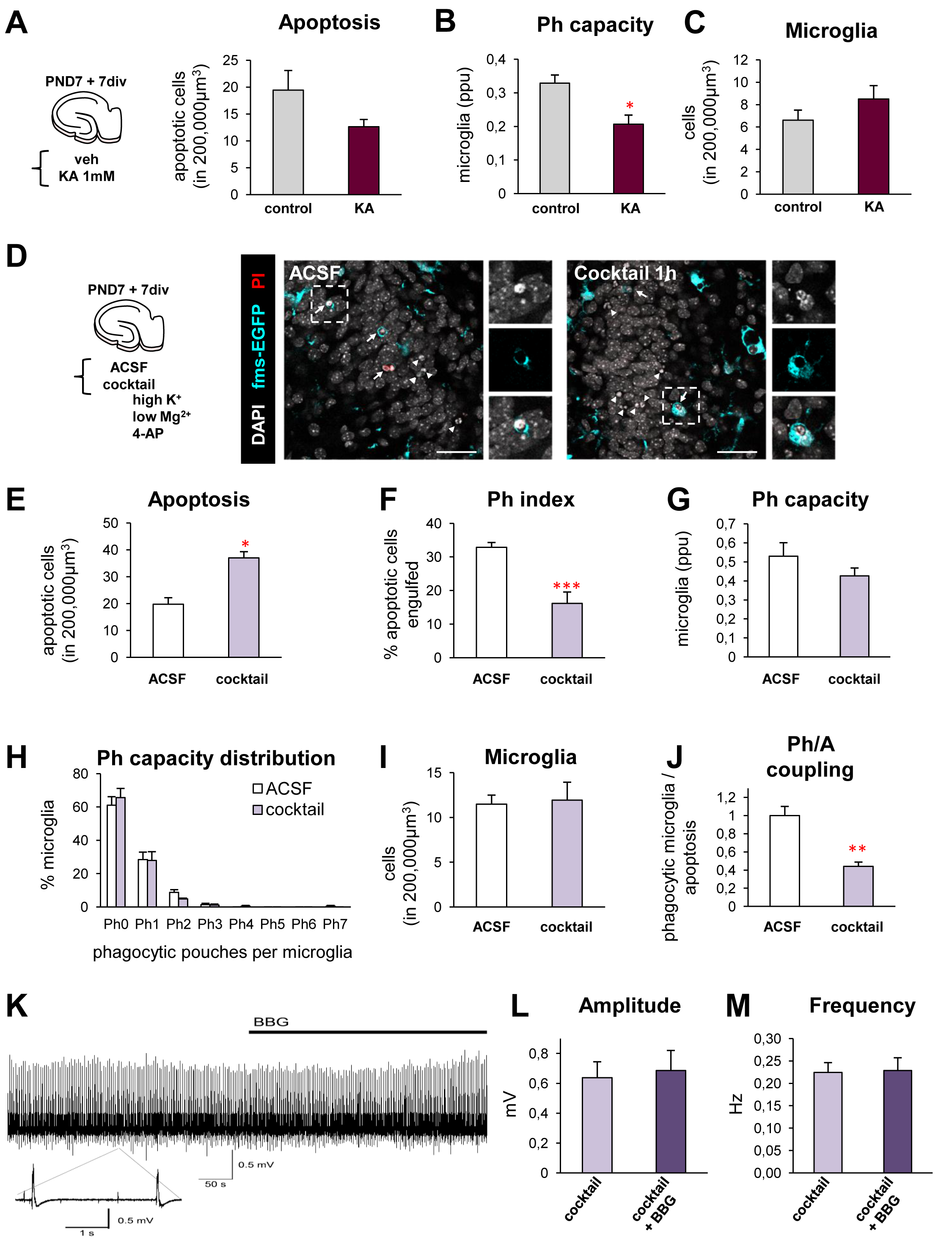

Supplement: S10 Fig — (A) Number of apoptotic cells in a 200.000 μm3 volume in organotypic slices treated with KA (1 mM, 6 h). No significant differences were found between KA (n = 5) and control (vehicle; n = 3) slices, although there was a tendency to found fewer apoptotic cells in KA-treated slices (p = 0.08). (B) Weighted Ph capacity (in ppu) in organotypic slices treated with KA. (C) Number of microglia within the slice in a 200.000 μm3 volume in organotypic slices treated with KA. (D) Experimental design and representative images of the DG of hippocampal organotypic slices treated with ACSF (control) or an epileptogenic cocktail (high K+, low Mg2+, 4-AP) for 1 h. Normal or apoptotic (pyknotic/karyorrhectic) nuclear morphology was visualized with DAPI (white), microglia by the transgenic expression of fms-EGFP (cyan), and membrane permeability (characteristic of necrotic cells) by PI (red). High magnification inserts show details of phagocytosed apoptotic cells in the two conditions. Arrows, phagocytosed cells; arrowheads, non-phagocytosed cell. Scale bars = 30 μm. (E) Number of dead apoptotic cells in 200.000 μm3 of the DG in organotypic slices treated with the epileptogenic cocktail. (F) Ph index in organotypic slices (% of apoptotic cells phagocytosed) treated with the epileptogenic cocktail. Note that the Ph index in ACSF-treated slices is higher than in organotypic culture media-treated slices (Fig 1). (G) Weighted Ph capacity of microglia (in parts per unit, ppu). (H) Histogram showing the Ph capacity of microglia (in % of cells). (I) Number of microglial cells. (J) Ph/A coupling (in fold-change) in organotypic slices treated with the epileptogenic cocktail. (K) Extracellular recording of the seizure activity induced by the epileptogenic cocktail before and after the purinergic antagonist BBG was added in acute hippocampal slices. The effect of this drug in microglial currents is shown in Fig 9A–9C. (L) Spike amplitude (in mV) induced by the epileptogenic cocktail. (M) Spike f [file pbio.1002466.s023.tif]

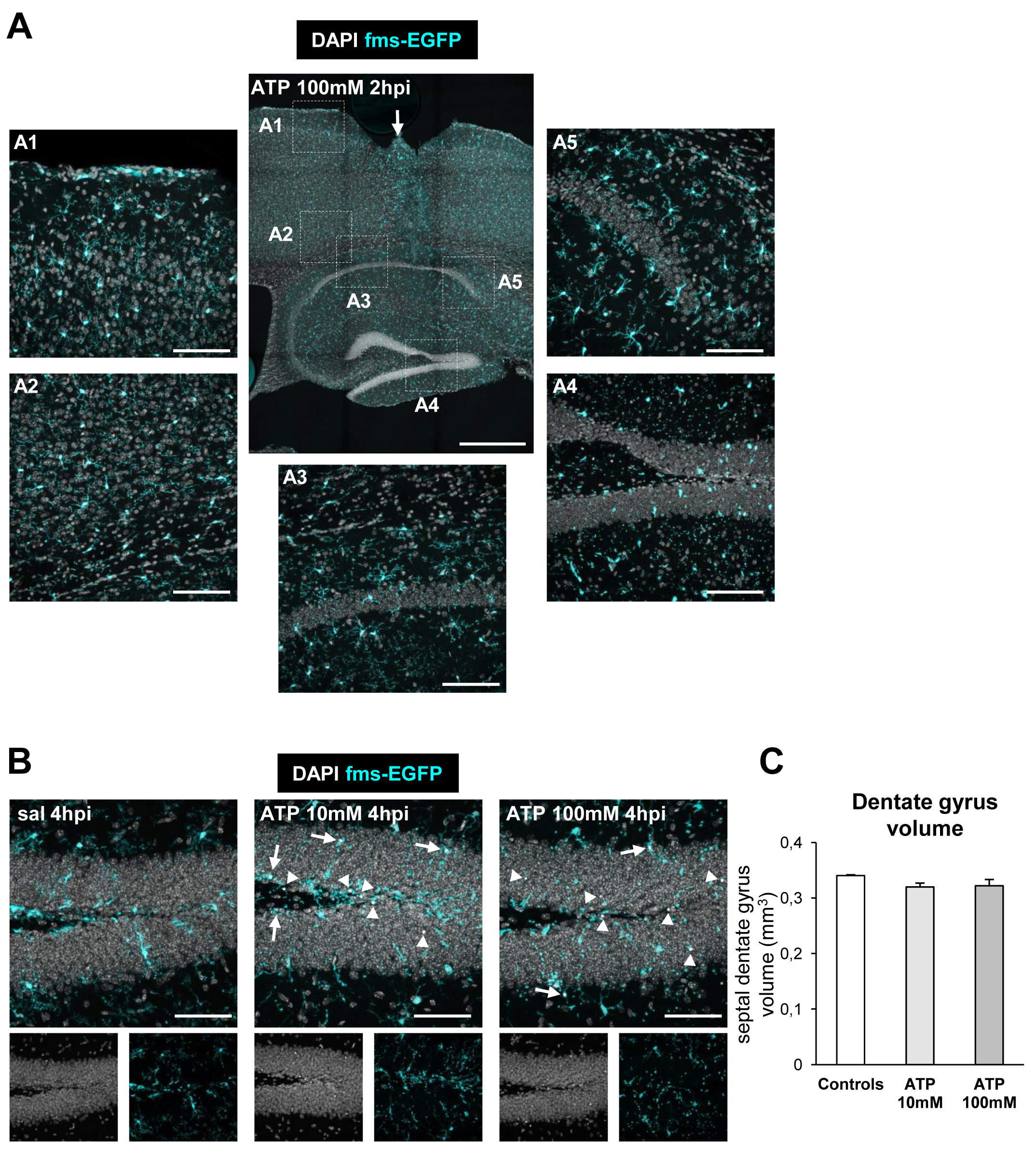

Supplement: S11 Fig — (A) Tiled confocal z-stack of the injection site in ATP-injected mice (100 mM, 2h). Note that the injected volume is larger than in the KA injections (1 μl versus 50 nL) and thus the tissue damage is more apparent. Nuclei are shown with DAPI in white and microglia is visualized with fms-EGFP in cyan. Inserts show details of the cortex (A1, A2), CA3 (A3), DG (A4), and CA1 (A5). The effect of injected ATP was restricted to the DG, as determined by the change in microglial morphology. (B) Representative confocal z-stack of the DG in mice injected with vehicle (control) or ATP (10 or 100 mM) at 4 hpi. (C) Septal DG volume (in mm3) in saline and ATP-injected mice at 4 hpi. Scale bars = 500 μm (A, tiled image), 100 μm (A, details), 50μm (B). z = 25.2 μm (A, tiled image), 16.8 μm (A, details), 14 μm (B). Underlying data is shown in S1 Data. (TIF) [file pbio.1002466.s024.tif]

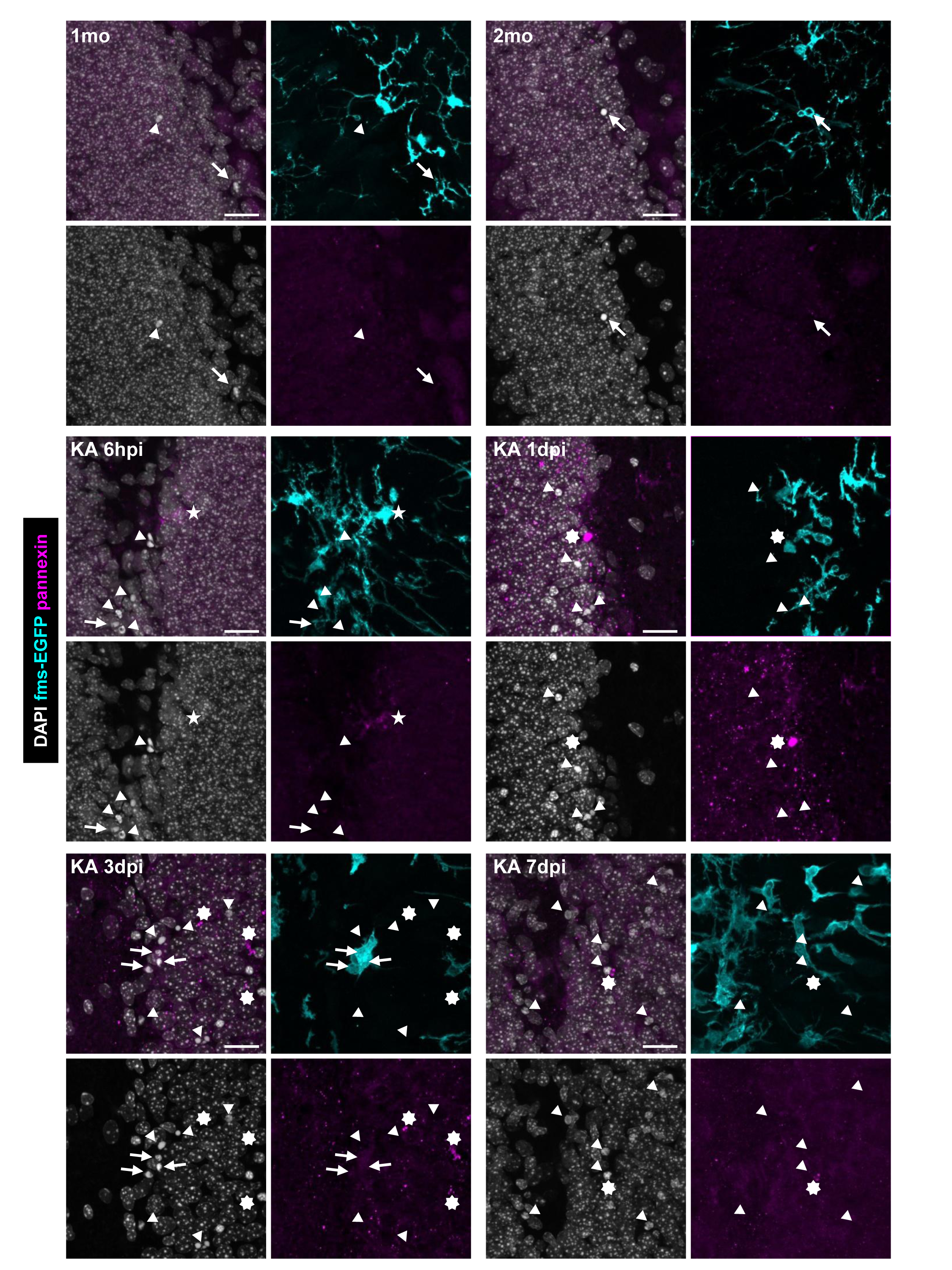

Supplement: S12 Fig — Representative confocal z-stack projections of the DG in control (1 and 2 mo) and KA-injected mice from 6 hpi to 7 dpi showing the low expression of pannexin (magenta) in granule neurons in the DG. Pannexin was expressed at low levels by granule neurons in control mice, and appeared in puncta on their surface along the time course after KA was injected (7-point stars), and could occasionally be diffusely expressed in microglia (5-point star at 6 hpi). Pannexin expression was largely absent in apoptotic cells, either phagocytosed (arrows) or nonphagocytosed (arrowheads) in control and KA mice. We found some cases of nonphagocytosed apoptotic cells labeled with puncta of pannexin at 7 dpi (7-point star at 7 dpi). Scale bars = 20 μm. z = 14.7 μm. (TIF) [file pbio.1002466.s025.tif]

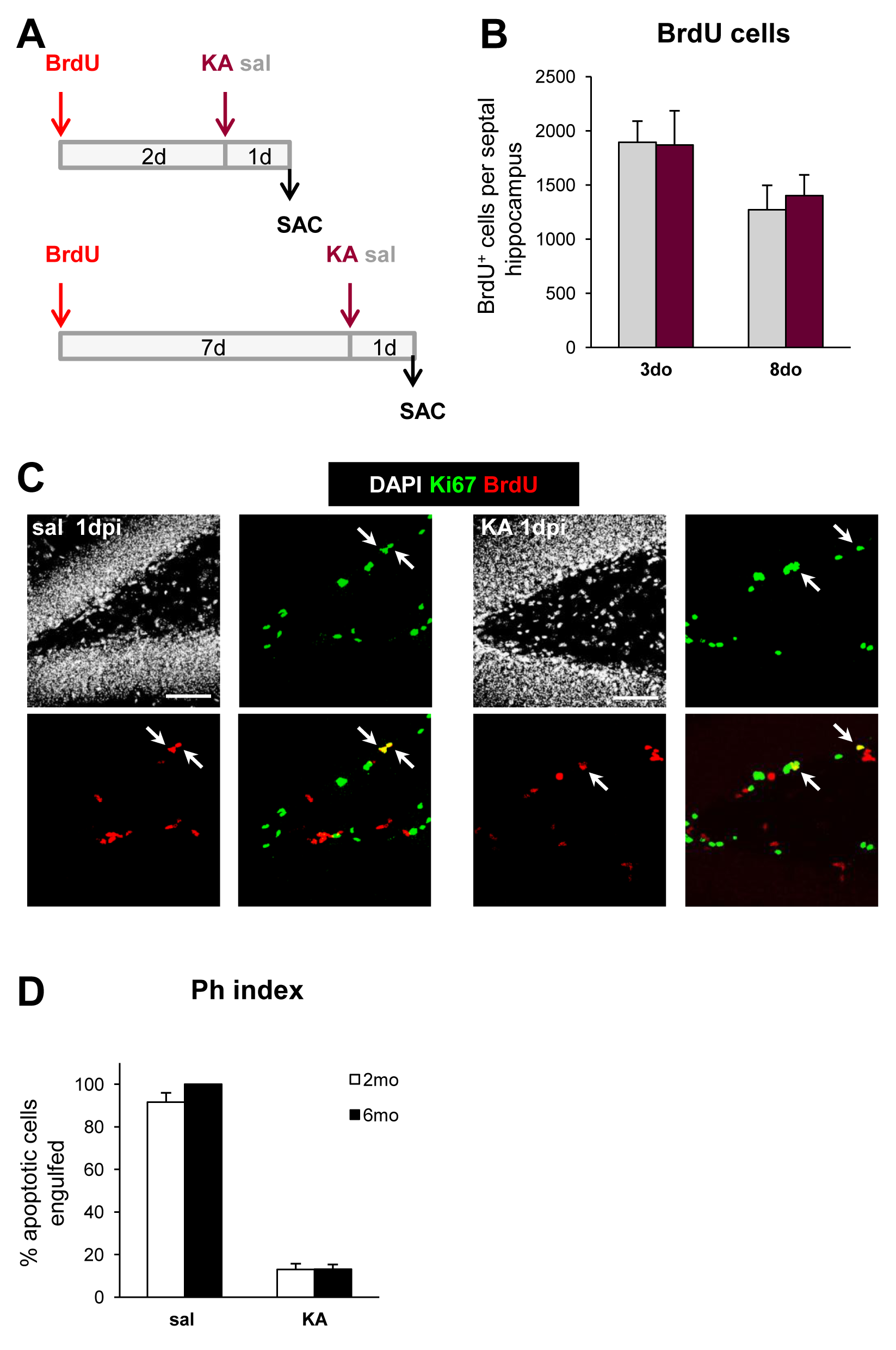

Supplement: S13 Fig — (A) Experimental design to test the effect of KA on 3 do (upper panel) and 8 do (lower panel). (B) Number of BrdU+ cells per septal hippocampus in saline or KA injected mice (n = 3–5 per group). 8 do cells were naturally less abundant than 3 do cells, reflecting the decreased survival of newborn cells. Nonetheless, KA did not significantly alter the number of BrdU+ cells born 3 or 8 d before. (C) Representative projections of confocal z-stacks of the DG of the hippocampus showing the colocalization between Ki67 (green) and BrdU (red), which had been injected 3 d before. The colocalization was a measure of the reentry of 3 do cells in the cell cycle and was identical in saline- and KA-injected mice. (C) Representative epifluorescent tiled image of the hippocampus and surrounding cortex of 2 and 6 mo mice injected with KA at 1 dpi stained with the neuronal activation marker c-fos. The same pattern of expression was found in young and mature mice throughout the DG, CA2, CA1, and the above cortex. (D) Ph index in the hippocampus (in % of apoptotic cells) in control and KA-injected mice at 2 and 6 mo (n = 4–5 per group). Scale bars = 50 μm (C). z = 28.7 μm (C, saline), 25.2 μm (C, KA). Underlying data is shown in S1 Data. (TIF) [file pbio.1002466.s026.tif]
