# Supplementary material for: Neuronal Hyperactivity Disturbs ATP Microgradients, Impairs Microglial Motility, and Reduces Phagocytic Receptor Expression Triggering Apoptosis/Microglial Phagocytosis Uncoupling
Source: PLoS Biol. 2016 May 26;14(5):e1002466. doi: 10.1371/journal.pbio.1002466 (PMC4881984; doi:10.1371/journal.pbio.1002466)
Supplement: S1 Table — List of biopsy (Cruces University Hospital, Bilbao, Spain) and autopsy (Netherlands Brain Bank, NBB) hippocampal samples analyzed, including the diagnostic (ND, nondemented controls), the number of sections analyzed, the total number of apoptotic cells, the number of apoptotic cells phagocytosed by microglia, the Ph index, as well as age, sex, PM (hr:min) delay, and cause of death. Representative images can be found in Fig 6and S8 Fig. (DOCX) [file pbio.1002466.s029.docx]

| **Code** | **Diagnostic** | **Procedence** | **Sections** | **Tot Apo** | **Phago Apo** | **Ph index** | **Age** | **Sex** | **PM delay** | **Cause of Death** |
| --- | --- | --- | --- | --- | --- | --- | --- | --- | --- | --- |
| MTLE030 | MTLE | biopsy (Cruces) | 3 | 15 | 8 | 53 | 38 | M | - | - |
| MTLE049 | MTLE | biopsy (Cruces) | 5 | 25 | 12 | 48 | 56 | M | - | - |
| MTLE052 | MTLE | biopsy (Cruces) | 2 | 20 | 9 | 45 | 46 | F | - | - |
| 2010-087 | Epilepsy | autopsy (NBB) | 1 | 25 | 1 | 4 | 76 | F | 3:45 | abdominal metastasis of ovary carcinoma |
| 2006-014 | ND control | autopsy (NBB) | 2 | 11 | 3 | 27 | 83 | F | 3:20 | euthanasia |
| 2009-022 | ND control | autopsy (NBB) | 2 | 25 | 2 | 8 | 77 | F | 2:55 | pulmonary metastasis of vulva carcinoma |
| 2008-081 | Epilepsy | autopsy (NBB) | 1 | 47 | 0 | 0 | 91 | M | 6:50 | acute heart failure |
| 1995-062 | ND control | autopsy (NBB) | 2 | 9 | 2 | 22 | 80 | M | 4:30 | renal insufficiency |
| 1996-052 | ND control | autopsy (NBB) | 2 | 8 | 2 | 25 | 73 | M | 9:10 | cardiac arrest |
| 1995-074 | Epilepsy | autopsy (NBB) | 2 | 20 | 0 | 0 | 77 | F | 16:00 | aspiration pneumonia |
| 1998-051 | ND control | autopsy (NBB) | 2 | 25 | 5 | 20 | 94 | F | 16:50 | thrombo-embolism |
| 1995-110 | ND control | autopsy (NBB) | 2 | 26 | 7 | 27 | 81 | F | 22:15 | coronary shock |
