# Supplementary material for: Neuronal Hyperactivity Disturbs ATP Microgradients, Impairs Microglial Motility, and Reduces Phagocytic Receptor Expression Triggering Apoptosis/Microglial Phagocytosis Uncoupling
Source: PLoS Biol. 2016 May 26;14(5):e1002466. doi: 10.1371/journal.pbio.1002466 (PMC4881984; doi:10.1371/journal.pbio.1002466)
Supplement: S2 Table — Percentage (in weight) in saturated, monounsaturated, omega 6 (Ω6) polyunsaturated, and omega 3 (Ω3) polyunsaturated fatty acids, as determined by gas chromatography. AA, arachidonic acid; ALA, α-linolenic acid; FAs, fatty acids; LA, linolenic acid; ND, not detected (under the limit for the detection by gas chromatography, <0.05%); PUFAs, polyunsaturated fatty acids. (DOCX) [file pbio.1002466.s030.docx]

| **Fatty acids** | **Ω3 balanced diet** | **Ω3 deficient diet** |
| --- | --- | --- |
| 16:0 | 22.6 | 7.3 |
| 18:0 | 3.3 | 4.1 |
| other saturated FAs | 1.8 | 1.6 |
| **total saturated FAs** | **27.7** | **13.0** |
| 16:1 Ω7 | 0.2 | 0.2 |
| 18:1 Ω9 | 57.9 | 28.1 |
| 18:1 Ω7 | 1.5 | 0.9 |
| other monounsaturated FAs | 0.4 | 0.2 |
| **total monounsaturated FAs** | **60.0** | **29.4** |
| 18:2 Ω6 (LA) | 10.6 | 57.4 |
| 20:4 n Ω6 (AA) | ND | ND |
| **total Ω6 polyunsaturated FAs** | **10.7** | **57.4** |
| 18:3 Ω3 (ALA) | 1.6 | 0.2 |
| **total Ω3 PUFAs** | **1.6** | **0.2** |
| **total PUFAs** | **12.3** | **57.6** |
