# Supplementary material for: Neuronal Hyperactivity Disturbs ATP Microgradients, Impairs Microglial Motility, and Reduces Phagocytic Receptor Expression Triggering Apoptosis/Microglial Phagocytosis Uncoupling
Source: PLoS Biol. 2016 May 26;14(5):e1002466. doi: 10.1371/journal.pbio.1002466 (PMC4881984; doi:10.1371/journal.pbio.1002466)
Supplement: S3 Table — List of primers used to amplify reference genes, cytokines, and glutamate receptor subunits. The gene name, Gene Bank accession number, amplicon size, sequence, and software used for their design are listed. (DOCX) [file pbio.1002466.s031.docx]

| **Gene** | **Gene Bank** | **Amplicon size** | **Sequence (5’-3’)** | **Ref.** |
| --- | --- | --- | --- | --- |
| **Reference genes** |  |  |  |  |
| OAZ1 | NM_008753 | 51 | Fwd AGCGAGAGTTCTAGGGTTGCC | Primer Express |
|  |  |  | Rev CCCCGGACCCAGGTTACTAC |  |
| L27A | BC086939 | 101 | Fwd TGTTGGAGGTGCCTGTGTTCT | Primer Express |
|  |  |  | Rev CATGCAGACAAGGAAGGATGC |  |
| HPRT |  | 150 | Fwd ACAGGCCAGACTTTGTTGGA  Rev ACTTGCGCTCATCTTAGGCT | Primer Blast |
| **Cytokines** |  |  |  |  |
| IL-1β | NM_008361 | 152 | Fwd CAACCAACAAGTGATATTCTCCATG | Primer Express |
|  |  |  | Rev GATCCACACTCTCCAGCTGCA |  |
| IL-6 | NM_031168 | 141 | Fwd GAGGATACCACTCCCAACAGACC | Primer Express |
|  |  |  | Rev AAGTGCATCATCGTTGTTCATACA |  |
| TGFβ1 | NM_011577 | 51 | Fwd GCAGTGGCTGAACCAAGGAG | Primer Express |
|  |  |  | Rev TGAGCGCTGAATCGAAAGC |  |
| TNFα | NM_013693 | 179 | Fwd CATCTTCTCAAAATTCGAGTGACAA | Primer Express |
|  |  |  | Rev TGGGAGTAGACAAGGTACAACCC |  |
| CSF | NM_007778 | 51 | Fwd GTCCTGCAGCAGTTGATCGA | Primer Express |
|  |  |  | Rev GGCAATCTGGCATGAAGTCTC |  |
| MIC | NM_011819 | 52 | Fwd TCAGTCCAGAGGTGAGATTGGG | Primer Express |
|  |  |  | Rev TTGACGCGGAGTAGCAGCTG |  |
| **AMPA receptors** |  |  |  |  |
| Gria1 | NM_001113325.2 | 111 | Fwd TACATTGAGCAACGCAAGCC | Primer Blast |
|  | NM_008165.4 |  | Rev GTTTACGGGACCTCTCAGGG |  |
|  | NM_001252403.1 |  |  |  |
| Gria2 | NM_001083806.1 | 148 | Fwd GGGGACAAGGCGTGGAAATA | Primer Blast |
|  | NM_013540.2 |  | Rev CCAATCTTCCGGGGTCCATT |  |
|  | NM_001039195.1 |  |  |  |
| Gria3 | NM_016886.4 | 107 | Fwd TGACAGCTCATCCTCCGAGA | Primer Blast |
|  | NM_001281929.1 |  | Rev AGCGCTCATTTCCTTCCAGT |  |
| Gria4 | NM_019691.4 | 135 | Fwd AAGCACGTCAAAGGCTACCA | Primer Blast |
|  | NM_001113180.1 |  | Rev TTTCGTCACCATGGGCGTAT |  |
|  | NM_001113181.1 |  |  |  |
| **Kainate receptors** |  |  |  |  |
| Grik1 | NM_146072.4 | 76 | Fwd CTCGCTTGCCTAGGAGTCAG | Primer Blast |
|  | NM_010348.3 |  | Rev GGTGGGGGTTATACCACTCG |  |
| Grik2 | NM_001111268.1 | 102 | Fwd CTTCTCTTCCCTCAGCCGTG | Primer Blast |
|  | NM_010349.2 |  | Rev TGCAAGCGAATGAGACCAGT |  |
| Grik3 | NM_001081097.2 | 131 | Fwd GTTCCTAGTGTGCGCCTTCT | Primer Blast |
|  |  |  | Rev ATCGAAAGGCGTGCTCTTCA |  |
| Grik4 | NM_175481.5 | 131 | Fwd TCCTGCTTGGCTCTTGATGG | Primer Blast |
|  |  |  | Rev CGCGGTTGATACGGTTCTTG |  |
| Grik5 | NM_008168.2 | 135 | Fwd TGATAGTCGCCTTCGCCAAT | Primer Blast |
|  |  |  | Rev TCCCATTGATCTGCTCTCGG |  |
| **NMDA receptors** |  |  |  |  |
| Grin1 | NM_008169.2 | 114 | Fwd GACACAAGGATGCCCGTAGG | Primer Blast |
|  | NM_001177656.1 |  | Rev TCTTTTTAGGGTCGGGCTCTG |  |
|  | NM_001177657.1 |  |  |  |
| Grin2a | NM_008170.2 | 148 | Fwd CGCATCCATGGCTTGGTGTTT | Primer Blast |
|  |  |  | Rev TGGATGTCGGATCCTTGTCAG |  |
| Grin2b | NM_008171.3 | 112 | Fwd CATGGGTGTCTGTTCTGGCA | Primer Blast |
|  |  |  | Rev GGGGGAGTTCATCACGGATT |  |
| Grin3a | NM_001276355.1 | 146 | Fwd TGGTACAAGGGGTTTCAGCG | Primer Blast |
|  | NM_001033351.1 |  | Rev GTGCAGGGGATTCTGACTCT |  |
| Grin3b | NM_130455.2 | 140 | Fwd TGTTTGTCCTGCTGTGCCT | Primer Blast |
|  |  |  | Rev GTGGATCTTCTGGCTCGTGTGAA |  |
| **Metabotropic receptors** |  |  |  |  |
| GRM1 | NM_016976.3 | 150 | Fwd AAACCCGAGAGGAATGTCCG  Rev GCCGTTAGAATGGCGTTCC | Primer Blast |
| GRM2 | NM_001160353.1 | 143 | Fwd TGTCAAGTTTGATGCCCCCT  Rev AGCCTACCTTCTGGTAGCGA | Primer Blast |
| GRM3 | NM_181850.2 | 73 | Fwd TTCACAGCTCCATTCAACCCA  Rev CCATCCCGTCTCCGTAAGTG | Primer Blast |
| GRM4 | NM_001013385.1 | 107 | Fwd CACCAAGCCTGAACGAGTGG  Rev CGTAGCTGATCTGGGGGATCTTG | Primer Blast |
| GRM5 | NM_001081414.2  NM_001143834.1 | 123 | Fwd ACAACCTCTACAGTGGTGCG  Rev GGAGCTTAGGGTTTCCCCAG | Primer Blast |
| GRM6 | NM_173372.2 | 105 | Fwd TCATCCCTCCCCAGAATCCTT  Rev ATCTGCTCCTGGACTGAGCC | Primer Blast |
| GRM7 | NM_177328.3 | 141 | Fwd CTCCAGGGCTGTCGTGATTT  Rev TGCTGATGCAGTGGGTTGAT | Primer Blast |
| GRM8 | NM_008174.2 | 142 | Fwd ACCCATATTCACCAAGCCCG  Rev GTCACTTAGCTCTGGGGCTG | Primer Blast |
| **Purinergic receptors** |  |  |  |  |
| P2X4 | NM_011026 | 52 | Fwd TAAGTATGTGGAAGACTACGAGCAGG  Rev TCACTGGTCCGTCTCTCCG | Primer Express |
| P2X7 | NM_011027  NM_001038845  NM_001038839 | 51 | Fwd ACTATACCACGAGAAACATCTTGCC  Rev GAAAGGTACAAGAGCCGTTCATAGTT | Primer Express |
| P2Y6 | NM_183168 | 82 | Fwd ACAGACTCTCCGAGCATAGGAAA  Rev GGCGGCAAGCCTGGA | Primer Express |
| P2Y12 | NM_027571 | 88 | Fwd GCAGAACCAGGACCATGGAT  Rev CTGACGCACAGGGTGCTG | Primer Express |
| **Phagocytic receptors** |  |  |  |  |
| TREM2 | NM_031254.3  NM_001272078.1 | 110 | Fwd CTGATCACAGCCCTGTCCCAA  Rev CGTCTCCCCCAGTGCTTCAA | Primer Blast |
| CR3 | NM_001082960.1  NM_008401.2 | 100 | Fwd AATTGAGGGCACGCAGACA  Rev GCCCAGCAAGGGACCATTAG | Primer Blast |
| MerKT | NM_008587.1 | 131 | Fwd AAGGTCCCCGTCTGTCCTA  Rev GCGGGGAGGGGATTACTTTG | Primer Blast |
| GRP34 | NM_011823.4 | 151 | Fwd CTTCAGGAAAGCTTCAACTC  Rev GTAACTATCAGGAGGAGAGC | Sigma |
